# Enantiopure Trisubstituted Tetrahydrofurans with Appendage Diversity: Vinyl Sulfone- and Vinyl Sulfoxide-Modified Furans Derived from Carbohydrates as Synthons for Diversity Oriented Synthesis

**DOI:** 10.3390/molecules21060690

**Published:** 2016-05-26

**Authors:** Debanjana Dey, Tanmaya Pathak

**Affiliations:** Department of Chemistry, Indian Institute of Technology Kharagpur, Kharagpur 721302, India; debanjanadey@gmail.com

**Keywords:** vinyl sulfone, vinyl sulfoxide, modified tetrahydrofuran, Michael addition, diatereoselectivity

## Abstract

Enantiomerically pure 2-substituted-2,5-dihydro-3-(aryl) sulfonyl/sulfinyl furans have been prepared from the easily accessible carbohydrate derivatives. The orientation of the substituents attached at the C-2 position of furans is sufficient to control the diastereoselectivity of the addition of various nucleophiles to the vinyl sulfone/sulfoxide-modified tetrahydrofurans, irrespective of the size of the group. The orientation of the substituents at the C-2 center also suppresses the influence of sulfoxides on the diastereoselectivity of the addition of various nucleophiles. The strategy leads to the creation of appendage diversity, affording a plethora of enantiomerically pure trisubstituted furanics for the first time.

## 1. Introduction 

In order to increase the efficiency of a synthetic strategy for creating appendage, stereochemical, and scaffold diversities, explosive growth has taken place in the area of diversity-oriented synthesis (DOS) [1,2,3,4,5,6,7,8,9,10,11,12]. However, the imposition of stereocontrol in DOS remains a most difficult task. The asymmetric version of multicomponent reactions, considered as the cornerstone of DOS [4,7], has started emerging only recently [13]. It would be logical to use enantiopure substrates for the generation of new appendage or skeletal diversities with defined stereogenic centers in DOS, but in reality only a limited number of enantiopure “chiral pool” substrates such as carbohydrates have been utilized [2,3,7,8,13].

**Figure 1:**
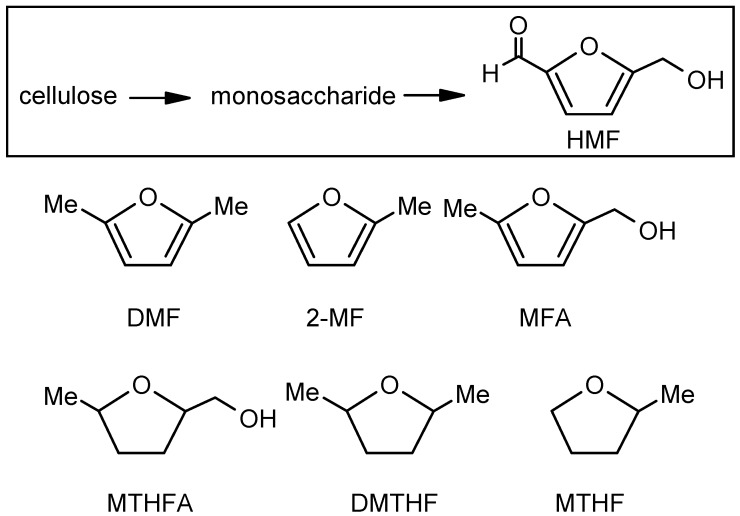
Selected examples of biomass-derived “petrochemicals”.

Carbohydrates as the source of chirality carbons are thoroughly underutilized and understudied in DOS [13], although carbohydrates are by far the most abundant organic compounds on earth. Carbohydrates represent the major portion of the annually renewable biomass [14,15], and it is estimated that, in 2025, up to 30% of raw materials for the chemical industry will be produced from renewable sources such as biomass [16]. As a result of continued efforts, 2,5-dimethylfuran (DMF) [17], a molecule with the potential of an alternative fuel, is now generated from the easily available biomass precursor d-fructose via 5-hydroxymethylfurfural (HMF). However, the conversion of biomass is not restricted to use as an alternative fuel alone. Recent research in this area is generating new tetrahydrofuran derivatives (furanics) such as 2,5-dimethyltetrahydrofuran (DMTHF), 2-methylfuran (2-MF), methyltetrahydrofuran (MTHF), 2-methylfurfural alcohol (MFA), 5-methyltetrahydrofurfural alcohol (MTHFA) *etc*. from DMF (Figure 1) [18].

Since substituted tetrahydrofuran moiety occurs extensively in natural and synthetic compounds, synthetic approaches to access this class of oxaheterocycles, especially in an enantiopure form, has proliferated during last several decades, and the overall work has been reviewed [19,20]. As part of a program for the generation of enantiomerically pure non-carbohydrate chemicals from easily available carbohydrates, we developed a DOS-based strategy for the construction of enantiopure furofurans from vinyl sulfone-modified mono- as well as bicylic-carbohydrates [21,22,23]. In such a synthesis, our tool was to use highly reactive vinyl sulfone [24,25,26,27,28,29] and vinyl sulfoxide [30,31,32] functional groups that are known as powerful Michael acceptors and efficient partners in Diels–Alder reactions.

**Figure 2:**
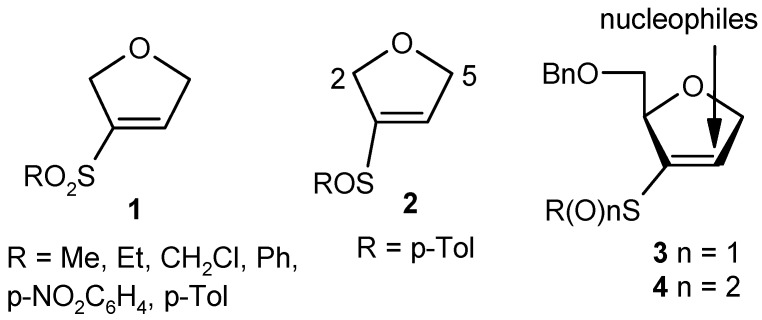
Representative examples of vinyl sulfone- and vinyl sufoxide-modified tetrahydrofurans.

Interestingly, 2,5-dihydro-3-(alkyl/aryl sulfonyl) furans **1** (Figure 2), employed long ago, are the special class of cyclic vinyl sulfone which underwent cycloaddition as well as Michael addition reactions with various nucleophiles [33,34,35]. 2,5-dihydro-3-(alkyl/aryl sulfinyl) furans **2** (Figure 2) are also important in synthetic chemistry due to their ability to participate in the Michael addition reactions. Since it is well-known that unsaturated sulfoxides have the potential to act as chiral auxiliaries in asymmetric synthesis, Compound **2** has the added advantage of inducing asymmetric induction. [36,37,38,39,40] Although these cyclic Michael acceptors have high potential as synthetic intermediates [33,34,35,36,37,38,39,40], there are scattered publications, few in number, on the synthesis and utility of 2,5-dihydro-3-(alkyl/aryl) sulfonyl/sulfinyl furans (Figure 2) [23,41,42,43,44,45,46,47]. To date, these compounds are thoroughly understudied and underutilized, partly because these molecules are difficult to access in reasonably large amount using conventional synthetic strategies.

## 2. Result and Discussion

2-benzyloxymethyl-2,5-dihydro-3-(*p*-tolyl sulfonyl)- and 2-benzyloxymethylene-2,5-dihydro-3-(*p*-tolyl sulfinyl)-furans **3** and **4** (Figure 2) undergoes nucleophilic attack at C-4 position from the -side of the tetrahydrofuran ring in such a fashion that all substituents attached to the furan ring occupy *anti*-orientations [23]. In order to broaden the scope of converting carbohydrates to furanics, we examined whether the steric bulk at C-2 position of furans would play the deciding role in determining the diastereoselectivity of the addition of nucleophiles to vinyl sulfones and vinyl sulfoxides structurally close to **3** and **4**. Therefore, two vinyl sulfone-modified tetrahydrofurans, one with a “small” methyl group at the C-2 position of the furan ring and another with the “large” -CH_2_OTr group at the same position, were prepared and subjected to addition reactions. 

**Scheme 1:**
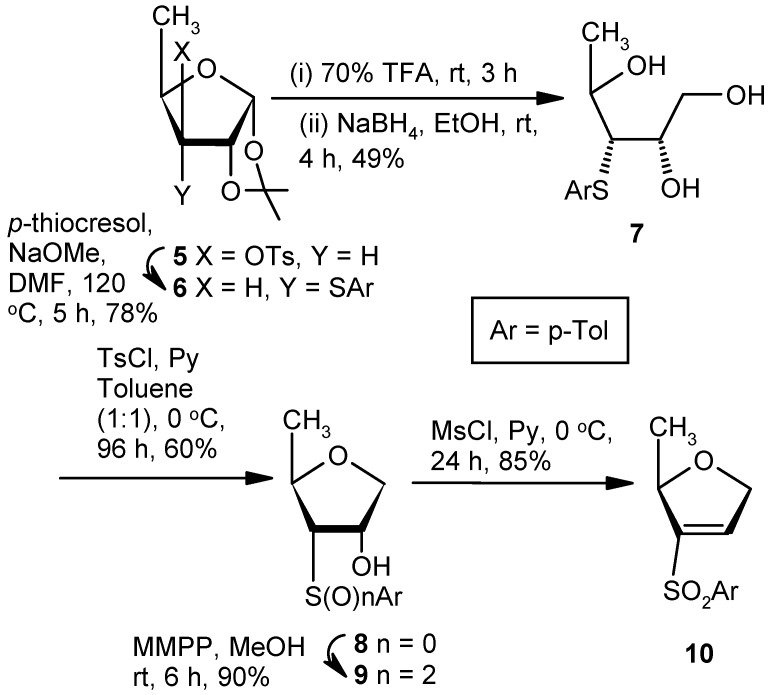
Synthesis of vinyl sulfone-modified tetrahydrofyran **10**.

Thus, the preparation of vinyl sulfone-modified tetrahydrofuran **10** with a methyl group at C-2 position started from Compound **5** (Scheme 1) [45]. The tosyl compound **5** was heated with *p*-thiocresol in the presence of NaOMe at 120 °C to afford the sulfide (**6**). Compound **6** was consecutively treated with trifluoroacetic acid (TFA) and sodium borohydride (NaBH_4_) to afford the acyclic compound **7**. Selective tosylation of Compound **7** afforded the desired enantiomerically pure cyclic compound **8**. Oxidation of **8** with magnesium monoperoxyphthalate hexahydrate (MMPP) afforded the sulfone compound **9**. The hydroxyl group of **9** was mesylated, and the subsequent elimination of the mesyl group produced the desired vinyl sulfone **10** (Scheme 1). The vinylic proton of **10** at δ 7.17 (^1^H-NMR) and the corresponding carbon at δ 138.3 (^13^C-NMR) confirmed the formation of the vinyl sulfone moiety of Compound **10**. 

**Scheme 2:**
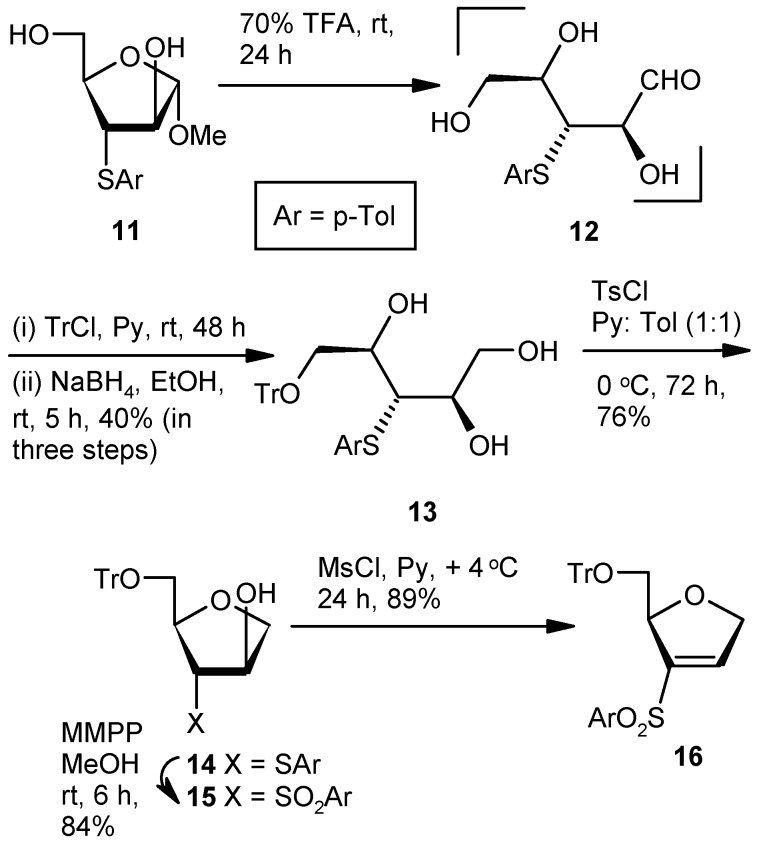
Synthesis of vinyl sulfone-modified tetrahydrofuran **16**.

The vinyl sulfone-modified tetrahydrofuran **16**, having a bulky group like -CH_2_OTr group at C-2 position, was obtained as follows. α-Anomeric *arabino* sulfide **11** [48,49] was treated with 70% TFA in water to afford the acyclic aldehyde **12**. The aldehyde was directly treated with trityl chloride in pyridine to selectively protect the primary alcohol; the crude material was reduced with NaBH_4_ in ethanol to produce the trityl protected acyclic sulfide **13** in two steps. Selective tosylation of **13** afforded the cyclic compound **14**. Oxidation of **14** produced the corresponding sulfone **15**, which, after mesylation, produced the desired tritylated vinyl sulfone **16** (Scheme 2). The vinylic proton of **16** at δ 7.00 and the corresponding carbon at δ 140.8 confirmed the formation of the vinyl sulfone moiety of Compound **16**.

**Scheme 3:**
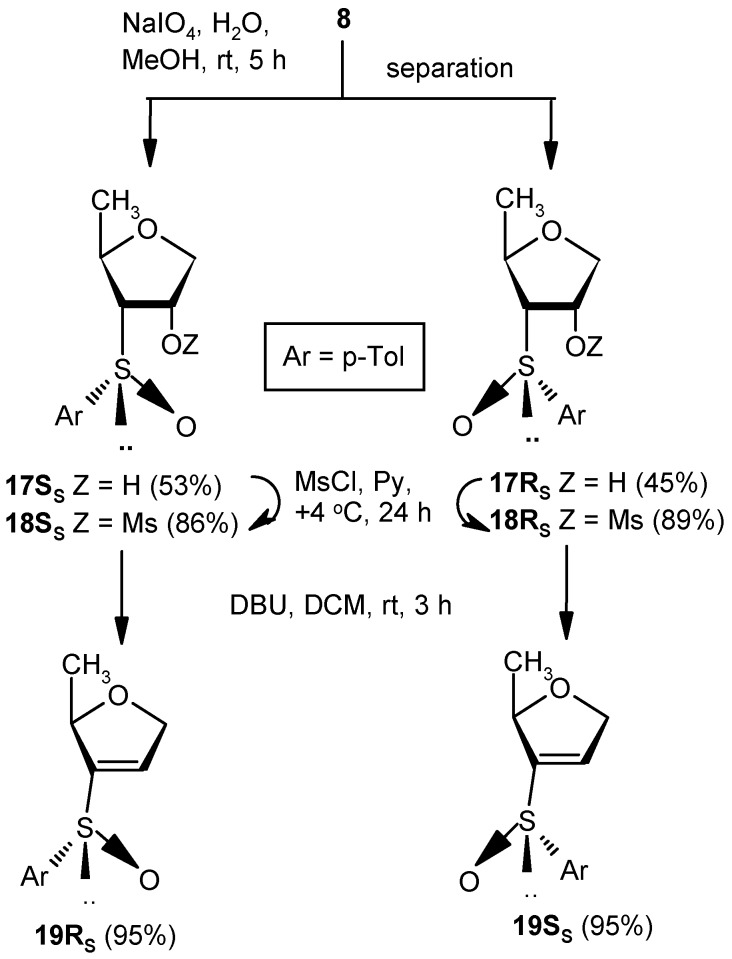
Synthesis of vinyl sulfoxide-modified tetrahydrofurans **19R_S_**/**19S_S._**

**Figure 3:**
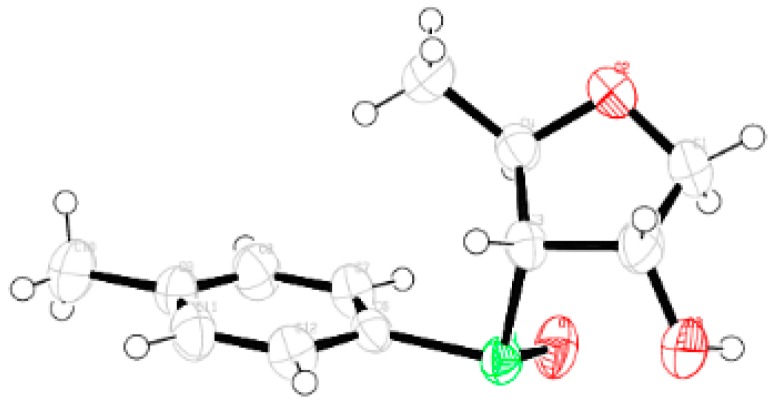
Crystal structure of **17S_S._**

To synthesize the corresponding vinyl sulfoxides, Compound **8** was oxidized under controlled condition [31] using NaIO_4_/MeOH-H_2_O to afford the sulfoxides. Two sulfoxides **17S_S_** and **17R_S_**, formed almost in a 1:1 ratio, were separated. The structure of Sulfoxide **17S_S_** was confirmed by X-ray crystallography (Figure 3), which indirectly confirmed the structure of **17R_S_**. The sulfoxides were separately mesylated to afford **18S_S_** and **18R_S_**, respectively, which were treated with DBU in DCM at room temperature for 3 h to afford desired vinyl sulfoxides **19R_S_** and **19S_S_**, respectively, in excellent yields (Scheme 3). The crystal structure of sulfoxide **17S_S_** (Figure 3) also indirectly confirmed the structure of the corresponding vinyl sulfoxide **19R_S_**.

**Scheme 4:**
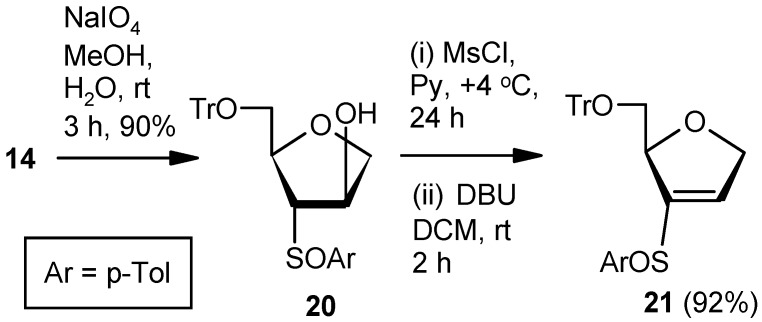
Synthesis of vinyl sulfoxide-modified terahydrofurans **21**.

The absolute configuration at the sulfur of vinyl sulfoxides **19R_S_** and **19S_S_** could also be confirmed by comparing the NMR data of **19** with those reported for 2-aryl-3-sulfinyl-2,5-dihydrofuran [23,50]. In the reported data, the vinylic proton was used as a tool for assigning the stereochemistry of sulfur atom because of the highly deshielding effect induced by the sulfinyl oxygen on the vinylic hydrogen [50]. The vinylic proton of Compound **19R_S_** appeared at δ 6.59 in its ^1^H-NMR spectrum, whereas that for Compound **19S_S_** appeared at δ 6.52. Thus, it was clear that the chemical shift value of the vinylic proton of **19Rs** was much more deshielded than that of **19S_S_**. According to the reported data [50], the higher chemical shift value of the vinylic proton is possible if sulfur oxygen is oriented towards the vinylic proton. It was therefore clear that in Compound **19S_S_** the sulfur oxygen was oriented opposite the vinylic proton. The corresponding trityl protected vinyl sulfoxides were synthesized from the cyclic sulfide (**14**) via sulfoxides (**20**). These sulfoxides could not be separated at this stage and therefore were directly converted to vinyl sulfoxides (**21**) in excellent yields, once again as an inseparable mixture (Scheme 4); two vinylic protons appeared at δ 6.50 and δ 6.81, confirming the formation of the vinyl sulfoxide group.

**Scheme 5:**
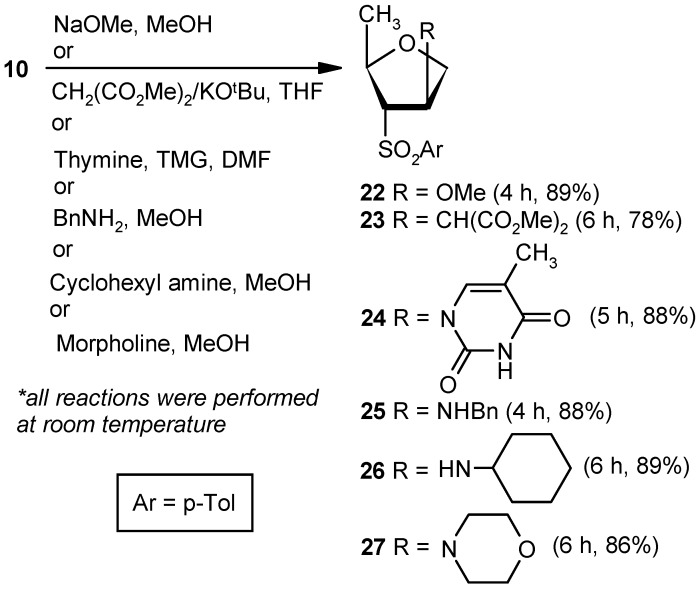
Synthesis of enantiopure furanics from **10**.

**Figure 4:**
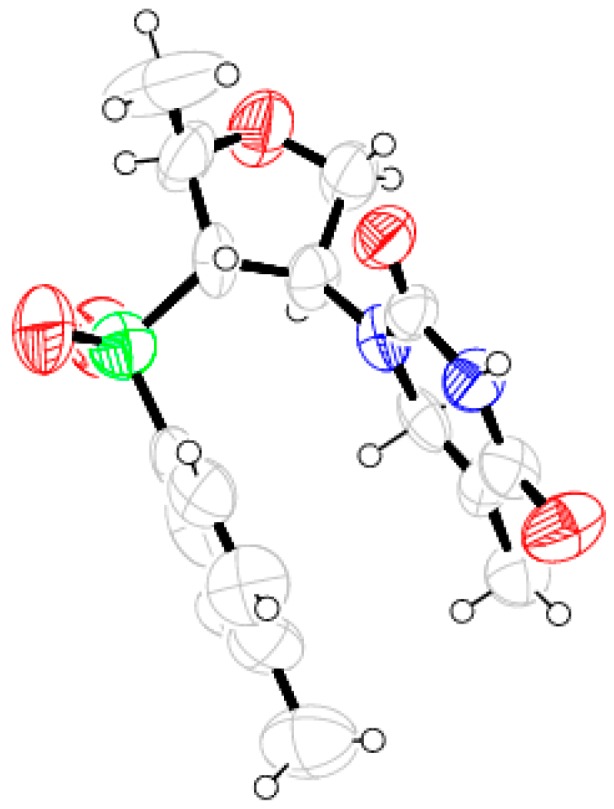
Crystal structure of **24**.

**Figure 5:**
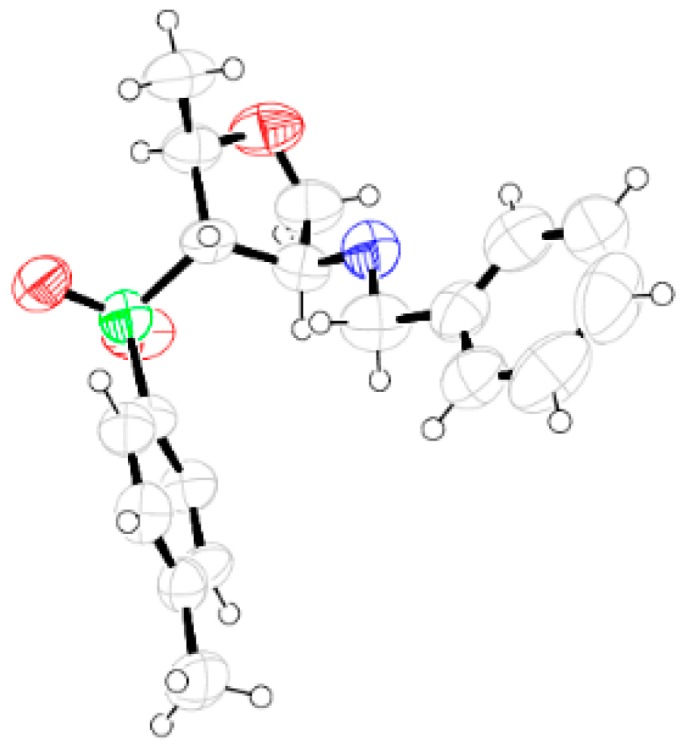
Crystal structure of **25**.

**Scheme 6:**
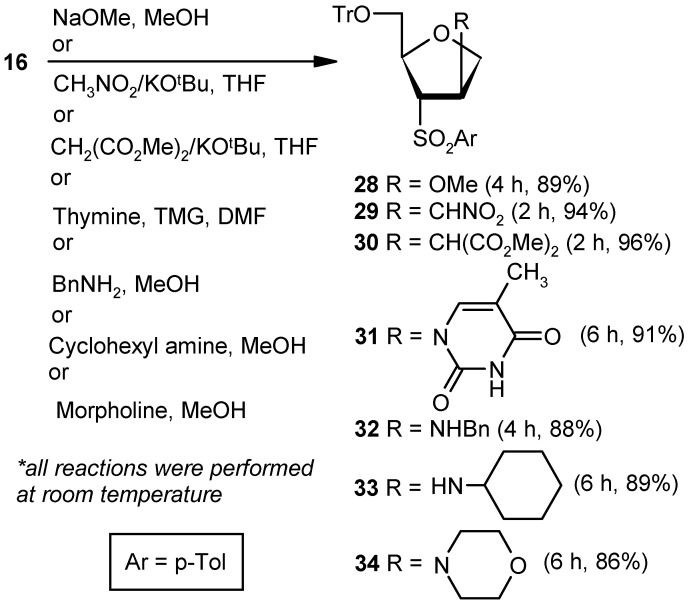
Synthesis of enantiopure furanics from **16**.

Vinyl sulfone **10** was reacted with sodium methoxide/methanol, dimethylmalonate/KO^t^Bu, thymine/TMG, benzylamine, cyclohexylamine, and morpholine at room temperature to afford the Michael-adducts **22**–**27**, respectively (Scheme 5). The spectral data of all these compounds were found to be similar to the spectra of compounds obtained from **4** [23]. However, the structures **24** and **25** were confirmed by X-ray crystallography (Figure 4 and Figure 5). Thus, it was clear that all the addition compounds in Scheme 5 were in “*arabino”* configuration. The tritylated vinyl sulfone-modified tetrahydrofuran **16** was also reacted with sodium methoxide/MeOH, nitromethane/KO^t^Bu, dimethylmalonate/KO^t^Bu, thymine/TMG, benzylamine, cyclohexylamine, and morpholine to afford the single diastereomers **28**–**34**, respectively (Scheme 6). Once again, the spectral data established the similarity between the Michael adducts of **4** and Compounds **22**–**27**.

**Scheme 7:**
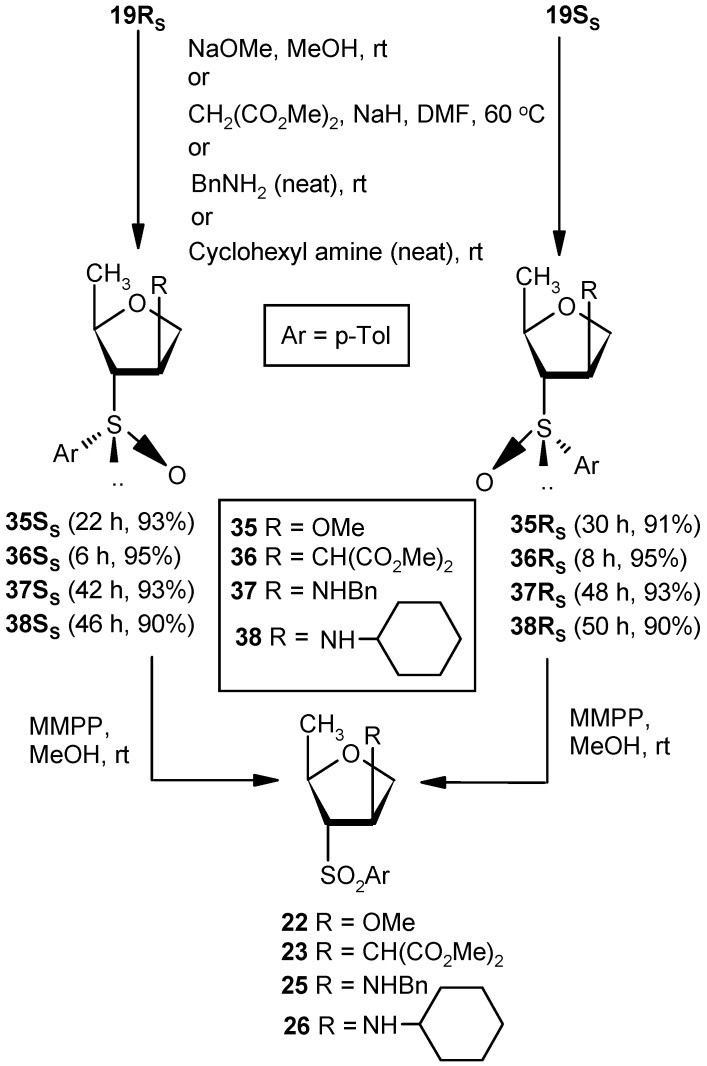
Synthesis of enantiopure furanics from **19R_S_** and **19S_S_**.

**Scheme 8:**
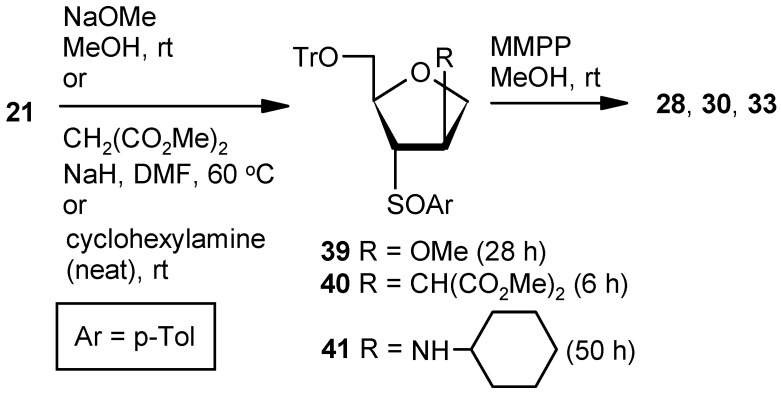
Synthesis of enantiopure furanics from tritylated vinyl sulfoxides **21**.

To identify the asymmetric induction by the sulfoxide group, if any, vinyl sulfoxide-modified tetrahydrofurans were treated with sodium methoxide/MeOH, dimethylmalonate/NaH, benzylamine, and cyclohexylamine. Thus, **19R_S_** afforded single diastereomers **35S_S_**–**38S_S_**, respectively, and **19S_S_** afforded **35R_S_**–**38R_S_**, respectively (Scheme 7). All these Michael-adduct pairs, **35S_S_**/**35R_S_**, **36S_S_**/**36R_S_**, **37S_S_**/**37R_S,_** and **38S_S_**/**38R_S_** were separately oxidized with MMPP in MeOH to afford **22**–**26**, respectively, (Scheme 7). The oxidation reactions of amino compounds were terminated within 0.5 h to avoid over-oxidation. The tritylated vinyl sulfoxides **21** were also reacted with a selected group of nucleophiles, namely sodium methoxide/MeOH, dimethylmalonate/NaH, and cyclohexylamine. The products, **39**–**41** were inseparable and therefore those individual mixtures were directly oxidized to the corresponding sulfones **28**, **30**, and **33** in good overall yields (Scheme 8).

## 3. Experimental Section

### General Methods

All reactions were conducted in a N_2_ atmosphere. Melting points were determined in open-end capillary tubes and are uncorrected. Carbohydrates and other fine chemicals were obtained from commercial suppliers and are used without purification. Solvents were dried and distilled following the standard procedures. TLC was carried out on pre-coated plates (Merck silica gel 60, f_254_, Merck, Darmstadt, Germany), and the spots were visualized with UV light or by charring the plate dipped in a 5% H_2_SO_4_–MeOH solution. Column chromatography was performed on silica gel (230–400 mesh). ^1^H- and ^13^C-NMR for new compounds were recorded at 200/400 and 50/100 MHz, respectively, using CDCl_3_ as the solvent in Bruker (Massachusetts, MA, USA) NMR instrument DEPT experiments had been carried out to identify the methylene carbons. Optical rotations were recorded at 589 nm. Mass spectroscopy data were obtained from a mass analyzer (Xevo G2 QTof) consisting of TOF and quadrupole in either ESI^+^ or ESI^−^ mode. The electronic information of compounds is available in the Supplementary Materials. 

Compound **6**: A solution of *p*-thiocresol (2.83 g, 22.85 mmol) and NaOMe (0.98 g, 18.28 mmol) in anhyd DMF (10 mL) was stirred at room temperature for 0.5 h in a nitrogen atmosphere. A solution of Compound **5** (1.5 g, 4.57 mmol) in anhyd DMF (5 mL) was added, and the final solution was heated at 120 °C After 5 h (tlc), the reaction mixture was cooled to room temperature, poured into cold satd. aqueous solution of NaHCO_3_, and the product was extracted with EtOAc (3 × 10 mL). The combined organic layers were dried over anhyd Na_2_SO_4_ and filtered, and the filtrate was concentrated under reduced pressure. The crude material was purified over silica gel to afford **6** (0.99 g, 78%). Eluent: EtOAc: PE (1:9); yellowish gum; ${\left[\alpha \right]}_{D}^{23.5}$ = (+) 37.8 (*c* = 1.02, CHCl_3_); ^1^H-NMR (400 MHz, CDCl_3_): δ = 1.29 (d, *J* = 6.0 Hz, 3H), 1.38 (s, 3H), 1.58 (s, 3H), 2.34 (s, 3H), 2.88 (dd, *J* = 4.4, 10.0 Hz, 1H), 4.10–4.14 (m, 1H), 4.77–4.79 (m, 1H), 5.80 (d, *J* = 4.0 Hz, 1H), 7.12 (d, *J* = 7.6 Hz, 2H), 7.41 (d, *J* = 8.0 Hz, 2H); ^13^C-NMR (100 MHz, CDCl_3_): δ = 17.5, 21.0, 26.3, 26.4, 57.7, 77.5, 81.5, 103.8, 111.8, 129.8, 131.1, 132.3, 137.5; HRMS [ES^+^, (M + Na)^+^]: for C_15_H_20_O_3_NaS obsd 303.1056, calcd 303.1031.

Compound **7**: A mixture of Compound **6** (1.0 g, 3.57 mmol) and 70% trifluoroacetic acid in water (10 mL) was stirred at room temperature. After 3 h, (tlc) the reaction mixture was poured into ice-cold satd. Aqueous solution of NaHCO_3_, and the product was extracted with EtOAc (3 × 10 mL). The combined organic layers were dried over anhyd Na_2_SO_4_ and filtered, and the filtrate was concentrated under reduced pressure to get a residue. The residue was dissolved in EtOH (15 mL), and NaBH_4_ (0.55 g, 14.28 mmol) was added at 0 °C. After 4 h at room temperature, the reaction mixture was concentrated under reduced pressure to get a residue. The residue was poured into satd. Aqueous solution of NaHCO_3_, and the product was extracted with EtOAc (3 × 10 mL). The combined organic layers were dried over anhyd Na_2_SO_4_ and filtered, and the filtrate was concentrated under reduced pressure. The residue was purified over silica gel to get **7** (0.42 g, 49%). Eluent: EtOAc: PE (2:3); yellowish gum; ${\left[\alpha \right]}_{D}^{23.5}$ = (−)14.6 (*c* = 1.02, CHCl_3_); ^1^H-NMR (400 MHz, CDCl_3_): δ = 1.35 (d, *J* = 6.4 Hz, 3H), 2.32 (s, 3H), 2.50 (bs, 1H), 3.17–3.20 (m, 2H), 3.30 (s, 1H), 3.71–3.75 (m, 1H), 3.88–3.90 (m, 2H), 4.16 (t, *J* = 6.0 Hz, 1H), 7.11 (d, *J* = 7.6 Hz, 2H), 7.36 (d, *J* = 7.6 Hz, 2H); ^13^C-NMR (100 MHz, CDCl_3_): δ = 21.0, 21.0, 60.6, 64.5 (CH_2_), 68.4, 72.9, 130.1, 130.7, 132.2, 137.8; HRMS [ES^+^, (M + Na)^+^]: for C_12_H_18_O_3_NaS obsd 265.0850, calcd 265.0874.

Compound **8**: A solution of tosylchloride (0.6 g, 3.09 mmol) in anhyd tolune (5 mL) was dropwise added to a solution of **7** (0.5 g, 2.06 mmol) in a mixture of anhyd pyridine and toluene (1:1; 5 mL) at 0 °C. The mixture was stirred for 0.5 h at 0 °C, and the reaction mixture was stored at +4 °C for 96 h. The mixture was filtered through celite, and the filtrate was evaporated to dryness. Residual pyridine was co-evaporated with toluene. The residue was purified over silica gel to afford **8** (0.28 g, 60%). Eluent: EtOAc: PE (1:4); white solid; Mp = 97 °C; ${\left[\alpha \right]}_{D}^{23.5}$ = (+) 98.6 (*c* = 1.01, CHCl_3_); ^1^H-NMR (400 MHz, CDCl_3_): δ = 1.38 (d, *J* = 6.4 Hz, 3H), 2.33 (s, 3H), 3.0 (s, 1H), 3.10 (dd, *J* = 4.8, 10.4 Hz, 1H), 3.80–3.86 (m, 2H), 4.10 (dd, *J* = 4.4, 10.0, 1H), 4.25 (s, 1H), 7.13 (d, *J* = 8.0 Hz, 2H), 7.34 (d, *J* = 8.0 Hz, 2H); ^13^C-NMR (100 MHz, CDCl_3_): δ = 19.0, 21.0, 61.4, 70.9, 73.7 (CH_2_), 76.4, 129.8, 130.1, 131.8, 137.9; HRMS [ES^+^, (M + H)^+^]: for C_12_H_17_O_2_S obsd 225.0920, calcd 225.0949.

Compound **9**: MMPP (3.3 g, 6.69 mmol) was added to a solution of 8 (0.5 g, 2.23 mmol) in MeOH (8 mL), and the mixture was stirred at room temperature. After 6 h (tlc), the mixture was evaporated under reduced pressure. The solid residue was stirred in a mixture of EtOAc and satd. aqueous NaHCO_3_ solution for 1 h. The organic part was separated, dried over anhyd Na_2_SO_4_, and filtered, and the filtrate was evaporated to dryness. The residue was purified over silica gel to afford **9** (0.51 g, 90%). Eluent: EtOAc: PE (2:3); white solid; Mp = 110 °C; ${\left[\alpha \right]}_{D}^{23.5}$ = (+) 26.2 (*c* = 0.98, CHCl_3_); ^1^H-NMR (400 MHz, CDCl_3_): δ = 1.26 (d, *J* = 6.4 Hz, 3H), 2.44 (s, 3H), 3.23–3.26 (m, 1H), 3.65 (d, *J* = 5.2 Hz, 1H), 3.75 (dd, *J* = 2.8, 10.0 Hz, 1H), 3.94 (dd, *J* = 4.4, 10.0 Hz, 1H), 4.49–4.58 (m, 2H), 7.37 (d, *J* = 8.4 Hz, 2H), 7.83 (d, *J* = 8.0 Hz, 2H); ^13^C-NMR (100 MHz, CDCl_3_): δ = 21.0, 21.6, 72.3, 72.4, 73.8, 73.9 (CH_2_), 128.3, 130.0, 136.3, 145.4; HRMS [ES^+^, (M + Na)^+^]: for C_12_H_16_O_4_NaS obsd 279.0691, calcd 279.0667.

Compound **10**: A solution of mesylchloride (0.5 mL, 5.85 mmol) in anhyd pyridine (3 mL) was added dropwise to a solution of **9** (0.5g, 1.95 mmol) in anhyd pyridine (5 mL). The mixture was stirred for 0.5 h and stored at +4 °C. After 24 h (tlc), the reaction mixture was poured into ice-cold water, and the product was extracted with EtOAc. The organic layer was dried over anhyd Na_2_SO_4_ and filtered, and the filtrate was evaporated to dryness. The residual pyridine was co-evaporated with toluene. The residue was purified over silica gel to afford **10** (0.39 g, 85%). Eluent: EtOAc: PE (1:4); yellowish gum; ${\left[\alpha \right]}_{D}^{23.5}$ = (−) 11.6 (*c* = 1.36, CHCl_3_); ^1^H-NMR (400 MHz, CDCl_3_): δ =1.31 (d, *J* = 6.4 Hz, 3H), 2.43 (s, 3H), 4.61–4.78 (m, 2H), 4.89–4.93 (m, 1H), 7.17 (s, 1H), 7.34 (d, *J* = 8.4 Hz, 2H), 7.76 (d, *J* = 8.4 Hz, 2H); ^13^C-NMR (100 MHz, CDCl_3_): δ = 20.9, 21.6, 73.3 (CH_2_), 80.5, 127.9, 130.0, 136.6, 138.3, 145.0, 145.3; HRMS [ES^+^, (M + H)^+^]: for C_12_H_15_O_3_S obsd 239.0729, calcd 239.0742.

Compound **13**: A mixture of Compound **11** (1.5 g, 1.11 mmol) and aqueous 70% trifluoroacetic acid (10 mL) was stirred at room temperature. After 24 h (tlc), the reaction mixture was poured into the ice-cold satd. aqueous solution of NaHCO_3,_ and the product was extracted with EtOAc (3 × 10 mL). The combined organic layers were dried over anhyd Na_2_SO_4_ and filtered, and the filtrate was concentrated under reduced pressure to get the residue **12**. The residue was dissolved in pyridine, and tritylchloride (0.93 g, 3.33 mmol) was added. The mixture was stirred at room temperature in an inert atmosphere. After 48 h (tlc), the reaction mixture was poured into ice-cold satd. aqueous solution of NaHCO_3,_ and the product was extracted with EtOAc (3 × 10 mL). Organic layers were pooled, dried over anhyd Na_2_SO_4_, and filtered, and the filtrate was concentrated under reduced pressure to get a residue. The residue was dissolved in EtOH (40 mL) and NaBH_4_ (0.17 g, 4.44 mmol) was added at 0 °C. After 5 h at room temperature, the reaction mixture was concentrated under reduced pressure. The residue was poured into satd. aqueous solution of NaHCO_3_, and the product was extracted with EtOAc (3 × 10 mL). The combined organic layers were dried over anhyd Na_2_SO_4_ and filtered, and the filtrate was concentrated under reduced pressure. The residue was purified over silica gel to afford **13** (1.11 g, 40%). Eluent: EtOAc: PE (3:2); yellowish gum; ${\left[\alpha \right]}_{D}^{27}$ = (−) 7.6 (*c* = 1.32, CHCl_3_); ^1^H-NMR (200 MHz, CDCl_3_): δ = 2.29 (s, 3H), 3.28–3.34 (m, 2H), 3.40–3.47 (m, 1H), 3.61–3.68 (m, 1H), 3.80–3.90 (m, 1H), 4.00–4.10 (m, 2H), 7.00 (d, *J* = 15.6 Hz, 2H), 7.17–7.36 (m, 16H); ^13^C-NMR (50 MHz, CDCl_3_): δ = 21.3, 54.8, 64.8 (CH_2_), 65.8 (CH_2_), 71.9, 72.9, 87.4, 127.4, 128.1, 128.8, 130.1, 131.3, 132.2, 137.5, 143.8 (3 × C); HRMS [ES^+^, (M + Na)^+^]: for C_31_H_32_O_4_NaS obsd 523.1964, calcd 523.1919.

Compound **14**: Compound **13** (1.0 g, 2.0 mmol) was converted to 14 (0.73 g, 76%) following the procedure described for the preparation of **8**. Eluent: EtOAc: PE (1:4); colorless gum; ${\left[\alpha \right]}_{D}^{27}$ = (+) 13.8 (*c* = 1.7, CHCl_3_); ^1^H-NMR (200 MHz, CDCl_3_): δ = 2.29 (s, 3H), 3.10 (dd, *J* = 3.0, 10.4 Hz, 1H), 3.42 (d, *J* = 3.8 Hz, 2H), 3.61 (dd, *J* = 2.6, 10.4 Hz, 1H), 3.89–4.12 (m, 4H), 7.04 (d, *J* = 7.8, 2H), 7.16–7.46 (m, 18H); ^13^C-NMR (50 MHz, CDCl_3_): δ = 21.2, 54.6, 64.8 (CH_2_), 74.7 (CH_2_), 77.4, 83.2, 87.9, 127.4, 128.1, 128.9, 130.1, 130.7, 131.4, 137.3, 143.5 (3 × C); HRMS [ES^+^, (M + H)^+^]: for C_31_H_31_O_3_S obsd 483.1939, calcd 483.1916.

Compound **15**: Compound **14** (0.5 g, 1.04 mmol) was converted to 15 (0.45 g, 84%) following the procedure described for the preparation of **9**. Eluent: EtOAc: PE (2:3); white solid; ${\left[\alpha \right]}_{D}^{27}$ = (+) 26.9 (*c* = 1.06, CHCl_3_); ^1^H-NMR (400 MHz, CDCl_3_): δ = 2.42 (s, 3H), 2.75 (dd, *J* = 8.0, 10.8 Hz, 1H), 3.32 (s, 1H), 3.55–3.64 (m, 2H), 4.02–4.03 (m, 2H), 4.40–4.42 (m, 1H), 4.72 (s, 1H), 7.25–7.39 (m, 18H), 7.62 (d, *J* = 8.0 Hz, 2H); ^13^C-NMR (100 MHz, CDCl_3_): δ = 21.9, 64.4 (CH_2_), 72.6, 73.2, 75.5 (CH_2_), 77.9, 87.7, 127.5, 128.2, 128.5, 128.8, 130.3, 135.1, 136.5, 143.4 (3 × C), 145.4; HRMS [ES^+^, (M + Na)^+^]: for C_31_H_30_O_5_NaS obsd 537.1689, calcd 537.1712.

Compound **16**: Compound **15** (0.5 g, 0.97 mmol) was converted to **16** (0.43 g, 89%) following the procedure described for the preparation of **10**. Eluent: EtOAc: PE (1:4); yellowish gum; ${\left[\alpha \right]}_{D}^{27}$ = (+) 23.5 (*c* = 1.06, CHCl_3_); ^1^H-NMR (400 MHz, CDCl_3_): δ = 2.41 (s, 3H), 3.14–3.18 (m, 1H), 3.41 (d, *J* = 10.4 Hz, 1H), 4.77–4.96 (m, 2H), 5.28 (s, 1H), 7.0 (s, 1H), 7.18–7.38 (m, 18H), 7.61 (d, *J* = 8.0 Hz, 2H); ^13^C-NMR (100 MHz, CDCl_3_): δ = 21.6, 65.2 (CH_2_), 74.6 (CH_2_), 84.1, 86.6, 127.0, 127.7, 127.8, 128.7, 129.9, 136.5, 140.8, 141.9, 143.8 (3 × C), 144.7; HRMS [ES^+^, (M + Na)^+^]: for C_31_H_28_O_4_NaS obsd 519.1578, calcd 519.1606.

Compounds **17S_S_** and **17R_S_**: A solution of NaIO_4_ (2.27 g, 10.7 mmol) in water (3 mL) was added to a well-stirred solution of **8** (2.0 g, 8.92 mmol) in MeOH (25 mL), and the mixture was stirred at room temperature. After 5 h, volatile matters were evaporated to dryness under reduced pressure, and the residue was partitioned between satd. aqueous solution of NaHCO_3_ and EtOAc (3 × 10 mL). The combined organic layer was dried over anhyd Na_2_SO_4_ and filtered, and the filtrate was concentrated under reduced pressure to get a residue. The residue was purified over silica gel to afford **17S_S_** and **17R_S_**. Compound **17S_S_**: Yield (1.13 g, 53%); Eluent: EtOAc: PE (3:2); white solid; Mp = 127 °C; ${\left[\alpha \right]}_{D}^{20.5}$ = (−) 80.7 (*c* = 0.93, CHCl_3_); ); ^1^H-NMR (200 MHz, CDCl_3_): δ = 0.90 (d, *J* = 17.2 Hz, 3H), 2.41 (s, 3H), 2.84 (t, *J* = 7.0 Hz, 1H), 3.71 (dd, *J* = 4.4, 11.2 Hz, 1H), 3.97 (dd, *J* = 4.8, 9.6 Hz, 1H), 4.47–4.67 (m, 2H), 7.33 (d, *J* = 8.0 Hz, 2H), 7.56 (d, *J* = 8.0 Hz, 2H); ^13^C-NMR (50 MHz, CDCl_3_): δ = 21.4, 21.6, 72.7, 73.1, 73.4, 73.8 (CH_2_), 124.5, 130.1, 138.5, 141.7; HRMS [ES^+^, (M + Na)^+^]: for C_12_H_16_O_3_NaS obsd 263.0688, calcd 263.0718. Compound **17R**_S_*:* Yield (0.96 g, 45%); Eluent: EtOAc: PE (4:1); white solid; Mp = 98 °C; ${\left[\alpha \right]}_{D}^{23.5}$ = (−) 23.1 (*c* = 1.01, CHCl_3_); ^1^H-NMR (400 MHz, CDCl_3_): δ = 0.78 (d, *J* = 6.0 Hz, 3H), 2.42 (s, 3H), 2.82–2.85 (m, 1H), 3.83 (dd, *J* = 2.4, 9.6 Hz, 1H), 3.94 (dd, *J* = 4.4, 10.8 Hz, 1H), 4.16–4.19 (m, 1H), 4.79 (s, 1H), 5.08 (s, 1H), 7.35 (d, *J* = 8.0 Hz, 2H), 7.63 (d, *J* = 8.0 Hz, 2H); ^13^C-NMR (100 MHz, CDCl_3_): δ = 19.8, 21.7, 73.1, 73.6, 74.3 (CH_2_), 74.5, 125.7, 130.5, 138.4, 143.1; HRMS [ES^+^, (M + Na)^+^]: for C_12_H_16_O_3_NaS obsd 263.0699, calcd 263.0718.

Compound **18S_S_**: Compound **17S_S_** (0.5 g, 2.08 mmol) was converted to **18S_S_** (0.57 g, 86%) following the procedure described for the preparation of **10**. Eluent: EtOAc: PE (1:1); colorless gum; ${\left[\alpha \right]}_{D}^{20.5}$ = (+) 130.7 (*c* = 1.01, CHCl_3_); ^1^H-NMR (400 MHz, CDCl_3_): δ = 0.96 (d, *J* = 6.0 Hz, 3H), 2.43 (s, 3H), 3.00 (t, *J* = 7.2 Hz, 1H), 3.20 (s, 3H), 3.96 (dd, *J* = 6.0, 10.0 Hz, 1H), 4.12 (dd, *J* = 5.6, 10.0 Hz, 1H), 4.60–4.65 (m, 1H), 5.28–5.32 (m, 1H), 7.37 (d, *J* = 8.0 Hz, 2H), 7.55 (d, *J* = 4.8 Hz, 2H); ^13^C-NMR (100 MHz, CDCl_3_): δ = 21.2, 21.4, 38.7, 70.0, 70.2 (CH_2_), 72.1, 77.3, 124.2, 130.1, 138.2, 141.9; HRMS [ES^+^, (M + Na)^+^]: for C_13_H_18_O_5_NaS_2_ obsd 341.0480, calcd 341.0493.

Compound **18R_S_**: Compound **17R_S_** (1.0 g, 4.16 mmol) was converted to **18R_S_** (1.17 g, 89%) following the procedure described for the preparation of **10**. Eluent: EtOAc: PE (3:2); colorless gum; ${\left[\alpha \right]}_{D}^{23.5}$ = (+) 188.7 (*c* = 1.07, CHCl_3_); ^1^H-NMR (400 MHz, CDCl_3_): δ = 0.48 (d, *J* = 6.4 Hz, 3H), 2.43 (s, 3H), 3.20–3.23 (m, 1H), 3.27 (s, 3H), 3.99–4.04 (m, 2H), 4.22 (d, *J* = 11.2 Hz, 1H), 4.45 (bs, 1H), 7.36 (d, *J* = 8.0 Hz, 2H), 7.62 (d, *J* = 8.0 Hz, 2H); ^13^C-NMR (100 MHz, CDCl_3_): δ = 19.9, 21.8, 38.6, 72.4 (CH_2_), 72.9, 75.2, 81.8, 125.8, 130.7, 138.4, 143.9; HRMS [ES^+^, (M + Na)^+^]: for C_13_H_18_O_5_NaS_2_ obsd 341.0487, calcd 341.0493.

Compound **19R**_S_: Compound **18S_S_** (0.5 g, 1.58 mmol) was treated with DBU (0.5 mL, 3.16 mmol) in DCM (5 mL) at ambient temperature for 3 h. Solvent was evaporated under reduced pressure, and the resulting residue was purified over silica gel to afford **18R_S_** (0.34 g, 95%). Eluent: EtOAc: PE (2:3); colorless gum; ${\left[\alpha \right]}_{D}^{20.5}$ = (−) 137.5 (*c* = 0.98, CHCl_3_); ); ^1^H-NMR (400 MHz, CDCl_3_): δ = 1.21 (d, *J* = 6.4 Hz, 3H), 2.41 (s, 3H), 4.54–4.55 (m, 1H), 4.69 (d, *J* = 14.4 Hz, 1H), 4.82 (dd, *J* = 5.6, 14.0 Hz, 1H), 6.59 (s, 1H), 7.32 (d, *J* = 8.0 Hz, 2H), 7.54 (d, *J* = 7.6 Hz, 2H); ^13^C-NMR (100 MHz, CDCl_3_): δ = 21.2, 21.7, 74.7 (CH_2_), 80.6, 125.7, 130.5, 130.5, 138.6, 142.9, 148.8; HRMS [ES^+^, (M + H)^+^]: for C_12_H_15_O_2_S obsd 223.0784, calcd 223.0793.

Compound **19S**_S_: Compound **18R_S_** (0.5 g, 1.58 mmol) was converted to **19S_S_** (0.34 g, 95%) following the procedure described for the preparation of **19R_S_**. Eluent: EtOAc: PE (2:3); Yellowish gum; ${\left[\alpha \right]}_{D}^{23.5}$ = (+) 55.7 (c = 1.01, CHCl_3_); ^1^H-NMR (400 MHz, CDCl_3_): δ = 1.25 (d, *J* = 6.0 Hz, 3H), 2.37 (s 3H), 4.58 (d, *J* = 12.8 Hz, 1H), 4.69–4.77 (m, 2H), 6.52 (s, 1H), 7.28 (d, *J* = 8.0 Hz, 2H), 7.47 (d, *J* = 8.0 Hz, 2H); ^13^C-NMR (100 MHz, CDCl_3_): δ = 21.4, 21.7, 73.6 (CH_2_), 80.4, 124.7, 130.0, 134.4, 138.4, 141.8, 146.9; HRMS [ES^+^, (M + H)^+^]: for C_12_H_15_O_2_S obsd 223.0806, calcd 223.0793.

Compound **20**: Compound **14** (1.0 g, 2.07 mmol) was converted to the diastereomeric mixture of sulfoxides **20** (0.95 g, 92%) following the procedure described for the preparation of **17S_S_** and **17R_S_**. Colorless gum; ^1^H-NMR (400 MHz, CDCl_3_): δ = 2.33, 2.37, 2.81–2.85, 3.28–3.31, 3.42–3.45, 3.96–4.00, 4.08–4.11, 7.09–7.45; HRMS [ES^+^, (M + Na)^+^]: for C_31_H_30_O_4_NaS obsd 521.1756, calcd 521.1762.

Compound **21**: A solution of mesylchloride (0.25 mL, 3.0 mmol) in anhyd pyridine (3 mL) was dropwise added to a stirred solution of **20** (0.5 g, 1.00 mmol) in anhyd pyridine (5 mL) at 0 °C. After 0.5 h, the solution was stored at +4 °C. After 24 h (tlc), the reaction mixture was poured into ice-cold water, and the compound was extracted with EtOAc. The organic layer was separated, dried over anhyd Na_2_SO_4_, and filtered, and the filtrate was evaporated to dryness. Residual pyridine was co-evaporated with toluene. The residue was treated with DBU (0.4 mL, 2.5 mmol) in DCM (8 mL) at ambient temperature for 2 h. Solvent was evaporated under reduced pressure, and the resulting residue was purified over silica gel to afford the diastereomeric mixture of vinyl sulfoxides **21** (0.44 g, 92%). Colorless gum; ^1^H-NMR (400 MHz, CDCl_3_): δ = 2.25, 2.44, 3.03–3.07, 3.17–3.21, 3.33–3.92, 4.50, 4.74–5.04, 6.50, 6.81, 7.24–7.47; HRMS [ES^+^, (M + Na)^+^]: for C_31_H_28_O_3_NaS obsd 503.1664, calcd 503.1657.

Compound **22**: Sodium methoxide (0.02 g, 0.36 mmol) was added to an anhyd methanolic solution (5 mL) of vinyl sulfone **10** (0.043 g, 0.18 mmol), and the mixture was stirred at room temperature. After 4 h (tlc), volatile matters were removed under reduced pressure. The solid residue was stirred in a mixture of EtOAc and satd. aqueous NaHCO_3_ solution for 1 h. Organic layers were pooled together, dried over anhyd Na_2_SO_4_, and filtered, and the filtrate was evaporated. The residue thus obtained was purified over silica gel to afford **22** (0.04 g, 89%). Eluent: EtOAc: PE (1:4); White solid; Mp = 89 °C; ${\left[\alpha \right]}_{D}^{23.5}$ = (+) 14.1 (c = 0.42, CHCl_3_); ^1^H-NMR (400 MHz, CDCl_3_): δ = 1.20 (d, *J* = 6.0 Hz, 3H), 2.50 (s, 3H), 3.20 (s, 3H), 3.21–3.24 (m, 1H), 3.72 (dd, *J* = 4.4, 10.4 Hz, 1H), 4.01 (d, *J* = 10.4 Hz, 1H), 4.23–4.32 (m, 2H), 7.42 (d, *J* = 8.4 Hz, 2H), 7.83 (d, *J* = 8.4 Hz, 2H); ^13^C-NMR (100 MHz, CDCl_3_): δ = 20.4, 21.6, 56.9, 72.5 (CH_2_), 75.6, 75.6, 82.9, 128.5, 130.1, 135.2, 145.3; HRMS [ES^+^, (M + H)^+^]: for C_13_H_19_O_4_S obsd 271.1015, calcd 271.1004.

Compound **23**: A mixture of dimethylmalonate (0.28 mL, 2.52 mmol) and KO^t^Bu (0.23 g, 2.1 mmol) in THF (5 mL) was stirred at room temperature in a N_2_ atmosphere. After 0.5 h, a solution of **10** (0.2 g, 0.84 mmol) in THF (4 mL) was added to the reaction mixture. After stirring for 6 h (tlc), the mixture was evaporated under reduced pressure. The residue was partitioned between a mixture of EtOAc and satd. aqueous NH_4_Cl. The organic part was separated, dried over anhyd Na_2_SO_4_, and filtered, and the filtrate was evaporated to dryness. The residue thus obtained was purified over silica gel coloumn to afford **23** (0.23 g, 78%). Eluent: EtOAc: PE (1:4); Colorless gum; ${\left[\alpha \right]}_{D}^{23.5}$ = (+) 45.6 (*c* = 1.07, CHCl_3_); ^1^H-NMR (200 MHz, CDCl_3_): δ =1.11 (d, *J* = 6.0 Hz, 3H), 2.47 (s, 3H), 3.33–3.46 (m, 3H), 3.70 (d, *J* = 14.0 Hz, 6H), 3.86–3.89 (m, 2H), 4.16–4.22 (m, 1H), 7.40 (d, *J* = 8.0 Hz, 2H), 7.78 (d, *J* = 8.2 Hz, 2H); ^13^C-NMR (50 MHz, CDCl_3_): δ = 20.2, 21.8, 42.0, 52.9, 53.9, 70.7 (CH_2_), 71.5, 76.3, 128.9, 130.3, 135.1, 145.5, 168.2; HRMS [ES^+^, (M + H)^+^]: for C_17_H_23_O_6_S obsd 355.1249, calcd 355.1215.

Compound **24**: A well-stirred solution of thymine (0.16 g, 1.26 mmol) and TMG (0.11 mL, 0.9 mmol) in DMF (10 mL) was added to **10** (0.043 g, 0.18 mmol), and the mixture was stirred at ambient temperature in a nitrogen atmosphere. After 5 h, the reaction mixture was diluted with EtOAc (20 mL), and the precipitated solid was filtered off. The filtrate was washed with an aqueous satd. aqueous solution of NaHCO_3_, and the aqueous part was extracted with EtOAc (3 × 10 mL). The combined organic layer was dried over anhyd Na_2_SO_4_ and filtered, and the filtrate was concentrated under reduced pressure. The residue was purified over silica gel to get **24** (0.05 g, 88%). Eluent: EtOAc: PE (1:3); White solid; Mp = 145 °C; ${\left[\alpha \right]}_{D}^{23.5}$ = (−) 69.5 (*c* = 0.99, CHCl_3_); ^1^H-NMR (400 MHz, CDCl_3_): δ = 1.43 (d, *J* = 6.4 Hz, 3H), 1.86 (s, 3H), 2.43 (s, 3H), 3.55 (dd, *J* = 4.8, 7.6 Hz, 1H), 3.94–4.03 (m, 2H), 4.35–4.41 (m, 1H), 5.40–5.43 (m, 1H), 7.06 (s, 1H), 7.37 (d, *J* = 8.4 Hz, 2H), 7.82 (d, *J* = 8.0 Hz, 2H), 9.12 (s, 1H); ^13^C-NMR (100 MHz, CDCl_3_): δ = 12.5, 20.3, 21.7, 58.8, 71.2 (CH_2_), 74.1, 76.1, 112.4, 128.7, 130.1, 134.5, 136.9, 145.8, 149.9, 163.3; HRMS [ES^+^, (M + H)^+^]: for C_17_H_21_N_2_O_5_S obsd 365.1156, calcd 365.1171.

Compound **25**: Benzylamine (0.2 mL, 1.8 mmol) was added to an anhyd methanolic solution (5 mL) of **10** (0.043 g, 0.18 mmol), and the mixture was stirred at room temperature. After 4 h (tlc), volatile matters were removed under reduced pressure. The residue was partitioned between EtOAc and satd. aqueous NH_4_Cl solution. Then, the organic part was dried over anhyd Na_2_SO_4_ and filtered, and the filtrate was evaporated to dryness. The residue thus obtained was purified over silica gel to afford **25** (0.05 g, 88%). Eluent: EtOAc: PE (1:3); Brown solid; Mp = 139 °C; ${\left[\alpha \right]}_{D}^{23.5}$ = (−) 23.1 (*c* = 0.48, CHCl_3_); ^1^H-NMR (400 MHz, CDCl_3_): δ = 1.22 (d, *J* = 6.4 Hz, 3H), 2.46 (s, 3H), 3.13–3.16 (m, 1H), 3.72 (q, *J* = 3.2 Hz, 2H), 3.76–3.85 (m, 3H), 4.22–4.25 (m, 1H), 7.18 (d, *J* = 8.0 Hz, 2H), 7.23–7.35 (m, 6H), 7.71 (d, *J* = 8.4 Hz, 2H); ^13^C-NMR (100 MHz, CDCl_3_): δ = 20.9, 21.6, 51.4 (CH_2_), 60.8, 72.6 (CH_2_), 75.3, 75.8, 127.1, 128.0, 128.4, 128.5, 130.0, 135.0, 139.1, 145.1; HRMS [ES^+^, (M + H)^+^]: for C_19_H_24_NO_3_S obsd 346.1447, calcd 346.1477.

Compound **26**: Cyclohexylamine (1.9 mL, 16.80 mmol) was reacted with 10 (0.2 g, 0.84 mmol) following the procedure described for **25** to afford **26** (0.24 g, 89%). Eluent: EtOAc: PE (1:3); Brownish gum; ${\left[\alpha \right]}_{D}^{23.5}$ = (−) 37.6 (*c* = 1.09, CHCl_3_); ^1^H-NMR (400 MHz, CDCl_3_): δ = 1.02–1.16 (m, 3H), 1.21–1.25 (m, 4H), 1.54–1.60 (m, 7H), 2.12–2.18 (m, 1H), 2.46 (s, 3H), 3.08 (dd, *J* = 3.2, 7.2 Hz, 1H), 3.72–3.82 (m, 3H), 4.25–4.28 (m, 1H), 7.38 (d, *J* = 8.0 Hz, 2H), 7.80 (d, *J* = 8.4 Hz, 2H); ^13^C-NMR (100 MHz, CDCl_3_): δ = 21.2, 21.6, 24.6 (CH_2_), 24.8 (CH_2_), 25.8 (CH_2_), 33.3 (2 × CH_2_), 54.2, 58.4, 73.7 (CH_2_), 75.0, 75.9, 128.5, 130.0, 135.3, 145.1; HRMS [ES^+^, (M + H)^+^]: for C_18_H_28_NO_3_S obsd 338.1771, calcd 338.1790.

Compound **27**: Morpholine (1.4 mL, 16.80 mmol) was reacted with **10** (0.2 g, 0.84 mmol) following the procedure described for **25** to afford **27** (0.22 g, 86%). Eluent: EtOAc: PE (1:4); Brownish solid; Mp = 121 °C; ${\left[\alpha \right]}_{D}^{23.5}$ = (−) 33.6 (*c* = 1.05, CHCl_3_); ^1^H-NMR (400 MHz, CDCl_3_): δ = 1.00 (d, *J* = 6.0 Hz, 3H), 2.08–2.11 (m, 2H), 2.41 (s, 3H), 3.25 (d, *J* = 7.2 Hz, 1H), 3.55–3.59 (m, 4H), 3.66–3.71 (m, 1H), 3.82–3.83 (m, 1H), 4.00 (d, *J* = 10.4 Hz, 1H), 4.11 (t, *J* = 6.8 Hz, 1H), 7.34 (d, *J* = 7.6 Hz, 2H), 7.74 (d, *J* = 8.0 Hz, 2H); ^13^C-NMR (100 MHz, CDCl_3_): δ = 19.2, 21.8, 49.5 (CH_2_), 66.8 (CH_2_), 68.1, 68.8, 71.0 (CH_2_), 76.2, 128.8, 130.2, 135.3, 145.5; HRMS [ES^+^, (M + H)^+^]: for C_16_H_24_NO_4_S obsd 326.1463, calcd 326.1426.

Compound **28**: Compound **16** (0.043 g, 0.09 mmol) was converted to **28** (0.04 g, 89%) following the procedure described for the preparation of **22**. Eluent: EtOAc: PE (1:4); White sold; Mp = 111 °C; ${\left[\alpha \right]}_{D}^{26}$ = (+) 56.3 (*c* = 0.86, CHCl_3_); ^1^H-NMR (400 MHz, CDCl_3_): δ = 2.40 (s, 3H), 2.71 (dd, *J* = 4.4, 10.0 Hz, 1H), 3.24 (d, *J* = 3.6 Hz, 1H), 3.27 (s, 3H), 3.84 (d, *J* = 5.2 Hz, 1H), 3.93 (dd, *J* = 4.8, 9.6 Hz, 1H), 4.10 (d, *J* = 10.0 Hz, 1H), 4.33–4.36 (m. 1H), 4.44–4.45 (m, 1H), 7.21–7.28 (m, 12H), 7.37 (d, *J* = 7.2 Hz, 6H), 7.67 (d, *J* = 8.4 Hz, 2H); ^13^C-NMR (100 MHz, CDCl_3_): δ = 21.6, 56.9, 63.5 (CH_2_), 69.6, 73.3 (CH_2_), 78.8, 82.1, 86.4, 126.9, 127.7, 128.3, 128.6, 130.0, 135.0, 143.6 (3 × C), 144.9; HRMS [ES^+^, (M + Na)^+^]: for C_32_H_32_O_5_NaS obsd 551.1858, calcd 551.1868.

Compound **29**: A mixture of nitromethane (0.03 mL, 0.27 mmol) and KO^t^Bu (0.02 g, 0.22 mmol) in THF (5 mL) was reacted with Compound **16** (0.043 g, 0.09 mmol) in THF (4 mL) following the procedure described for **23** to afford **29** (0.047 g, 94%). Eluent: EtOAc: PE (1:4); Colorless gum; ${\left[\alpha \right]}_{D}^{26}$ = (+) 89.2 (*c* = 0.95, CHCl_3_); ^1^H-NMR (400 MHz, CDCl_3_): δ = 2.45 (s, 3H), 2.73 (dd, *J* = 3.2, 10.8 Hz, 1H), 3.47–3.53 (m, 2H), 3.57–3.60 (m, 1H) 4.00 (dd, *J* = 3.6, 9.2 Hz, 1H), 4.13–4.17 (m, 1H), 4.39–4.42 (m, 1H), 4.56 (d, *J* = 7.2 Hz, 2H), 7.26–7.38 (m, 18H), 7.64 (d, *J* = 8.4 Hz, 2H); ^13^C-NMR (100 MHz, CDCl_3_): δ = 21.7, 40.3, 63.6 (CH_2_), 66.1, 71.3 (CH_2_), 76.0 (CH_2_), 79.0, 87.2, 127.2, 127.9, 128.4, 128.5, 130.2, 134.1, 143.2 (3 × C), 145.5; HRMS [ES^+^, (M + Na)^+^]: for C_32_H_31_NO_6_NaS obsd 580.1763, calcd 580.1770.

Compound **30**: A mixture of dimethylmalonate (0.13 mL, 1.21 mmol) and KO^t^Bu (0.11 g, 1.0 mmol) in THF (5 mL) was reacted with Compound **16** (0.2 g, 0.4 mmol) in THF (4 mL) following the procedure described for **23** to afford **30** (0.24 g, 96%). Eluent: EtOAc: PE (1:4); Colorless gum; ${\left[\alpha \right]}_{D}^{26}$ = (+) 73.6 (*c* = 0.59, CHCl_3_); ^1^H-NMR (400 MHz, CDCl_3_): δ = 2.40 (s, 3H), 2.74 (dd, *J* = 4.4, 10.4 Hz, 1H), 3.26 (dd, *J* = 2.4, 10.4 Hz, 1H), 3.39–3.43 (m, 1H), 3.61 (s, 3H), 3.69 (s, 3H), 3.83–3.91 (m, 1H), 4.06–4.10 (m, 1H), 4.33–4.36 (m, 1H), 7.20–7.30 (m, 12H), 7.37 (d, *J* = 7.2 Hz, 6H), 7.63 (d, *J* = 8.0 Hz, 2H); ^13^C-NMR (100 MHz, CDCl_3_): δ = 21.7, 41.1, 52.7, 52.8, 53.2, 63.8 (CH_2_), 66.2, 71.6 (CH_2_), 79.3, 86.9, 127.0, 127.7, 128.6, 128.7, 129.9, 134.7, 143.4 (3 × C), 144.9, 167.9, 168.1; HRMS [ES^+^, (M + Na)^+^]: for C_36_H_36_O_8_NaS obsd 651.2018, calcd 651.2029.

Compound **31**: Compound **16** (0.043 g, 0.09 mmol) was converted to **31** (0.046 g, 88%) following the procedure described under the preparation of **24**. Eluent: EtOAc: PE (1:3); White solid; Mp = 110 °C; ${\left[\alpha \right]}_{D}^{26}$ = (+) 129.6 (*c* = 0.91, CHCl_3_); ^1^H-NMR (400 MHz, CDCl_3_): δ = 1.43 (s, 3H), 2.42 (s, 3H), 3.07 (dd, *J* = 3.2, 10.8 Hz, 1H), 3.66 (dd, *J* = 1.6, 10.8 Hz, 1H), 4.05–4.21 (m, 3H), 4.41–4.46 (m, 1H), 5.73–5.77 (m, 1H), 7.27–7.41 (m, 18H), 7.75 (d, *J* = 8.0 Hz, 2H), 9.31 (s, 1H); ^13^C-NMR (100 MHz, CDCl_3_): δ = 12.3, 21.9, 55.9, 63.5 (CH_2_), 68.5, 72.4 (CH_2_), 79.4, 87.6, 112.7, 127.6, 128.1, 128.8, 130.3, 134.5, 136.8, 143.2 (3 × C), 145.8, 150.4, 163.6; HRMS [ES^+^, (M + Na)^+^]: for C_36_H_34_N_2_O_6_NaS obsd 645.2030, calcd 645.2029.

Compound **32**: Compound **16** (0.043 g, 0.09 mmol) was converted to **32** (0.046 g, 88%) following the procedure described for **25**. Eluent: EtOAc: PE (1:3); Colorless gum; ${\left[\alpha \right]}_{D}^{26}$ = (+) 91.4 (*c* = 0.69, CHCl_3_); ^1^H-NMR (400 MHz, CDCl_3_): δ = 2.42 (s, 3H), 2.70 (dd, *J* = 3.2, 10.4 Hz, 1H), 3.44–3.46 (m, 1H), 3.62 (d, *J* = 13.6 Hz, 1H), 3.70–3.73 (m, 2H), 3.81–3.82 (m, 1H), 3.95–4.03 (m, 2H), 4.34–4.35 (m, 1H), 7.19–7.29 (m, 16H), 7.37 (d, *J* = 6.4 Hz, 6H), 7.59 (d, *J* = 8.0 Hz, 2H); ^13^C-NMR (100 MHz, CDCl_3_): δ = 21.7, 51.2 (CH_2_), 60.3, 63.8 (CH_2_), 69.6, 73.8 (CH_2_), 78.3, 86.9, 127.0, 127.0, 127.8, 128.0, 128.3, 128.3, 128.6, 130.0, 134.9, 139.3, 143.4 (3 × C), 144.8; HRMS [ES^+^, (M + H)^+^]: for C_38_H_38_NO_4_S obsd 604.2511, calcd 604.2522.

Compound **33**: Cyclohexylamine (0.92 mL, 8.06 mmol) was reacted with **16** (0.2 g, 0.40 mmol) following the procedure described for **25** to afford **33** (0.21 g, 89%). Eluent: EtOAc: PE (1:3); Colorless gum; ${\left[\alpha \right]}_{D}^{26}$ = (+) 103.6 (*c* = 1.03, CHCl_3_); ^1^H-NMR (400 MHz, CDCl_3_): δ = 0.90–1.37 (m, 7H), 1.52–1.66 (m, 7H), 2.22–2.27 (m, 1H), 2.43 (s, 3H), 2.73 (dd, *J* = 3.2, 10.4 Hz, 1H), 3.45 (dd, *J* = 2.8, 10.4 Hz, 1H), 3.61–3.63 (m, 1H), 3.88–4.02 (m, 3H), 4.34–4.37 (m, 1H), 7.25–7.42 (m, 18H), 7.66 (d, *J* = 8.4 Hz, 2H); ^13^C-NMR (100 MHz, CDCl_3_): δ = 21.6, 24.6 (CH_2_), 24.8 (CH_2_), 26.0 (CH_2_), 32.9 (CH_2_), 33.3 (CH_2_), 53.9, 57.7, 63.8 (CH_2_), 69.8, 74.4 (CH_2_), 78.1, 86.9, 126.9, 127.0, 127.7, 127.8, 128.3, 128.6, 128.7, 129.9, 135.1, 143.5 (3 × C), 144.8; HRMS [ES^+^, (M + H)^+^]: for C_37_H_42_NO_4_S obsd 596.2827, calcd 596.2829.

Compound **34**: Morpholine (0.70 mL, 8.06 mmol) was reacted with **16** (0.2 g, 0.40 mmol) following the procedure described for **25** to afford **34** (0.0.20 g, 86%). Eluent: EtOAc: PE (1:4); Brownish gum; ${\left[\alpha \right]}_{D}^{26}$ = (+) 96.2 (*c* = 0.87, CHCl_3_); ^1^H-NMR (400 MHz, CDCl_3_): δ = 2.40 (s, 3H), 2.45–2.49 (m, 3H), 2.84 (dd, *J* = 5.2, 10.4 Hz, 1H), 3.14 (d, *J* = 9.6 Hz, 1H), 3.58 (s, 3H), 3.77–3.90 (m, 3H), 4.14–4.16 (m, 1H), 4.28–4.30 (m, 1H), 7.19–7.37 (m, 18H), 7.65 (d, *J* = 8.0 Hz, 2H); ^13^C-NMR (100 MHz, CDCl_3_): δ = 21.9, 50.0 (CH_2_), 63.7 (CH_2_), 64.6, 66.9 (CH_2_), 67.6, 70.5 (CH_2_), 79.3, 87.2, 127.3, 127.9, 128.2, 128.6, 128.9, 130.1, 130.5, 131.3, 135.3, 143.6 (3 × C), 145.2; HRMS [ES^+^, (M + H)^+^]: for C_35_H_38_NO_5_S obsd 584.2498, calcd 584.2471.

Compound **35S**_S_: Compound **19R_S_** (0.07 g, 0.31 mmol) was converted to **35S_S_** (0.07 g, 93%) following the procedure described for the preparation of **22**. Eluent: EtOAc: PE (1:2); Colorless gum; ${\left[\alpha \right]}_{D}^{25.3}$ = (−) 35.6 (*c* = 1.01, CHCl_3_); ^1^H-NMR (400 MHz, CDCl_3_): δ = 0.89 (d, *J* = 6.0 Hz, 3H), 2.42 (s, 3H), 2.78 (d, *J* = 6.0 Hz, 1H), 3.27 (s, 3H), 3.59–3.63 (m, 1H), 3.96 (d, *J* = 10.4 Hz, 1H), 4.18–4.23 (m, 2H), 7.34 (d, *J* = 7.6 Hz, 2H), 7.50 (d, *J* = 8.0 Hz, 2H); ^13^C-NMR (100 MHz, CDCl_3_): δ = 20.8, 21.4. 57.0, 71.4 (CH_2_), 72.1, 73.1, 83.5, 124.2, 130.0, 137.8, 141.9; HRMS [ES^+^, (M + H)^+^]: for C_13_H_19_O_3_S obsd 255.1071, calcd 255.1055.

Compound **35R_S_**: Compound **19S_S_** (0.07 g, 0.31 mmol) was converted to **35R_S_** (0.069 g, 91%) following the procedure described for the preparation of **22**. Eluent: EtOAc: PE (1:2); Colorless gum; ${\left[\alpha \right]}_{D}^{25.3}$ = (+) 126.8 (c = 0.99, CHCl_3_); ^1^H-NMR (400 MHz, CDCl_3_): δ = 1.22 (d, *J* = 6.4 Hz, 3H), 2.41 (s, 3H), 2.78 (d, *J* = 7.6 Hz, 1H), 2.97 (s, 3H), 3.65–3.69 (m, 1H), 3.96–4.02 (m, 2H), 4.23 (s, 1H), 7.34 (d, *J* = 7.6 Hz, 2H), 7.53 (d, *J* = 8.0 Hz, 2H); ^13^C-NMR (100 MHz, CDCl_3_): δ = 19.7, 21.4, 56.5, 72.8 (CH_2_), 75.0, 75.6, 80.8, 124.5, 130.0, 138.5, 142.1; HRMS [ES^+^, (M + H)^+^]: for C_13_H_19_O_3_S obsd 255.1069, calcd 255.1055.

Compound **22** from **35R_S_** or **35S_S_**: Compound **35R_S_** (0.1 g, 0.39 mmol) was converted to **22** (0.09 g, 85%) following the procedure described for the preparation of **9**. Compound **35S_S_** was converted to **22** in a similar fashion.

Compound **36S_S_**: Dimethylmalonate (0.5 mL, 4.05 mmol) was added to a well stirred solution of NaH (0.05 g, 3.37 mmol) in DMF (5 mL), and the mixture was stirred for 0.5 h at ambient temperature in an inert atmosphere. Vinyl sulfoxide **19R_S_** (0.3 g, 1.35 mmol) was added, and the whole mixture was stirred at 60 °C. After 6 h (tlc), the reaction mixture was partitioned between aqueous satd. solution of NaHCO_3_ and EtOAc (3 × 10 mL). The combined organic layer was dried over anhyd Na_2_SO_4_ and filtered, and the filtrate was concentrated under reduced pressure to get a residue. The residue was purified over silica gel to get **36S_S_** (0.45 g, 95%). Eluent: EtOAc: PE (1:3); Colorless gum; ${\left[\alpha \right]}_{D}^{25.3}$ = (−) 69.3 (*c* = 0.65, CHCl_3_); ^1^H-NMR (400 MHz, CDCl_3_): δ = 1.24 (d, *J* = 6.0 Hz, 3H), 2.50–2.60 (m, 2H), 3.61 (s, 3H), 3.68 (s, 3H), 3.76–3.87 (m, 2H), 3.92–4.02 (m, 1H), 7.37 (d, *J* = 8.0 Hz, 2H), 7.52 (d, *J* = 8.0 Hz, 2H); ^13^C-NMR (100 MHz, CDCl_3_): δ = 19.7, 21.5, 39.7, 52.8, 54.0, 67.1 (CH_2_), 72.0, 76.3, 124.6, 130.0, 138.2, 141.9, 167.9, 168.1; HRMS [ES^+^, (M + Na)^+^]: for C_17_H_22_O_6_NaS obsd 377.1058, calcd 377.1035.

Compound **36R_S_**: A mixture of dimethylmalonate (0.5 mL, 4.05 mmol) and NaH (0.05 g, 3.37 mmol) in DMF (5 mL) was reacted with Compound **19S_S_** (0.3 g, 1.35 mmol) in DMF (4 mL) following the procedure described for **36S_S_** to afford Compound **36R_S_** (0.45 g, 95%). Eluent: EtOAc: PE (1:3); Colorless gum compound; ${\left[\alpha \right]}_{D}^{25.3}$ = (+) 112.5 (c = 0.98, CHCl_3_); ^1^H-NMR (400 MHz, CDCl_3_): δ = 1.15 (d, *J* = 6.0 Hz, 3H), 2.41 (s, 3H), 2.94–2.97 (m, 1H), 3.02 (d, *J* = 6.4 Hz, 1H), 3.27–3.32 (m, 1H), 3.62 (s, 3H), 3.68 (s, 3H), 3.76–3.82 (m, 2H), 3.80 (dd, *J* = 2.8, 9.6 Hz, 1H), 4.00 (q, *J* = 6.4 Hz, 1H), 7.33 (d, *J* = 8.0 Hz, 2H), 7.52 (d, *J* = 8.0 Hz, 2H); ^13^C-NMR (100 MHz, CDCl_3_): δ = 19.5, 21.4, 39.4, 52.6, 53.8, 70.6 (CH_2_), 71.6, 75.3, 124.5, 130.1, 138.2, 142.1, 168.1, 168.2; HRMS [ES^+^, (M + Na)^+^]: for C_17_H_22_O_6_NaS obsd 377.1064, calcd 377.1035.

Compound **26** from **36R_S_** or **36S_S_**: Compound **36R_S_** (0.06 g, 0.16 mmol) was converted to **26** (0.048 g, 77%) following the procedure described for the preparation of **9**. Compound **36S_S_** was converted to **26** in a similar fashion. 

Compound **37S_S_**: A mixture of vinyl sulfoxide **19R_S_** (0.3 g, 1.35 mmol) and benzylamine (2.9 mL, 27.0 mmol) was stirred at room temperature for 42 h. After completion of reaction, the mixture was partitioned between EtOAc and satd. aqueous NH_4_Cl solution. Then, the organic part was separated, dried over anhyd Na_2_SO_4_, and filtered, and the filtrate was evaporated to dryness. The residue thus obtained was purified over silica gel to afford Compound **37S_S_** (0.41 g, 93%). Eluent: EtOAc: PE (2:3); Brownish gum; ${\left[\alpha \right]}_{D}^{25.3}$ = (−) 59.6 (*c* = 0.88, CHCl_3_); ); ^1^H-NMR (400 MHz, CDCl_3_): δ = 1.70 (d, *J* = 6.4 Hz, 3H), 1.88–2.10 (m, 3H), 2.44 (s, 3H), 2.58–2.79 (m, 1H), 4.00 (d, *J* = 10.4 Hz, 2H), 4.39 (t, *J* = 8.0 Hz, 1H), 7.38–7.60 (m, 7H), 7.68 (d, *J* = 8.0 Hz, 2H); ^13^C-NMR (100 MHz, CDCl_3_): δ = 21.3, 21.4, 52.2 (CH2), 58.8, 71.6, 74.7 (CH2), 76.6, 123.7, 126.9, 128.0, 128.0, 130.2, 138.9, 139.3, 142.3; HRMS [ES^+^, (M + H)^+^]: for C_19_H_24_NO_2_S obsd 330.1554, calcd 330.1528.

Compound **37R_S_**: Benzylamine (2.9 mL, 27.0 mmol) was reacted with **19S_S_** (0.3 g, 1.35 mmol) following the procedure described for **37S_S_** to afford **37R_S_** (0.41 g, 93%). Eluent: EtOAc: PE (2:3); Brownish gum; ${\left[\alpha \right]}_{D}^{25.3}$ = (−) 103.7 (*c* = 0.76, CHCl_3_); ^1^H-NMR (400 MHz, CDCl_3_): δ = 1.19 (d, *J* = 6.0 Hz, 3H), 2.42 (s, 3H), 2.68–2.70 (m, 1H), 3.49 (t, *J* = 15.2 Hz, 2H), 3.77–3.85 (m, 3H), 4.00–4.06 (m, 1H), 7.09 (d, *J* = 6.8 Hz, 2H), 7.21–7.34 (m, 6H), 7.52 (d, *J* = 8.0 Hz, 2H); ^13^C-NMR (100 MHz, CDCl_3_): δ = 20.0, 21.4, 51.9 (CH_2_), 59.0, 73.0 (CH_2_), 74.8, 76.0, 124.4, 127.1, 128.0, 128.3, 130.1, 138.6, 139.3, 142.1; HRMS [ES^+^, (M + H)^+^]: for C_19_H_24_NO_2_S obsd 330.1563, calcd 330.1528.

Compound **25** from **37R_S_** or **37S_S_**: Compound **37R_S_** (0.1 g, 0.30 mmol) was converted to **25** (0.031 g, 30%) following the procedure described for the preparation of **9**. Compound **37S_S_** was converted to **25** in a similar fashion. The oxidation of **37R_S_**/**37S_S_** was quenched within 0.5 h to avoid over-oxidation.

Compound **38S_S_**: Cyclohexylamine (3.1 mL, 27.0 mmol) was reacted with **19R_S_** (0.3 g, 1.35 mmol) following the procedure described for **37S_S_** to afford **38S_S_** (0.39 g, 90%). Eluent: EtOAc: PE (2:3); Brownish gum; ${\left[\alpha \right]}_{D}^{25.3}$ = (−) 39.5 (*c* = 0.86, CHCl_3_); ^1^H-NMR (400 MHz, CDCl_3_): δ = 0.94–1.01 (m, 5H), 1.08–1.19 (m, 3H), 1.57–1.74 (m, 7H), 2.28 (t, *J* = 10.4 Hz, 1H), 2.41 (s, 3H), 2.61–2.63 (m, 1H), 3.62 (d, *J* = 3.6 Hz, 1H), 3.74 (d, *J* = 3.2 Hz, 2H), 4.22 (t, *J* = 6.4 Hz, 1H), 7.33 (d, *J* = 7.6 Hz, 2H), 7.52 (d, *J* = 7.6 Hz, 2H); ^13^C-NMR (100 MHz, CDCl_3_): δ = 21.4, 21.4, 24.8 (CH_2_), 25.8, 33.2 (CH_2_), 33.6 (CH_2_), 54.7, 58.5, 72.9, 72.9 (CH_2_), 74.6, 124.4, 130.0, 138.4, 141.9; HRMS [ES^+^, (M + H)^+^]: for C_18_H_28_NO_2_S obsd 322.1875, calcd 322.1841.

Compound **38R_S_**: Cyclohexylamine (3.1mL, 27.0 mmol) was reacted with **19S_S_** (0.3 g, 1.35 mmol) following the procedure described for **37S_S_** to afford **38R_S_** (0.39 g, 90%). Eluent: EtOAc: pet ether (2:3); Brownish gum; ${\left[\alpha \right]}_{D}^{25.3}$ = (−) 120.3 (*c* = 0.91, CHCl_3_); ^1^H-NMR (400 MHz, CDCl_3_): δ = 0.46–0.58 (m, 2H), 0.95–1.03 (m, 3H), 1.22–1.35 (m, 4H), 1.69–1.75 (m, 4H), 2.44 (s, 3H), 2.92–2.95 (m, 1H), 3.05 (t, *J* = 5.6 Hz, 1H), 3.30–3.36 (m, 1H), 4.08 (t, *J* = 8.0 Hz, 1H), 4.39 (t, *J* = 6.4 Hz, 1H), 7.38 (d, *J* = 8.0 Hz, 2H), 7.69 (d, *J* = 7.6 Hz, 2H); ^13^C-NMR (100 MHz, CDCl_3_): δ = 16.5, 21.4, 24.7 (CH_2_), 24.8 (CH_2_), 25.7 (CH_2_), 33.0 (CH_2_), 33.8 (CH_2_), 55.1, 57.0, 73.7 (CH_2_), 75.5, 76.3, 126.3, 130.1, 138.8, 142.9; HRMS [ES^+^, (M + H)^+^]: for C_18_H_28_NO_2_S obsd 322.1825, calcd 322.1841.

Compound **26** from **38R_S_** or **38S_S_**: Compound **38R_S_** (0.1 g, 0.31 mmol) was converted to **26** (0.029 g, 28%) following the procedure described for the preparation of **9**. Compound **38S_S_** was converted to **26** in a similar fashion. The oxidation of **38R_S_**/**38S_S_** was quenched within 0.5 h to avoid over-oxidation.

Compound **28** from **21**: Compound **21** (0.3 g, 0.62 mmol) was converted to **39** following the procedure described for the preparation of **22**. The inseparable mixture **39** was converted to **28** following the procedure described for the preparation of **9**.

Compound **30** from **21**: Compound **21** (0.3 g, 0.62 mmol) was converted to **40** following the procedure described under the preparation of **36S_S_**. The inseparable mixture **40** was converted to **30** following the procedure described for the preparation of **9**.

Compound **33** from **21**: Compound **21** (0.3 g, 0.62 mmol) was converted to **41** following the procedure described for the preparation of Compound **37S_S_**. The inseparable mixture **41** was converted to **33** following the procedure described for the preparation of **9**. The oxidation of **41** was quenched within 0.5 h to avoid over-oxidation.

## 4. Conclusions 

A simple strategy was devised for the synthesis of enantiomerically pure 2-substituted 2,5-dihydro-3-(arylsulfonyl)- and 2-substituted-2,5-dihydro-3-(arylsulfinyl)-furans from easily accessible carbohydrate derivatives. Since appendage diversity is one of the three major components of DOS [9], each of the four pure, and a diastereomeric mixture of, Michael acceptors were reacted with a variety of nucleophiles. The reactions were highly efficient, and each of **10**, **16**, **19R_S_**, and **19S_S_** afforded single diastereomers with varied appendages. The mixture of diastereomers **21** also afforded a pair of diastereomers from the Michael addition. Although the varying steric bulk at C-2 in combination with different nucleophiles could not alter the diastereoselectivity of addition, this strategy opens up a novel route for the synthesis of new enantiopure furanics with appendage diversity. In addition to the synthetic utility of this strategy, the other major observation is that the group, attached to a single chirality carbon (*i.e.*, C-2) originating from carbohydrate irrespective of its size, dictated the formation of all products in *anti-anti* configurations. Moreover, the group at C-2 suppressed the effect of chiral sulfoxides in the case of vinyl sulfoxide-modified tetrahydrofurans. This strategy is now currently pursued to generate different sets of densely functionalized tetrahydrofurans using a DOS strategy. 

## Supplementary Materials

Supplementary materials can be accessed at: http://www.mdpi.com/1420-3049/21/6/690/s1.
