# Supplementary material for: Enantiopure Trisubstituted Tetrahydrofurans with Appendage Diversity: Vinyl Sulfone- and Vinyl Sulfoxide-Modified Furans Derived from Carbohydrates as Synthons for Diversity Oriented Synthesis
Source: Molecules. 2016 May 26;21(6):690. doi: 10.3390/molecules21060690 (PMC6274046; doi:10.3390/molecules21060690)
Supplement: Supplementary file 1 [file molecules-21-00690-s001.pdf]

Supplementary Materials: Enantiopure Trisubstituted Tetrahydrofurans with Appendage Diversity: Vinyl Sulfone- and Vinyl Sulfoxide-Modified Furans Derived from Carbohydrates as Synthons for Diversity Oriented Synthesis

Debanjana Dey and Tanmaya Pathak

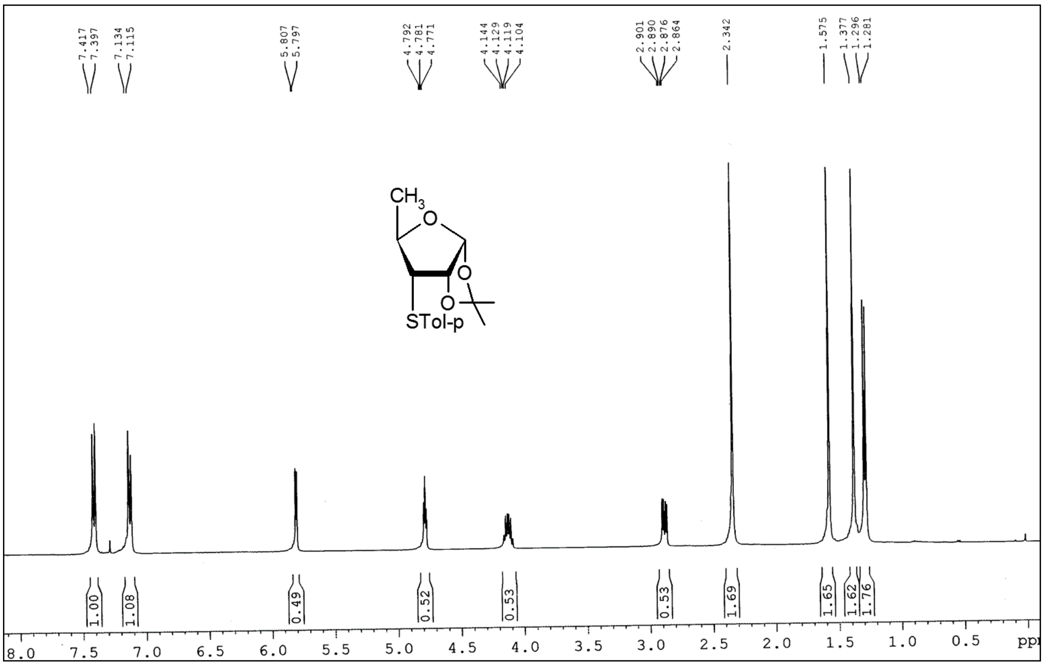

Figure S1. ^{1}H-NMR spectrum of compound **6**.

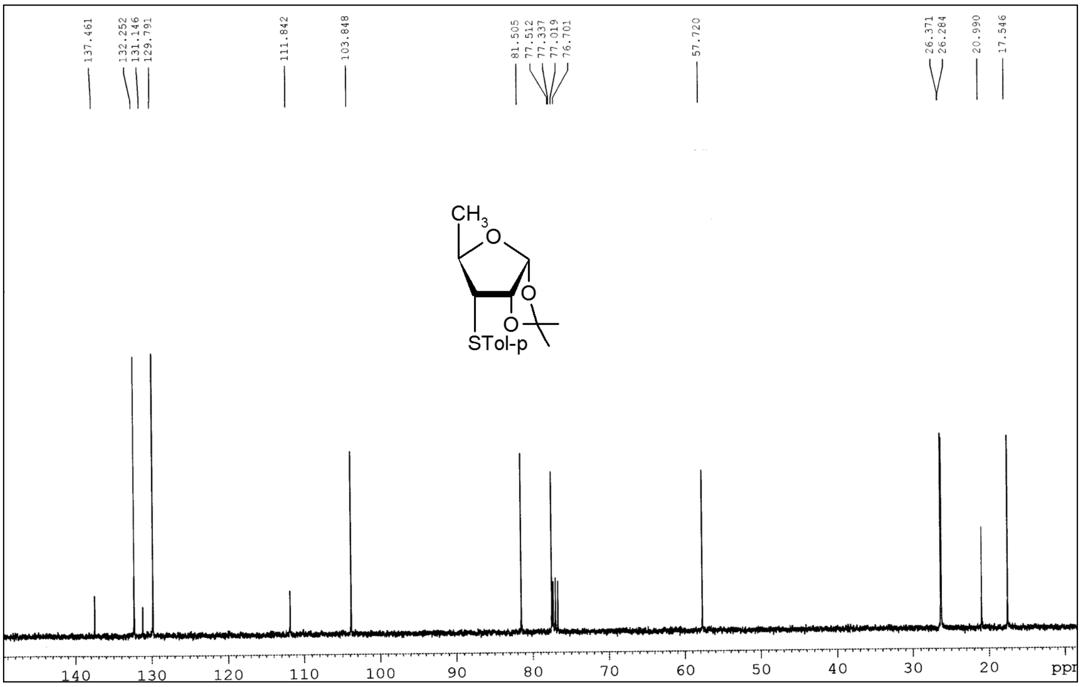

Figure S2. ^{13}C-NMR spectrum of compound **6**.

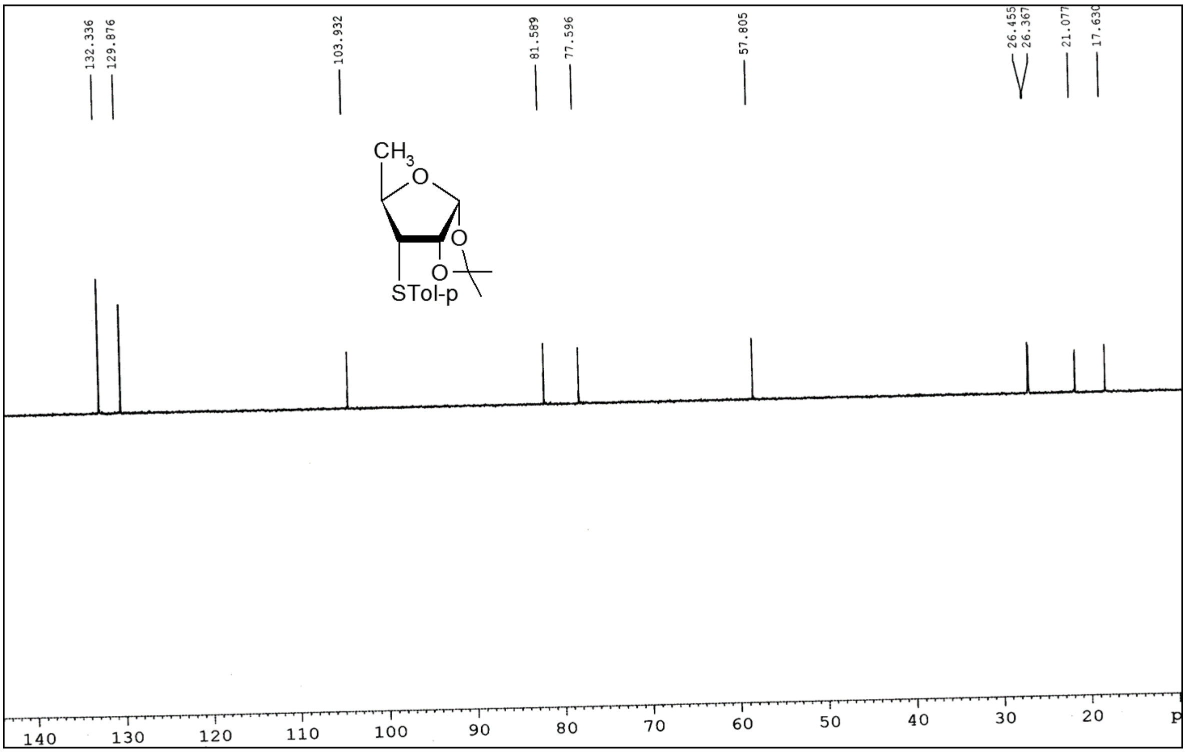

Figure S3. DEPT spectrum of compound **6**.

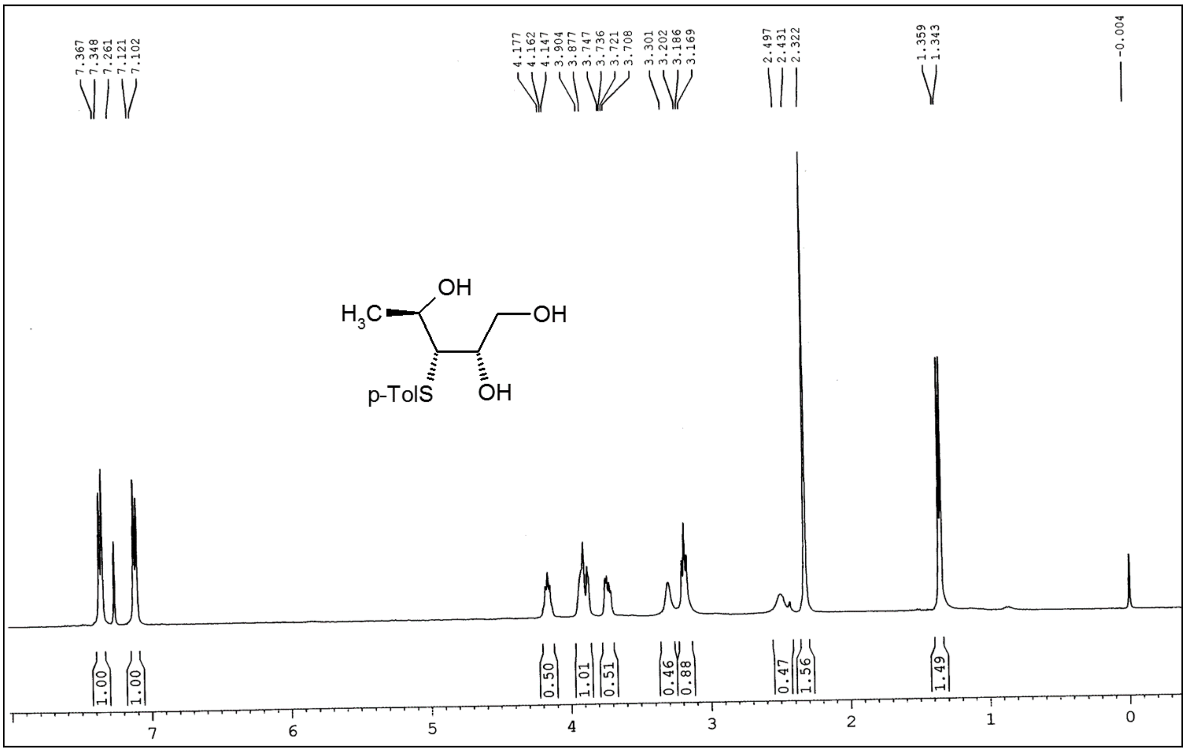

Figure S4. ^{1}H-NMR spectrum of compound **7**.

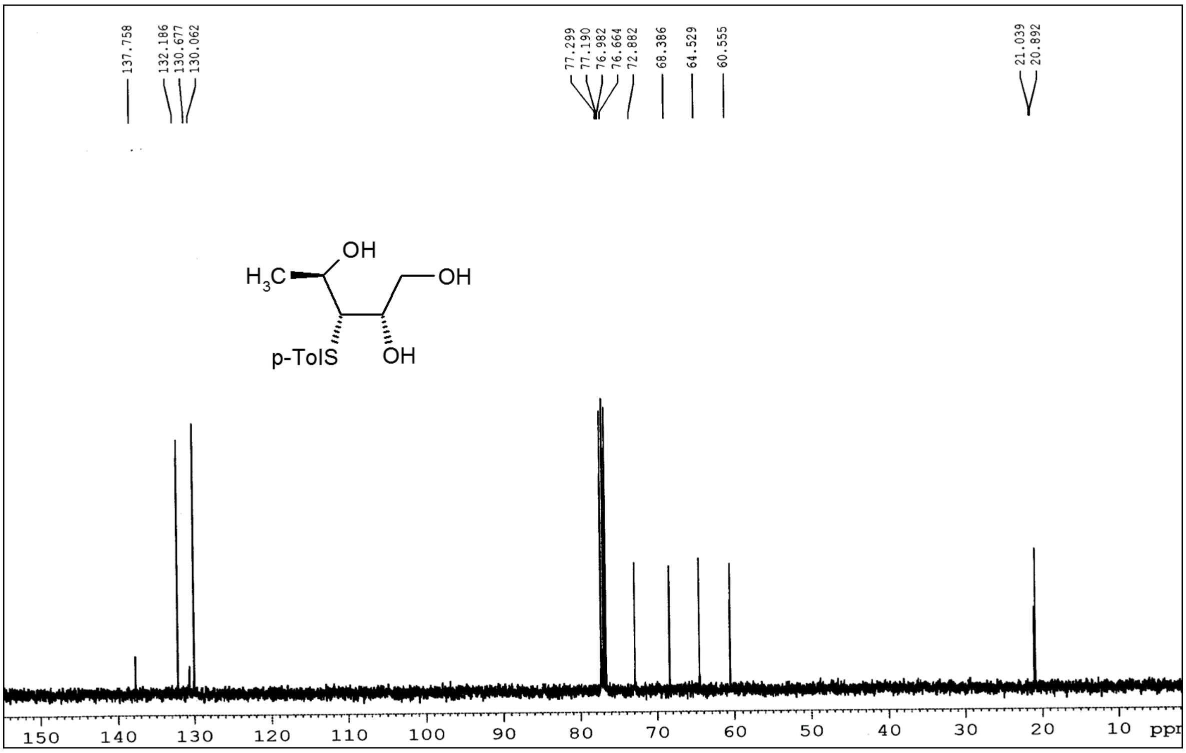

Figure S5. ^{13}C-NMR spectrum of compound **7**.

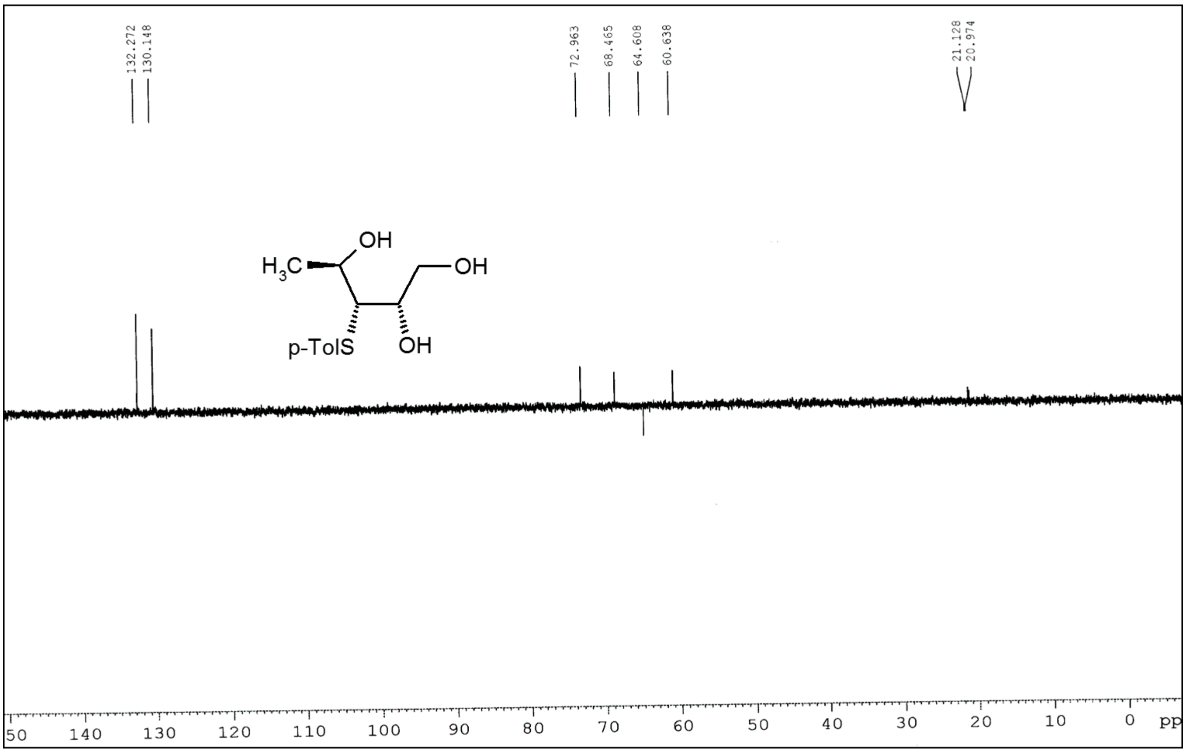

Figure S6. DEPT spectrum of compound **7**.

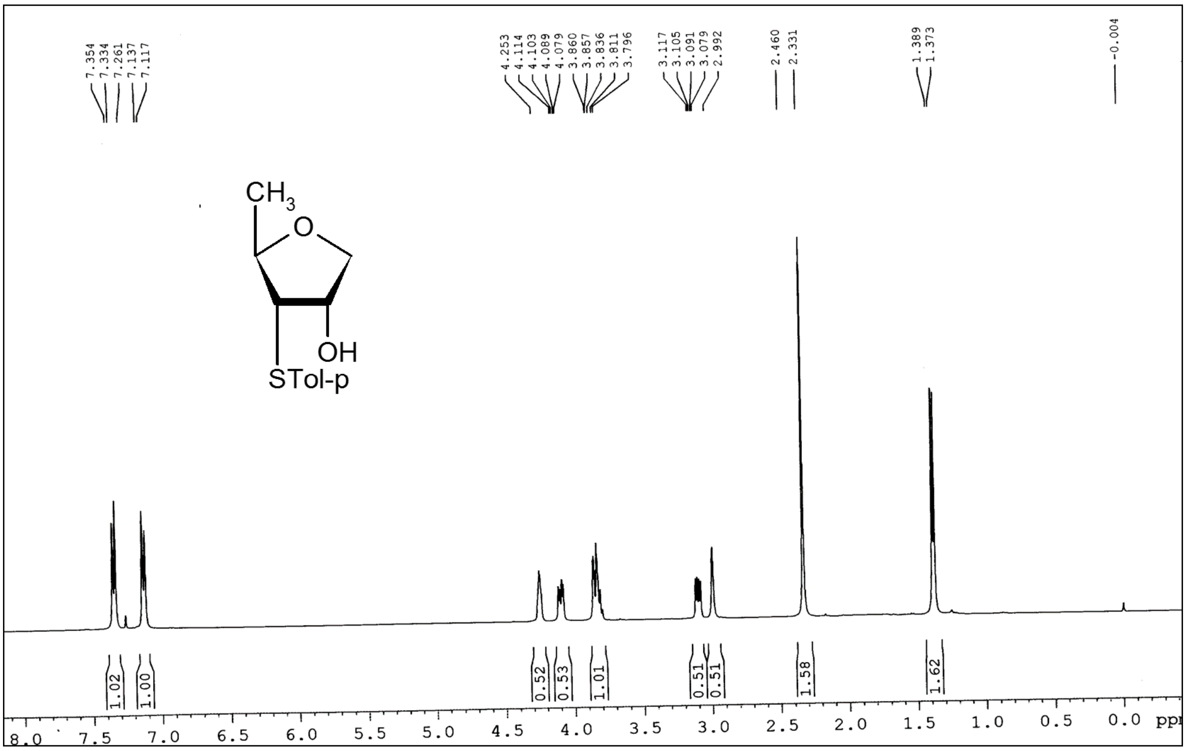

Figure S7. ^{1}H-NMR spectrum of compound **8**.

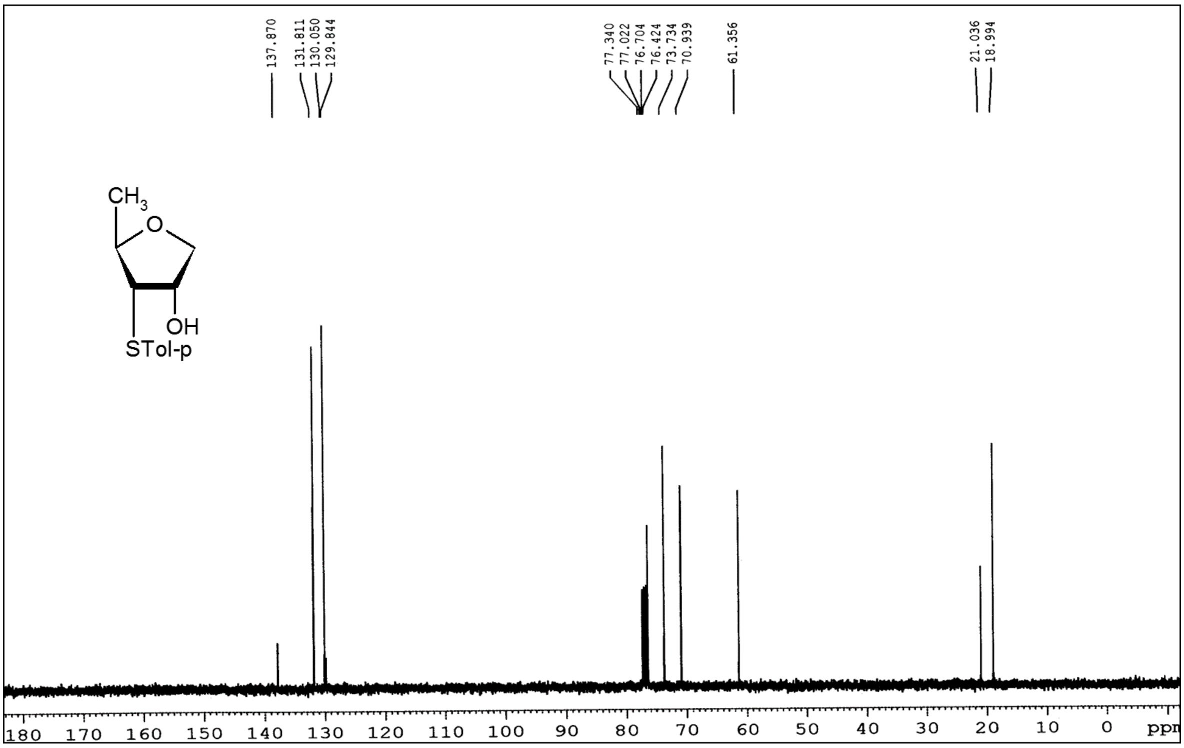

Figure S8. ^{13}C-NMR spectrum of compound **8**.

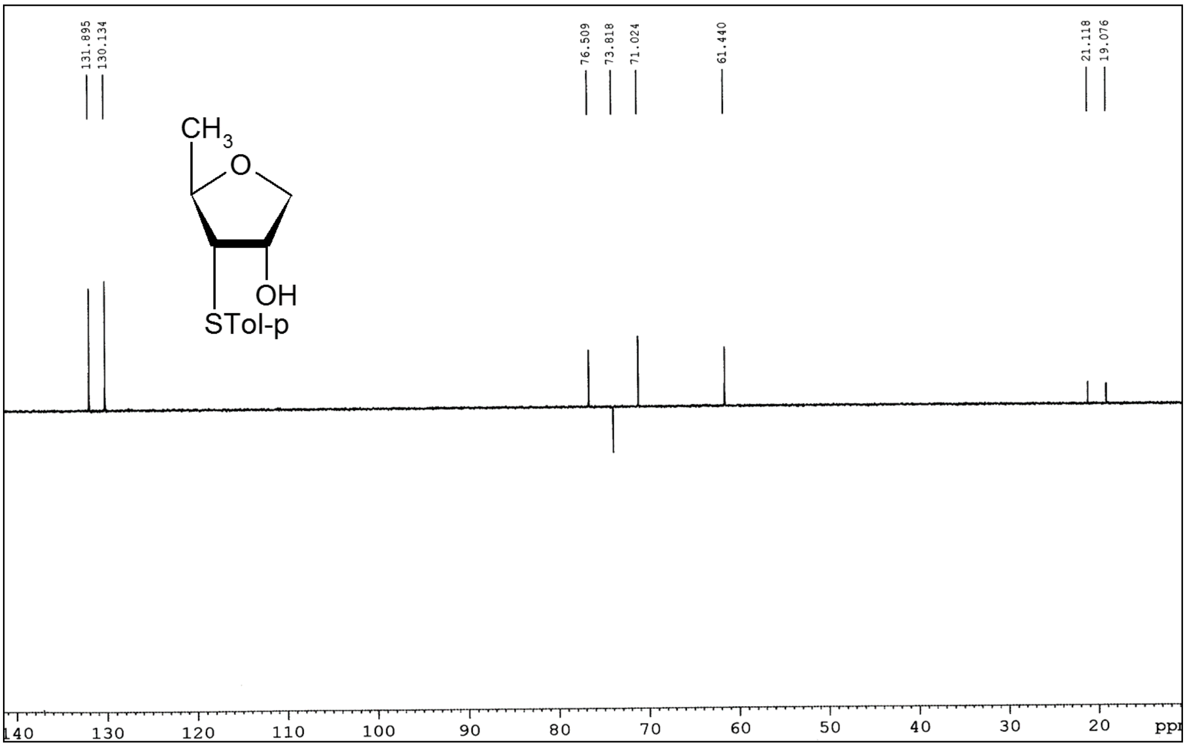

Figure S9. DEPT spectrum of compound **8**.

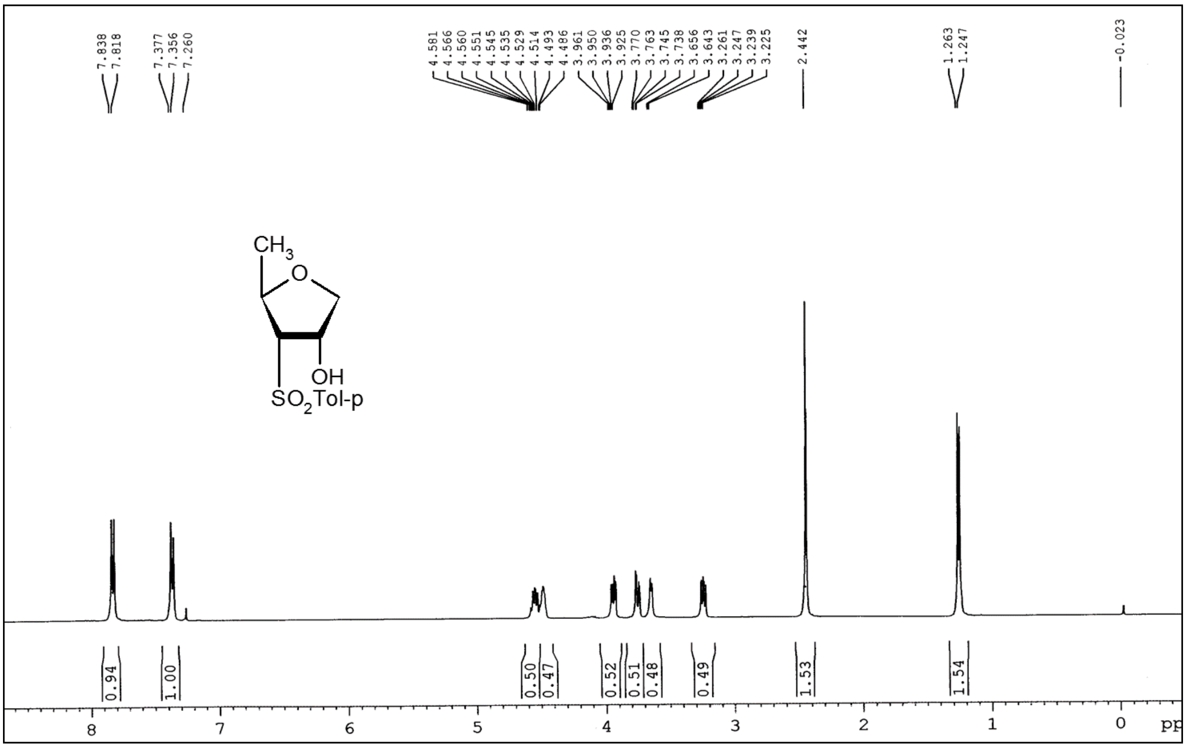

Figure S10. ^{1}H-NMR spectrum of compound **9**.

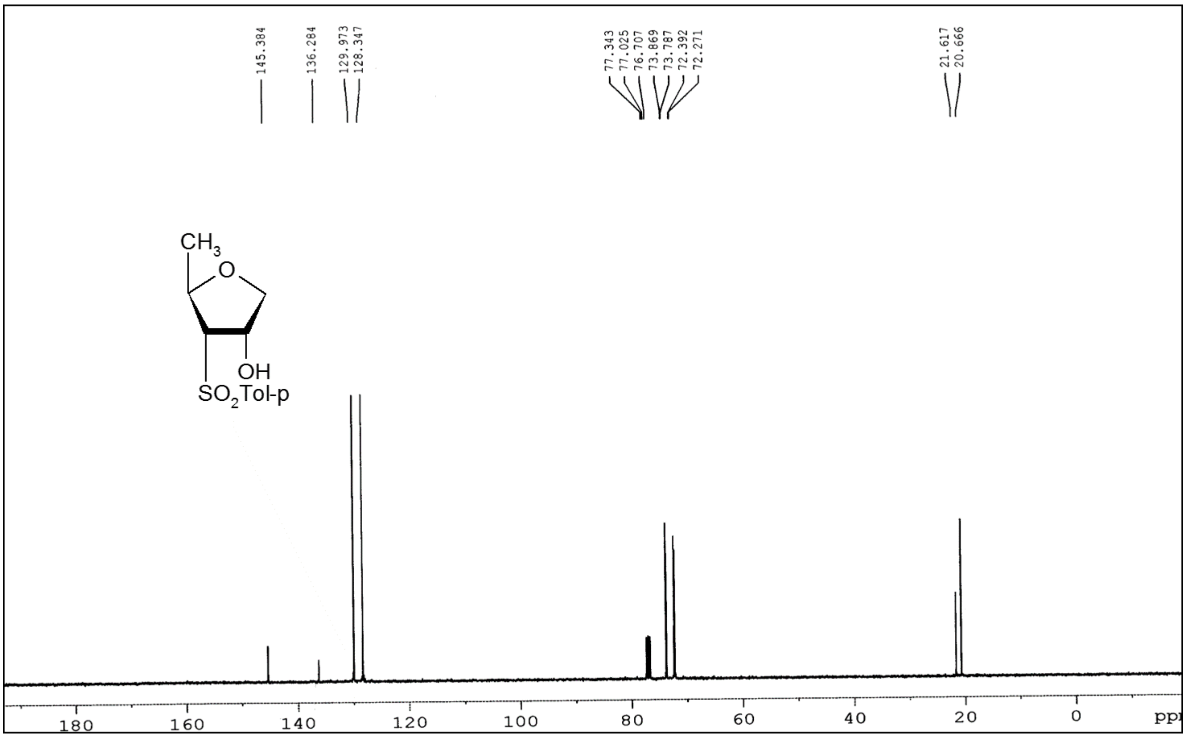

Figure S11. ^{13}C-NMR spectrum of compound **9**.

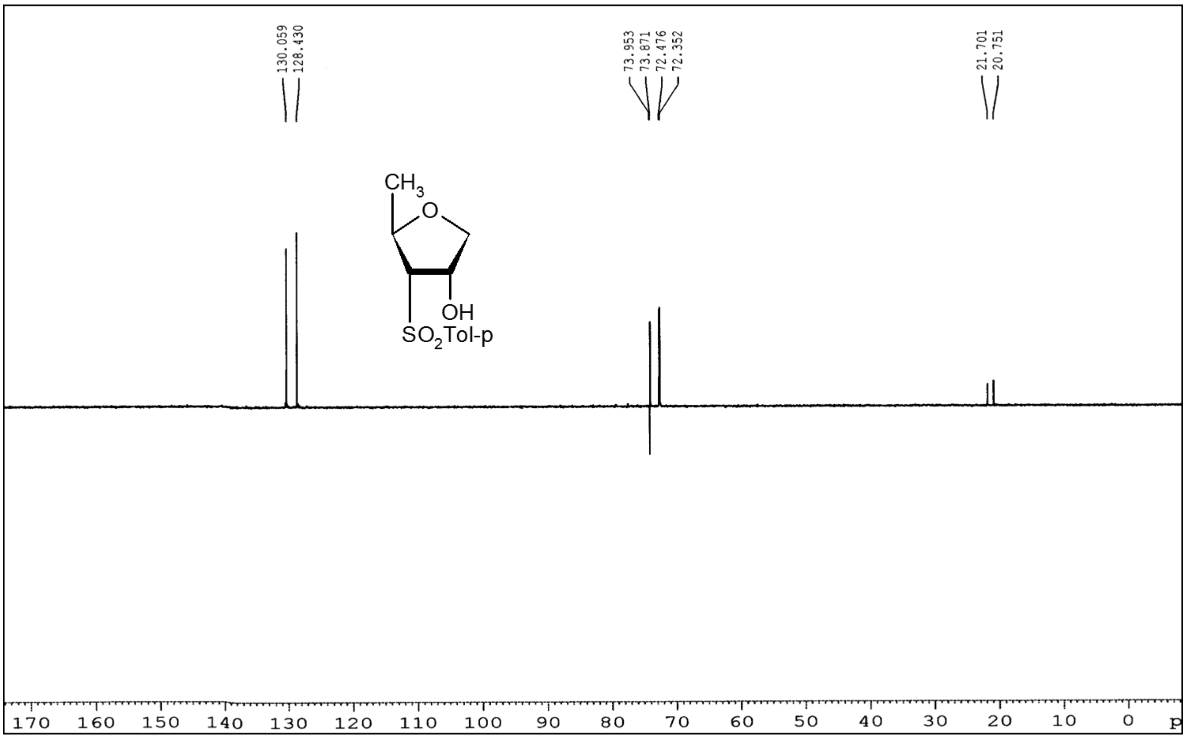

Figure S12. DEPT spectrum of compound **9**.

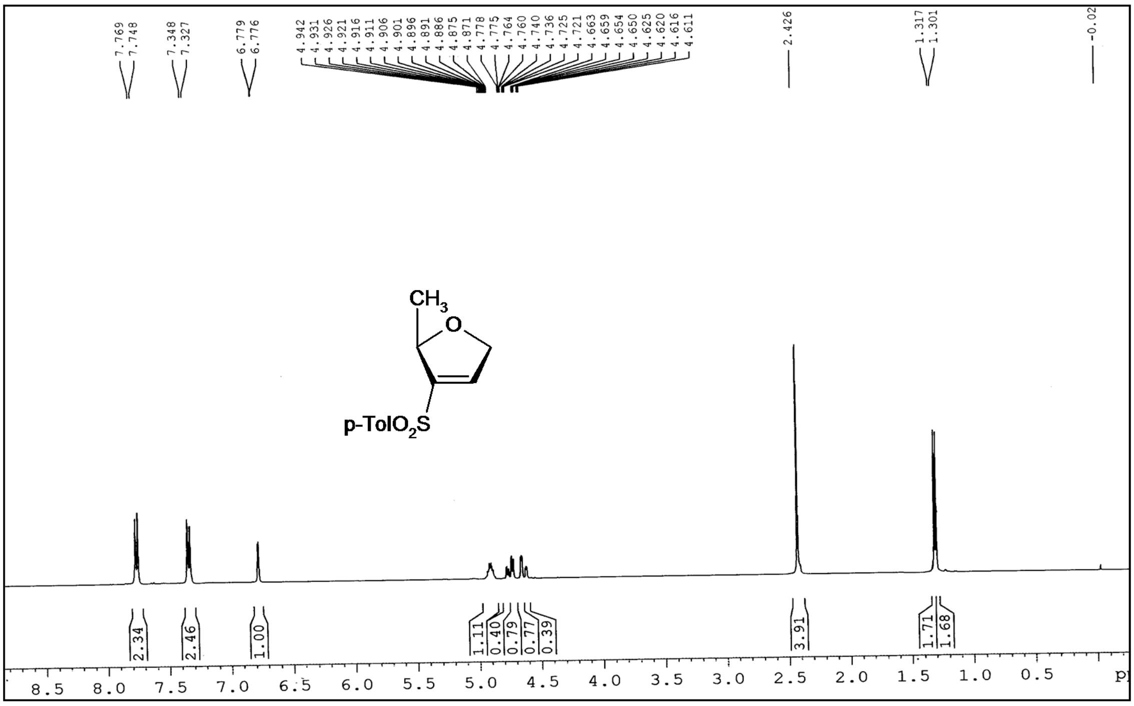

Figure S13. ^{1}H-NMR spectrum of compound **10**.

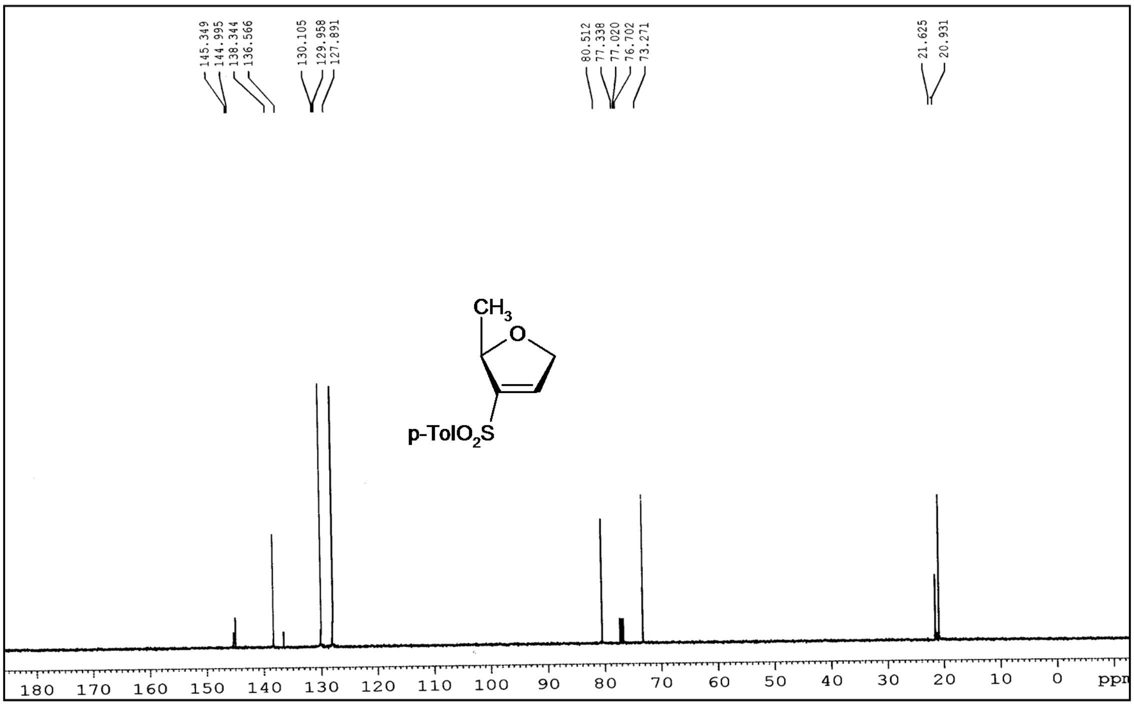

Figure S14. ^{13}C-NMR spectrum of compound **10**.

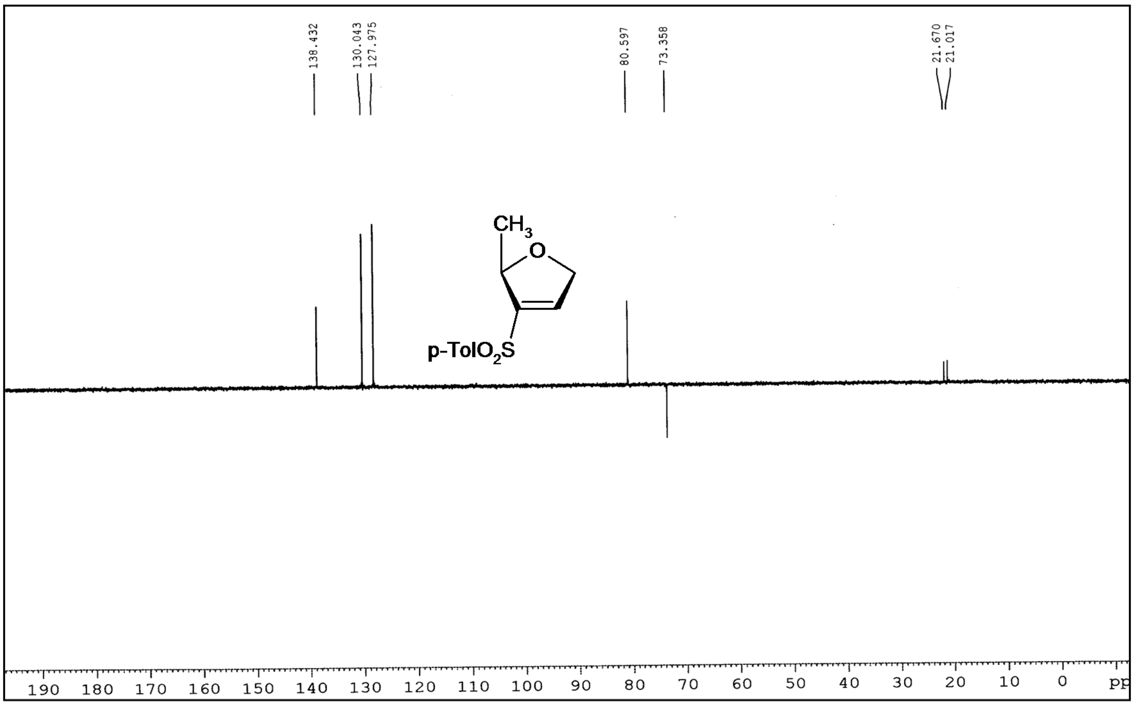

Figure S15. DEPT spectrum of compound **10**.

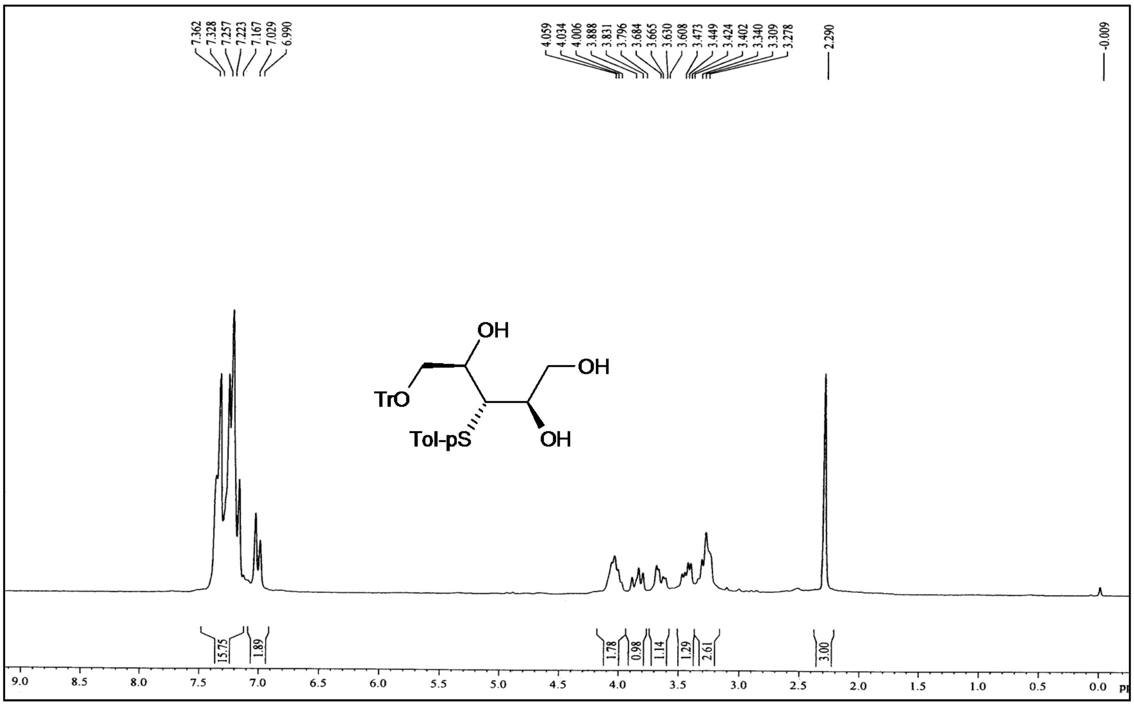

Figure S16. ^{1}H-NMR spectrum of compound **13**.

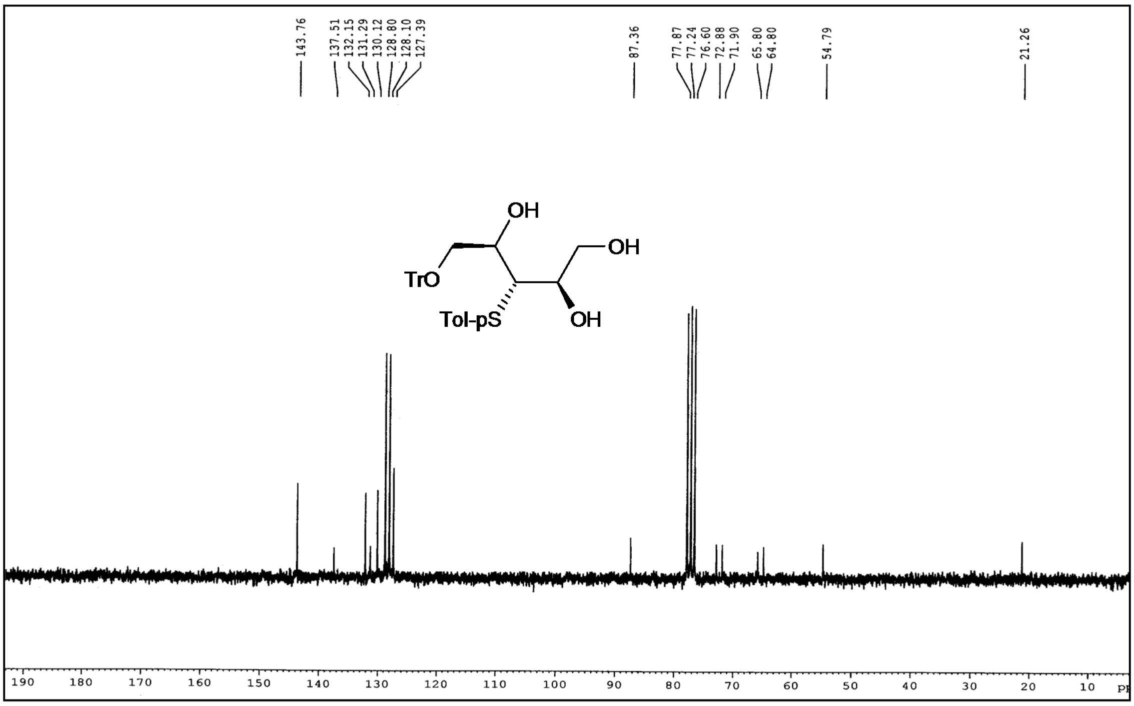

Figure S17. ^{13}C-NMR spectrum of compound **13**.

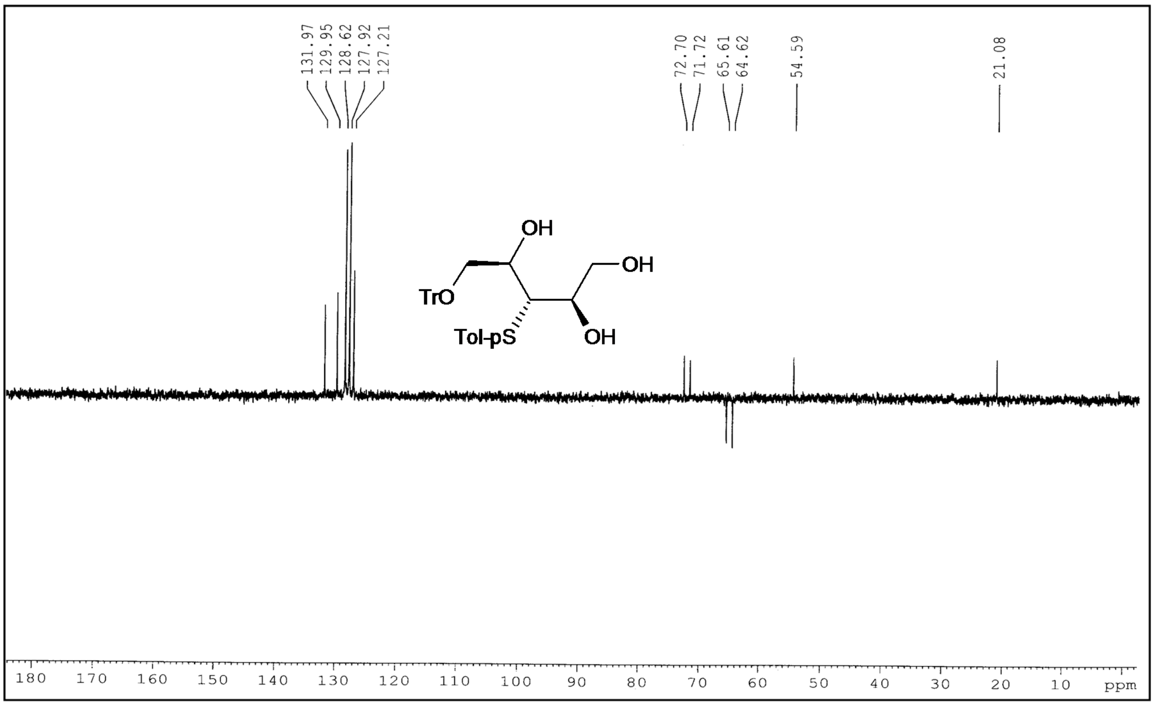

Figure S18. DEPT spectrum of compound **13**.

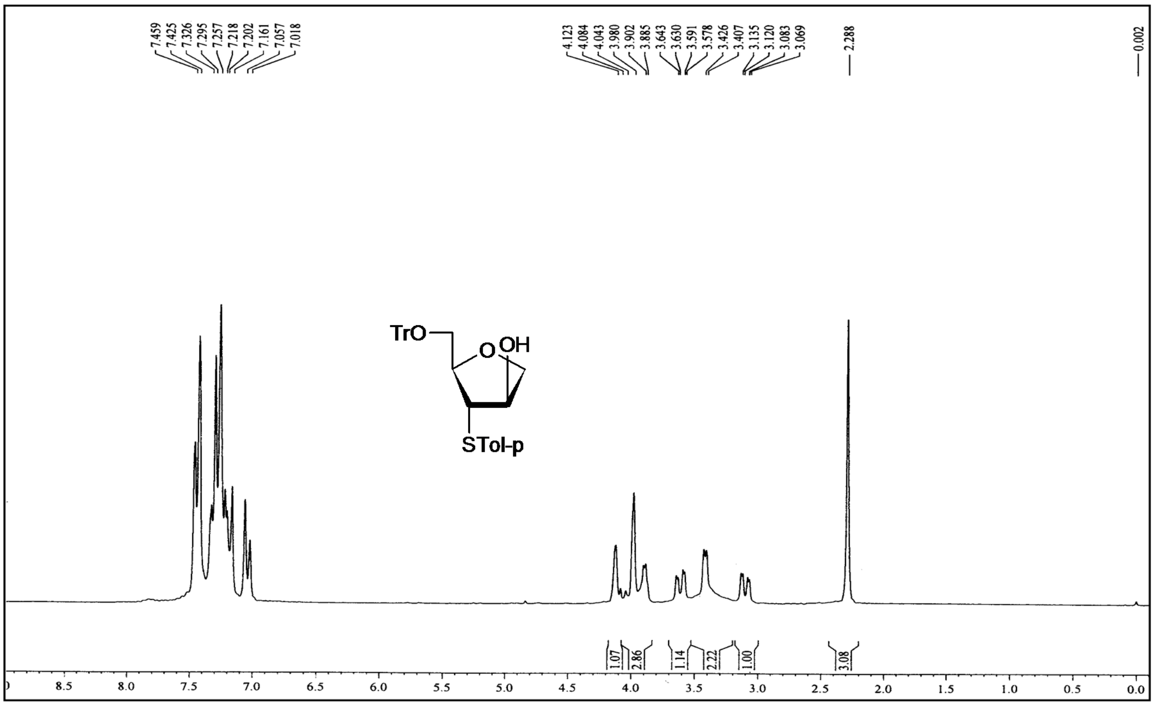

Figure S19. ^{1}H-NMR spectrum of compound **14**.

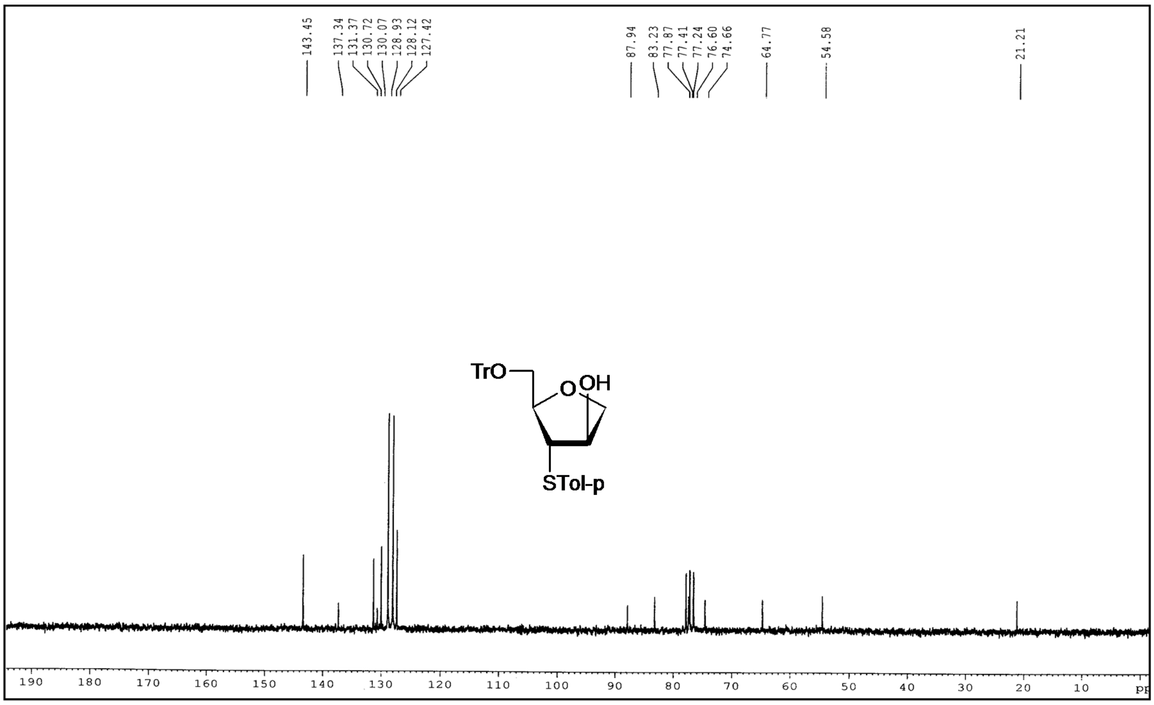

Figure S20. ^{13}C-NMR spectrum of compound **14**.

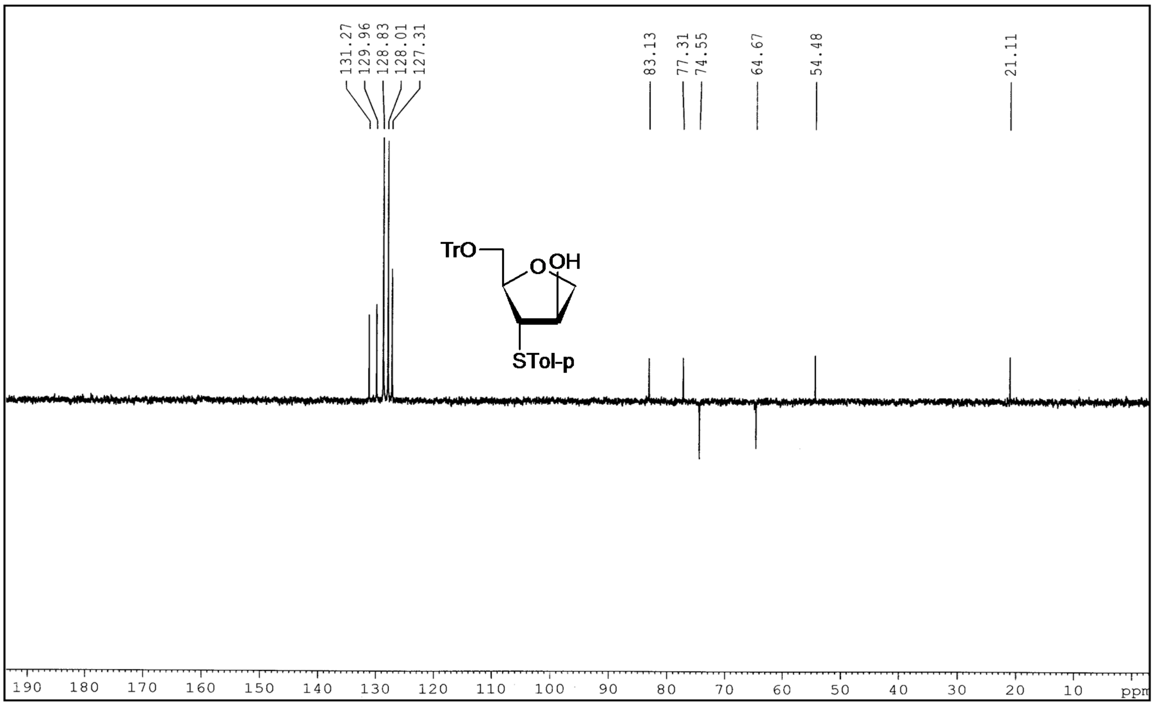

Figure S21. DEPT spectrum of compound **14**.

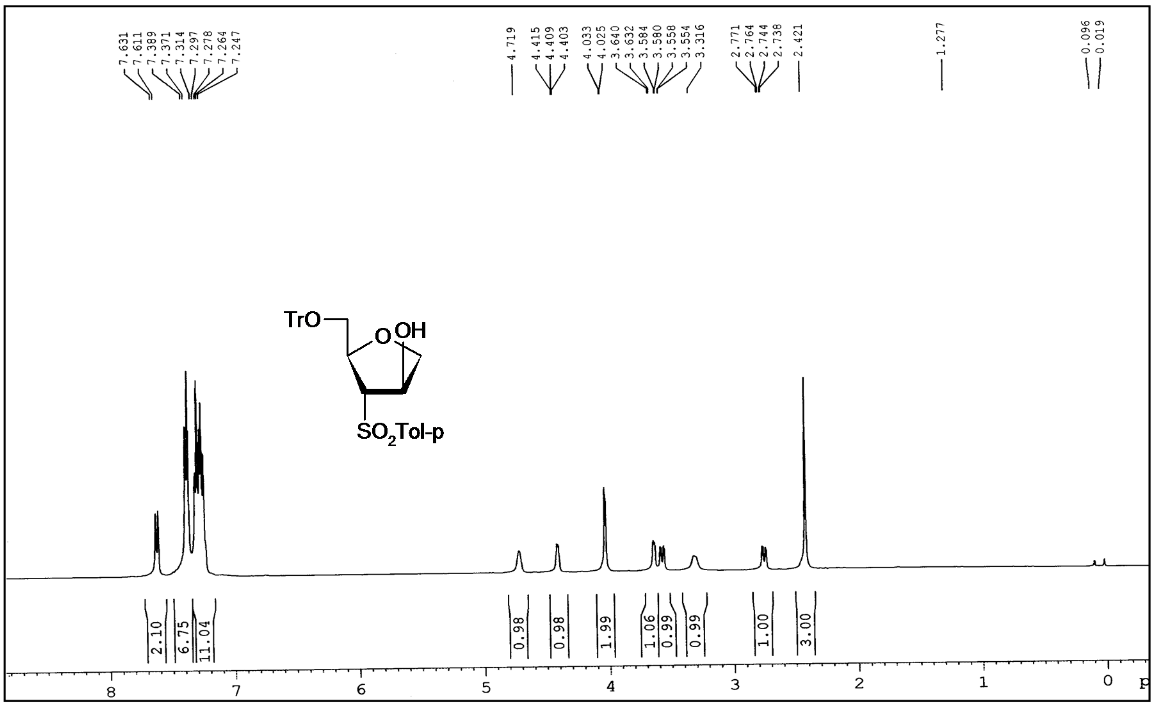

Figure S22. ^{1}H-NMR spectrum of compound **15**.

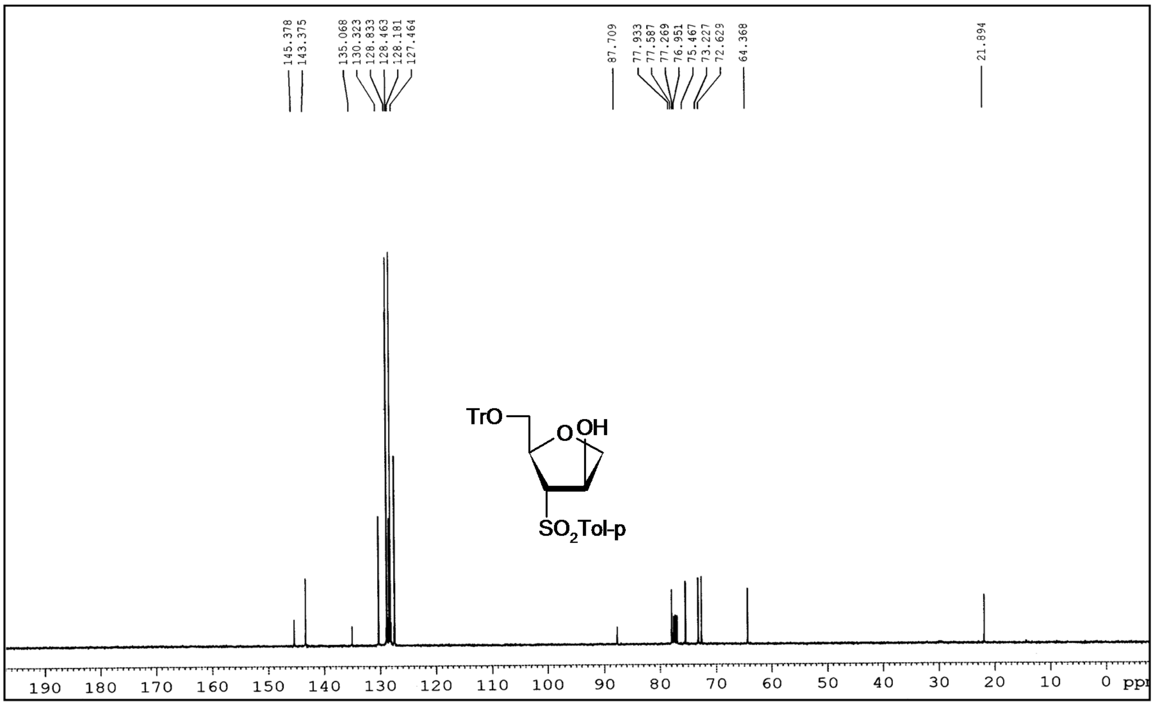

Figure S23. ^{13}C-NMR spectrum of compound **15**.

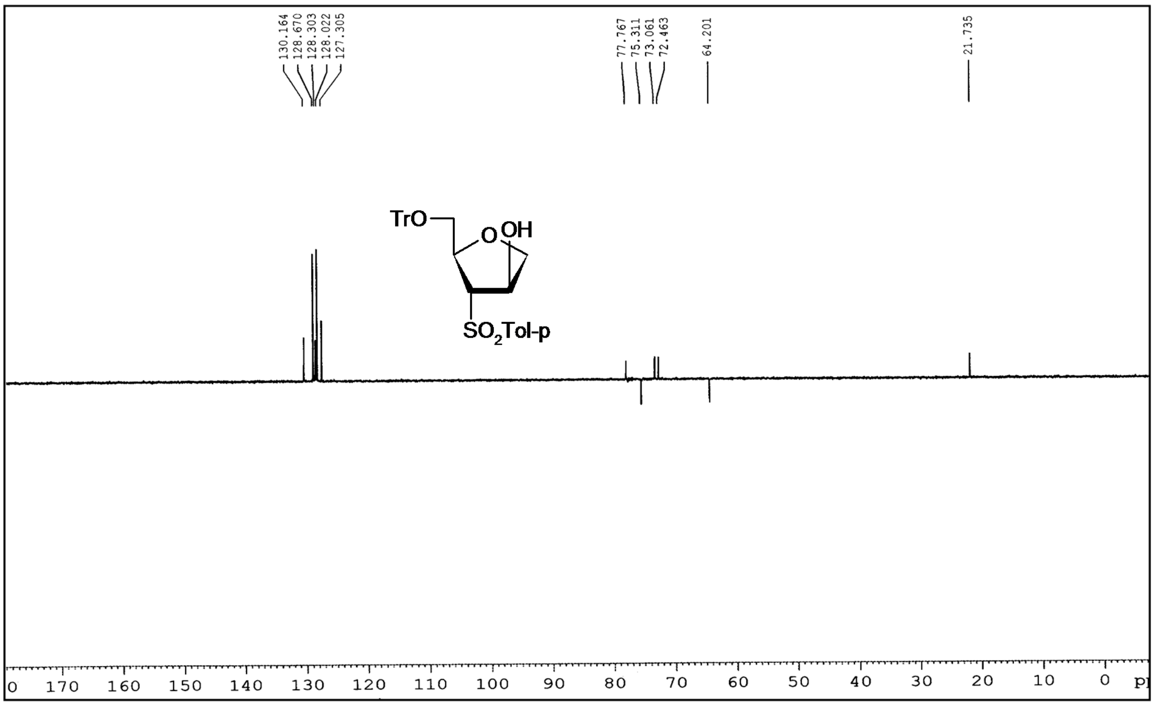

Figure S24. DEPT spectrum of compound **15**.

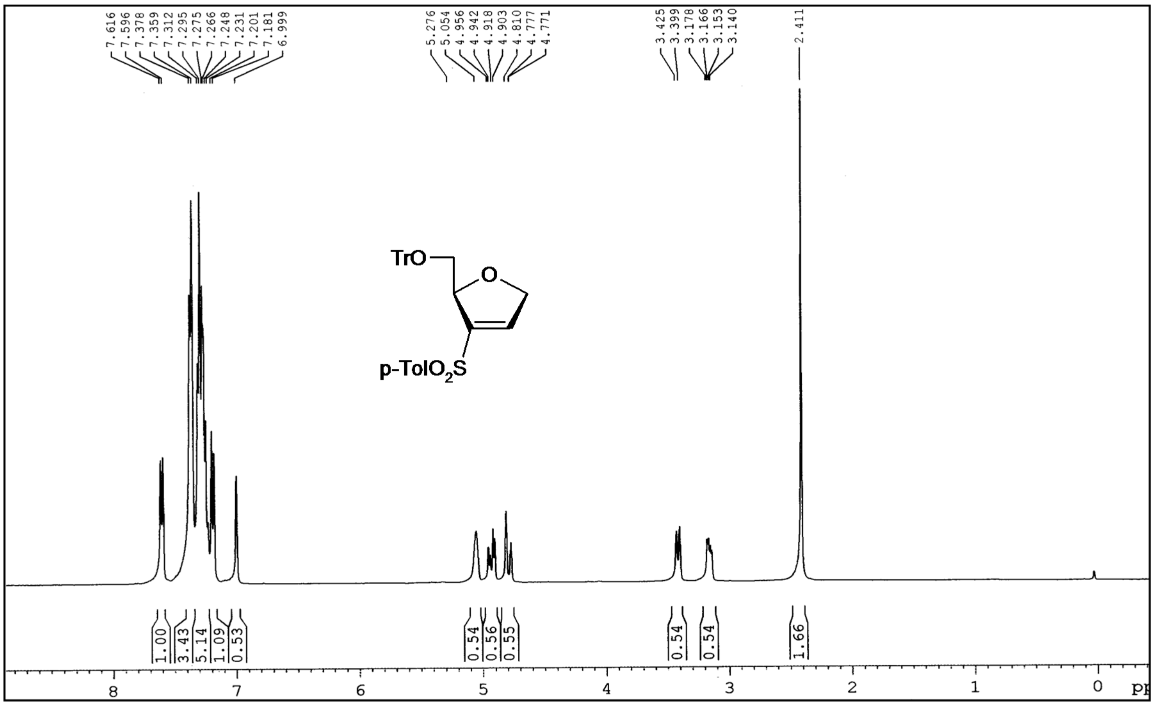

Figure S25. ^{1}H-NMR spectrum of compound **16**.

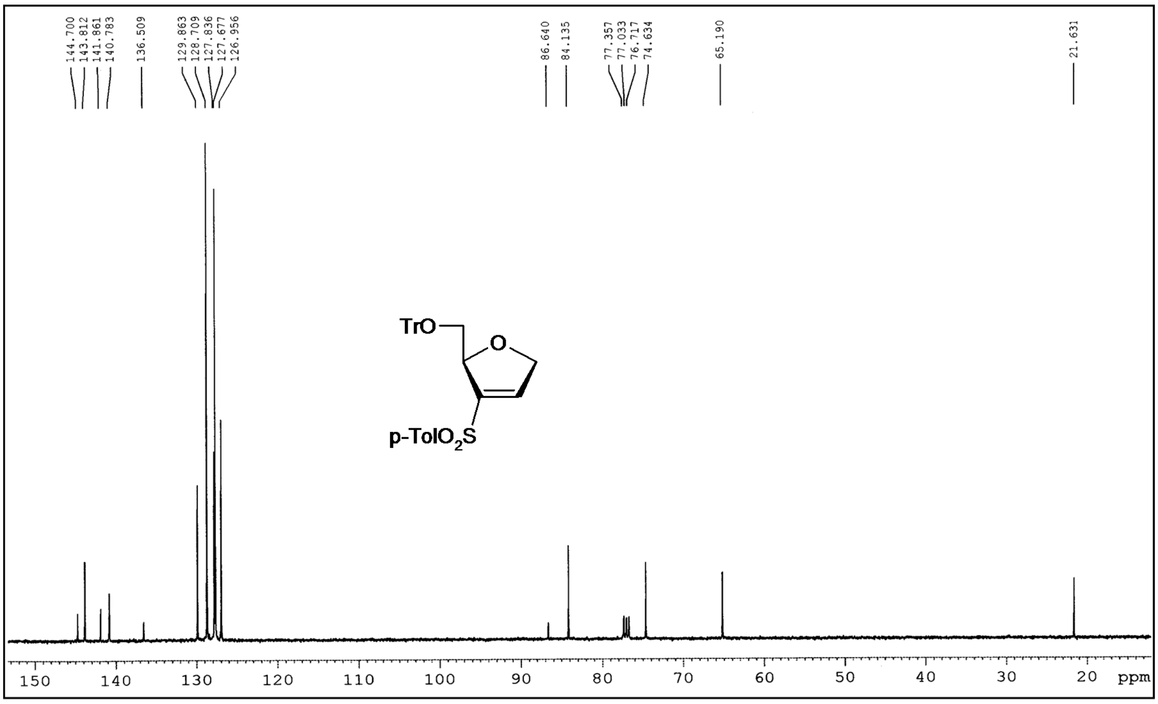

Figure S26. ^{13}C-NMR spectrum of compound **16**.

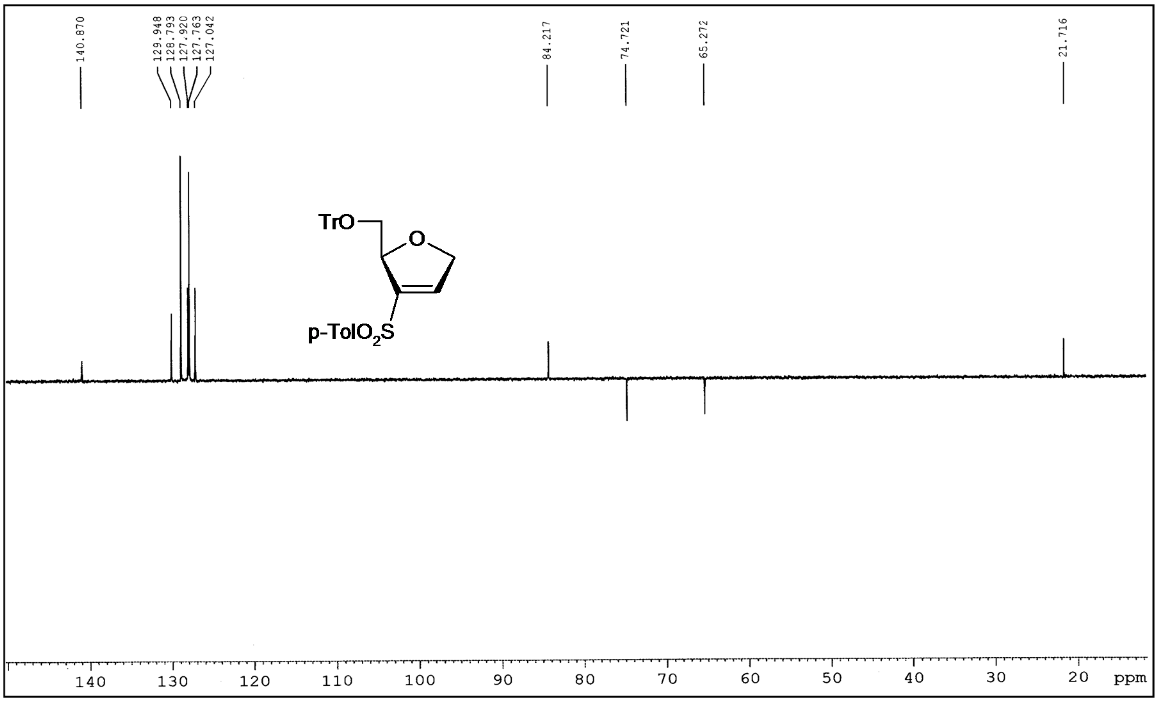

Figure S27. DEPT spectrum of compound **16**.

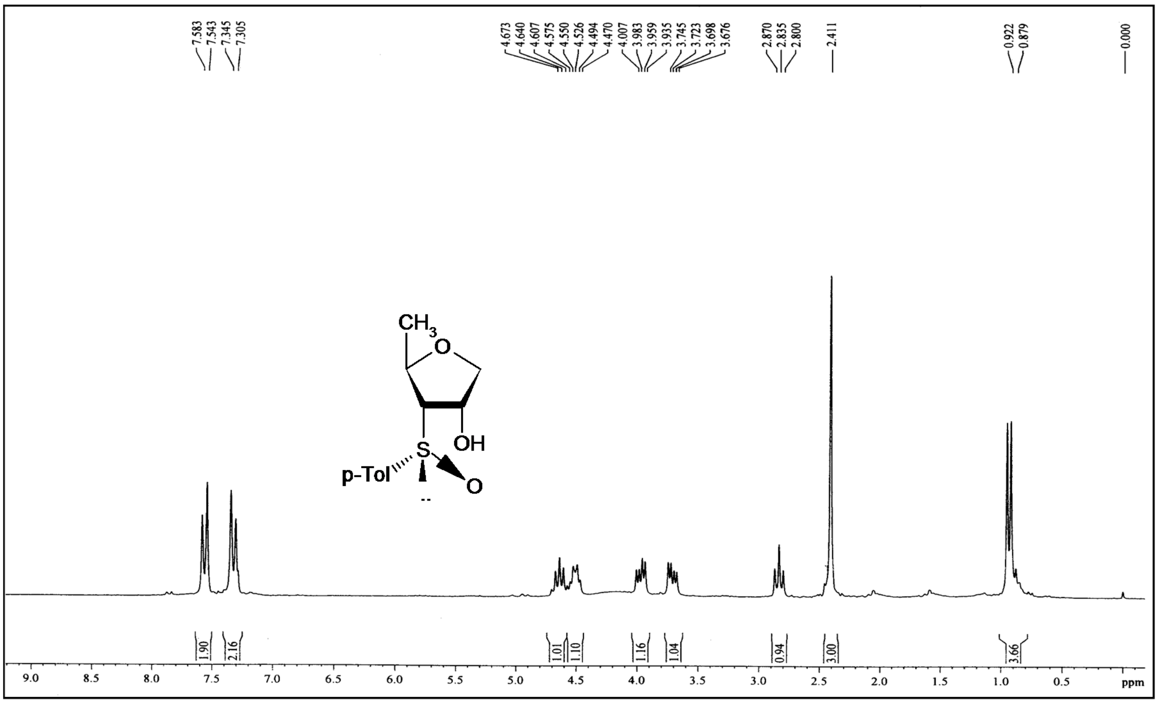

Figure S28. ^{1}H-NMR spectrum of compound **17Ss**.

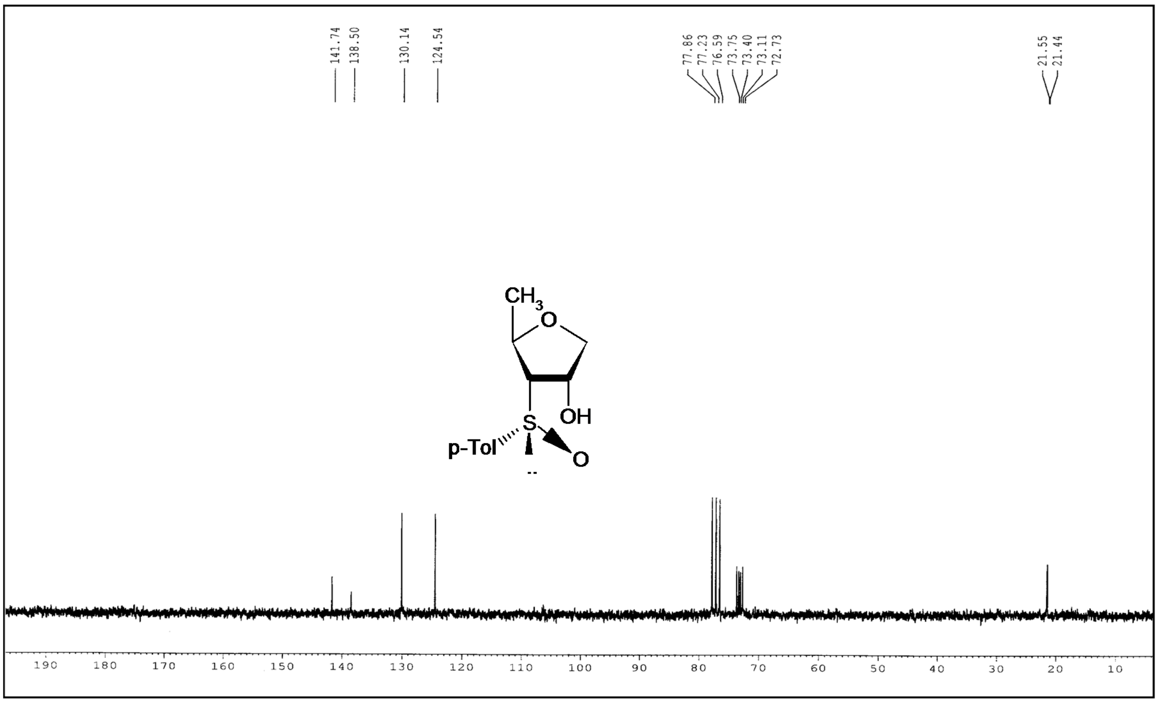

Figure S29. ^{13}C-NMR spectrum of compound **17S**s.

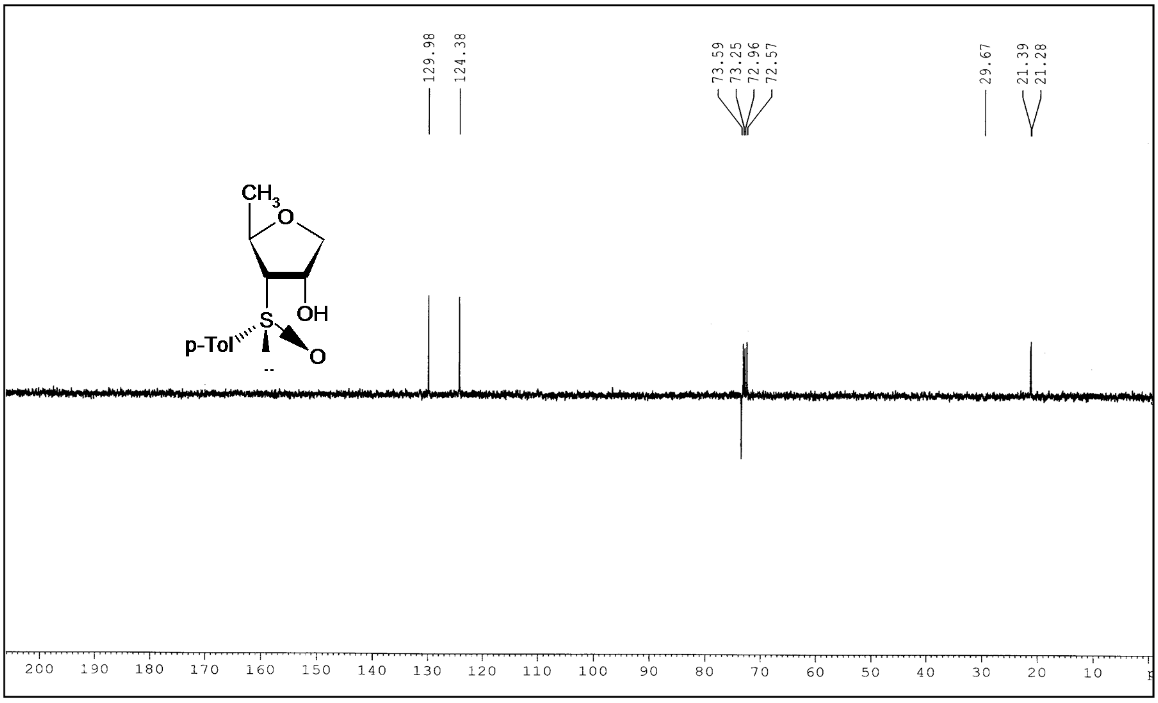

Figure S30. DEPT spectrum of compound **17S**s.

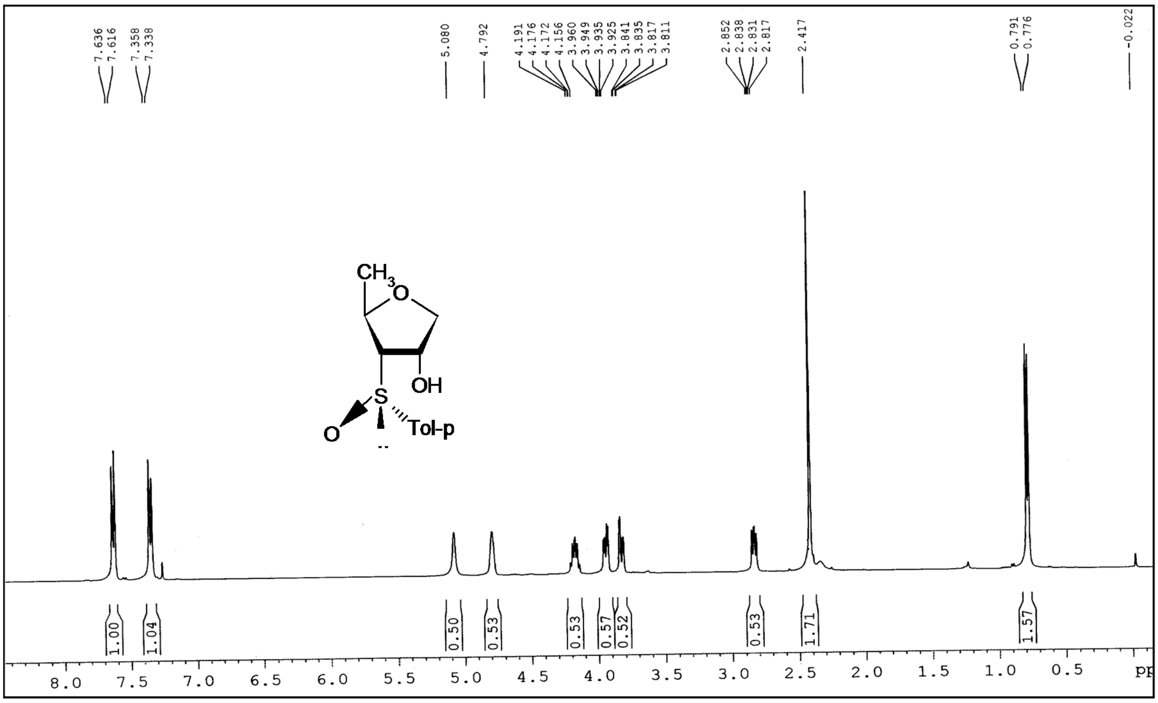

Figure S31. ^{1}H-NMR spectrum of compound **17Rs**.

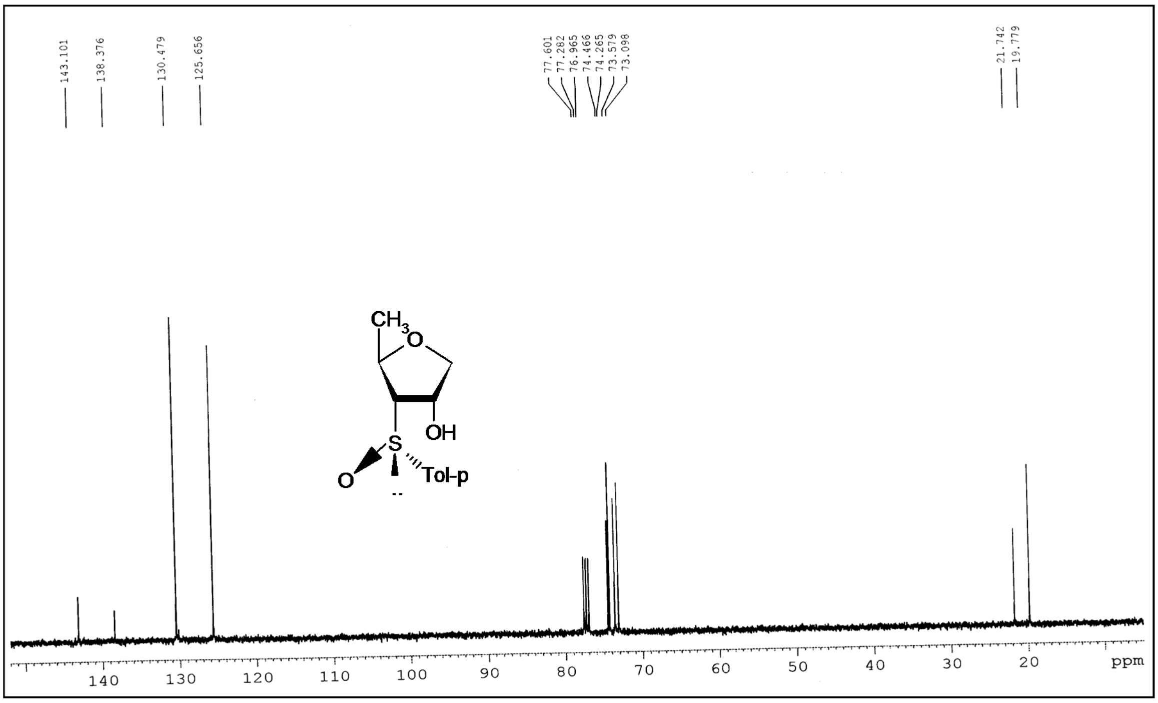

Figure S32. ^{13}C-NMR spectrum of compound **17Rs**.

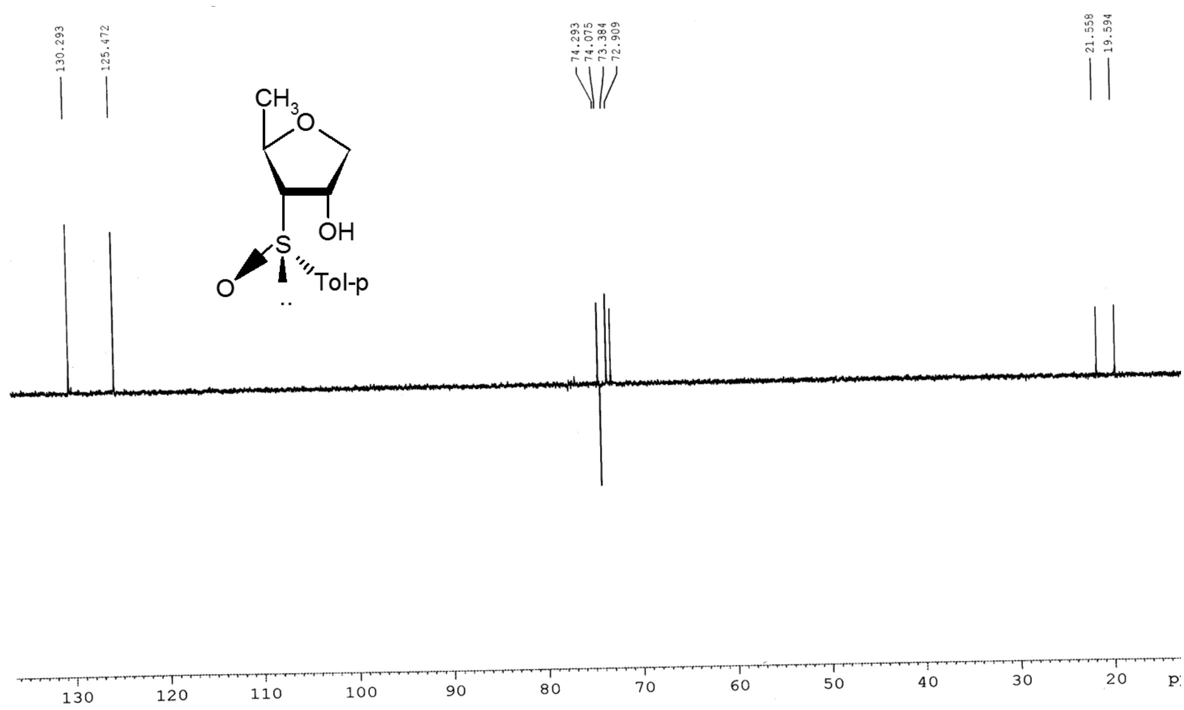

Figure S33. DEPT spectrum of compound **17R**s.

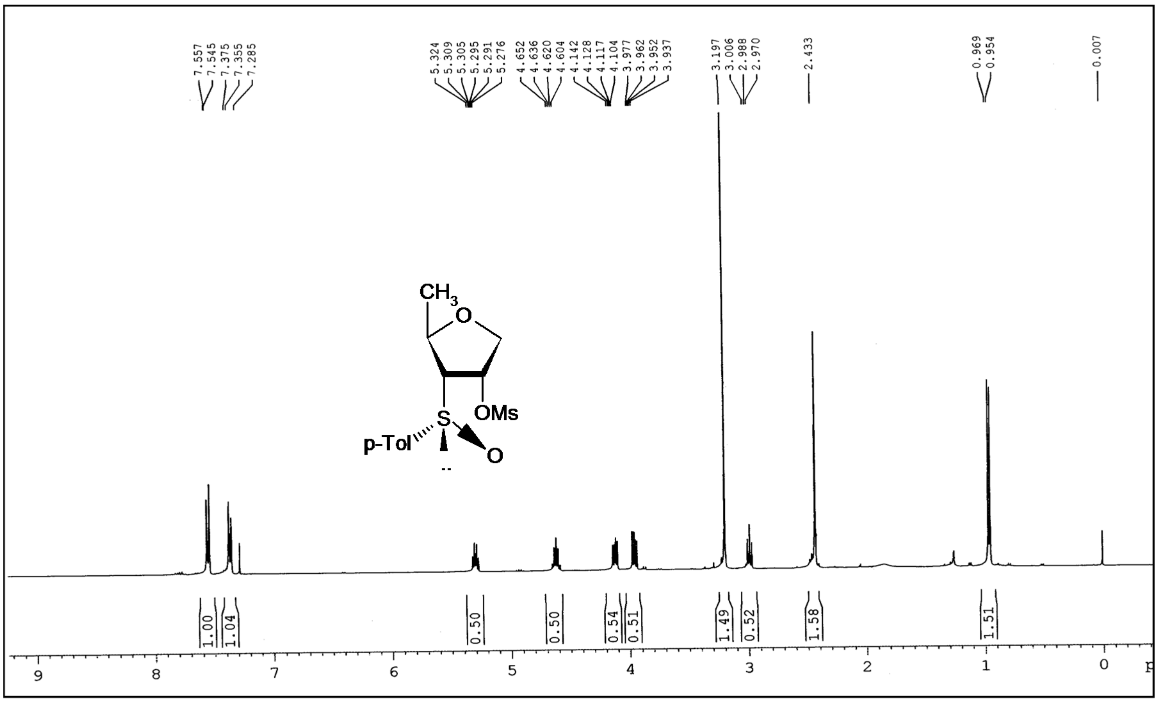

Figure S34. ^{1}H-NMR spectrum of compound **18S**s.

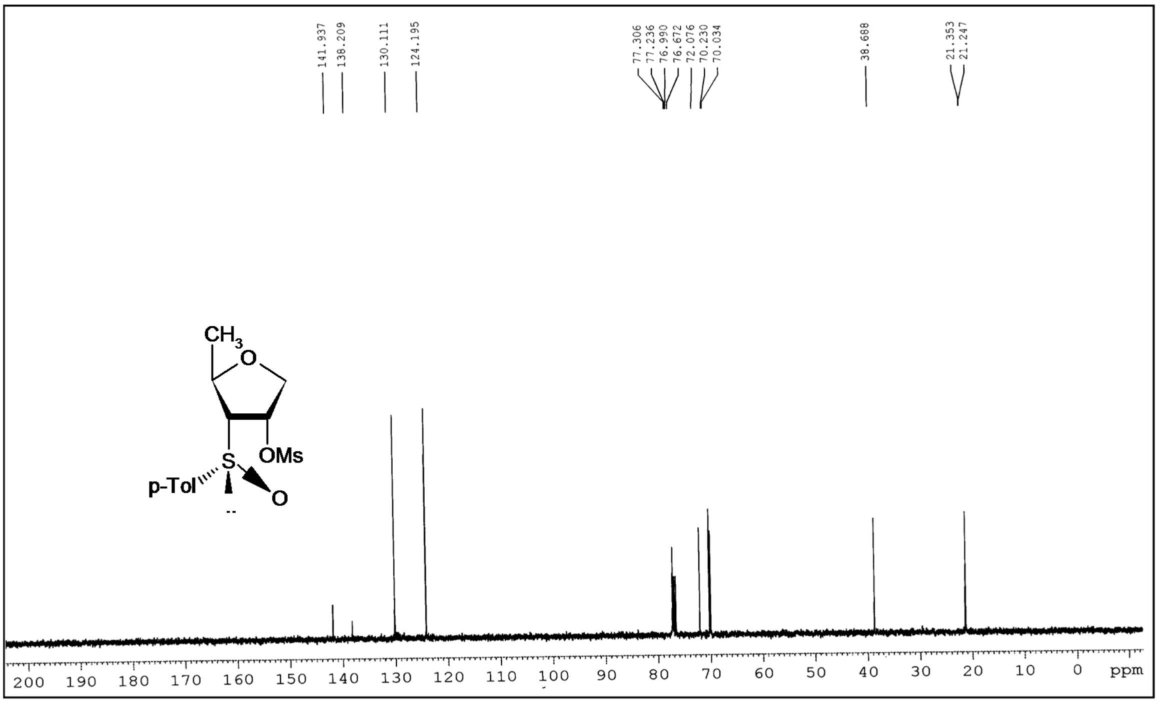

Figure S35. ^{13}C-NMR spectrum of compound **18S**s.

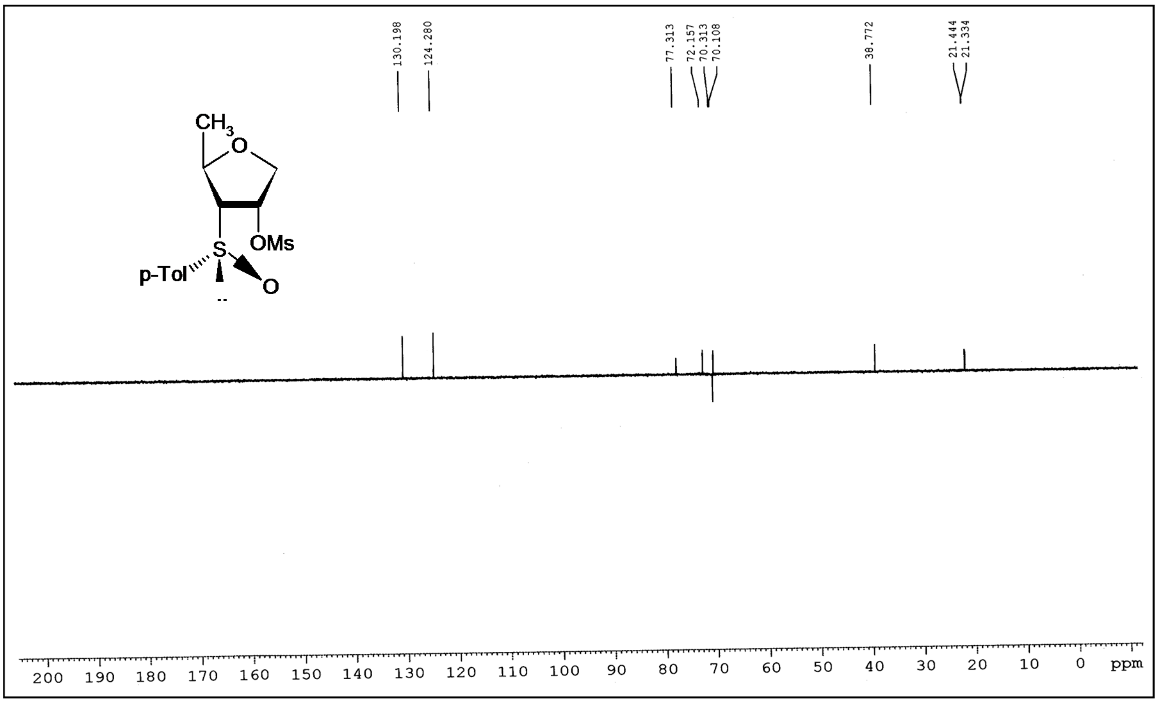

Figure S36. DEPT spectrum of compound **18S**s.

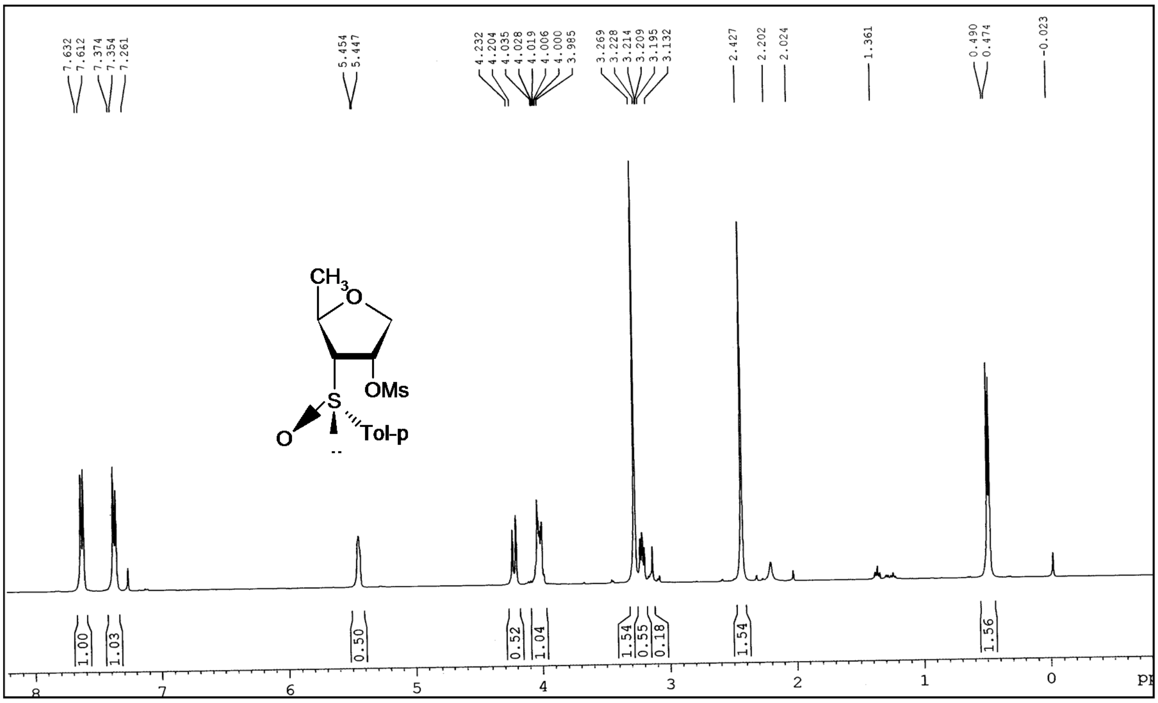

Figure S37. ^{1}H-NMR spectrum of compound **18R**s.

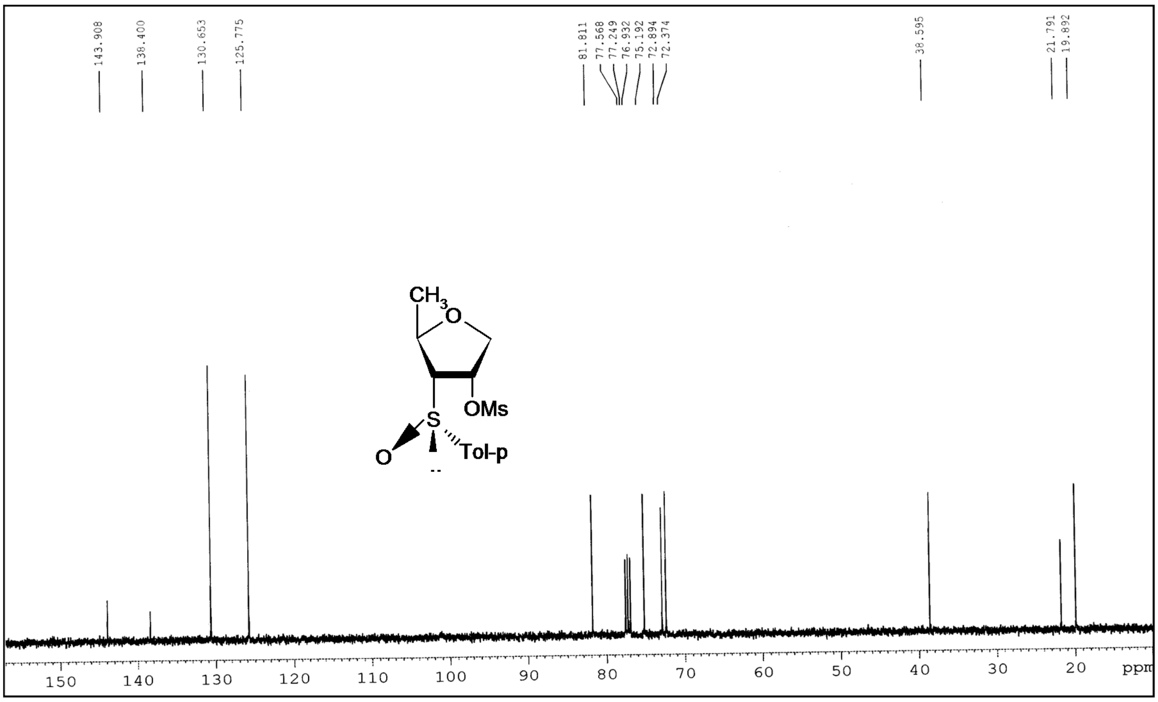

Figure S38. ^{13}C-NMR spectrum of compound **18R**s.

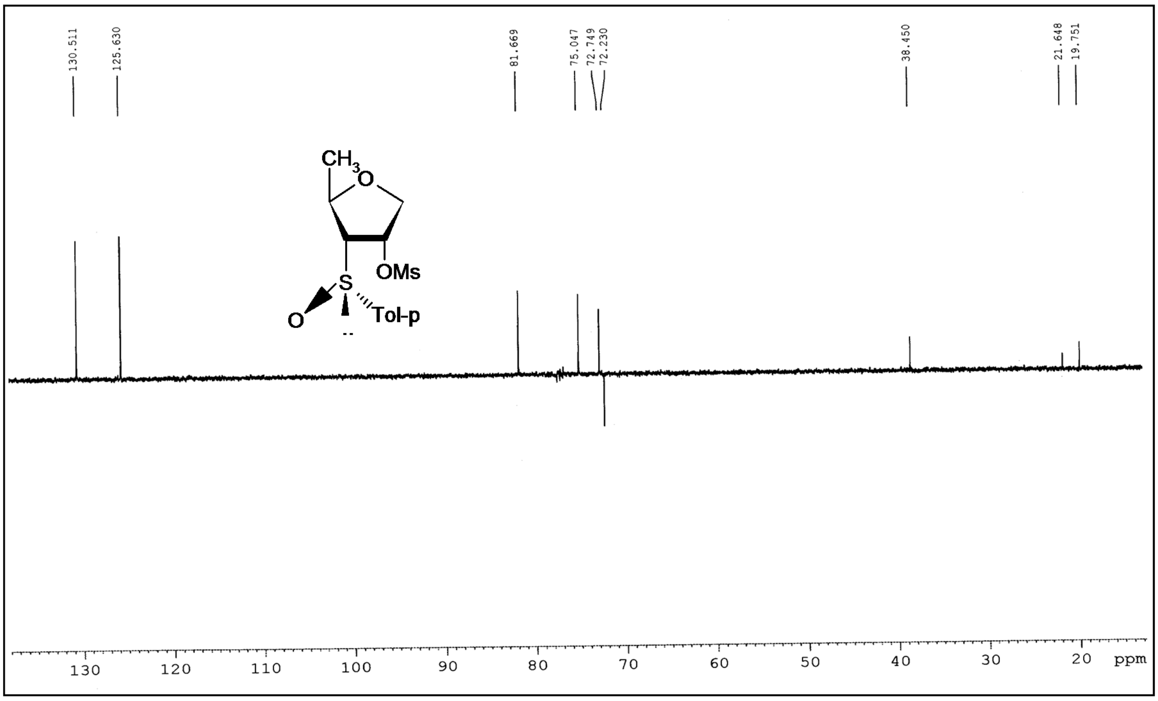

Figure S39. DEPT spectrum of compound **18R**s.

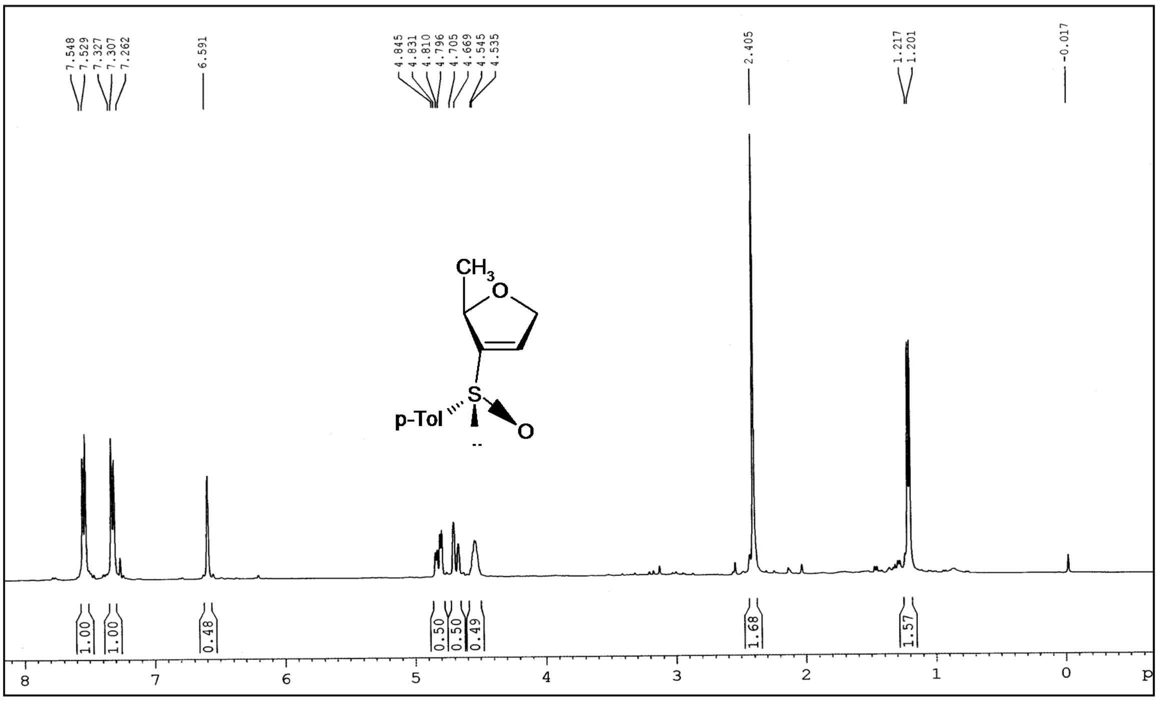

Figure S40. ^{1}H-NMR spectrum of compound **19R**s.

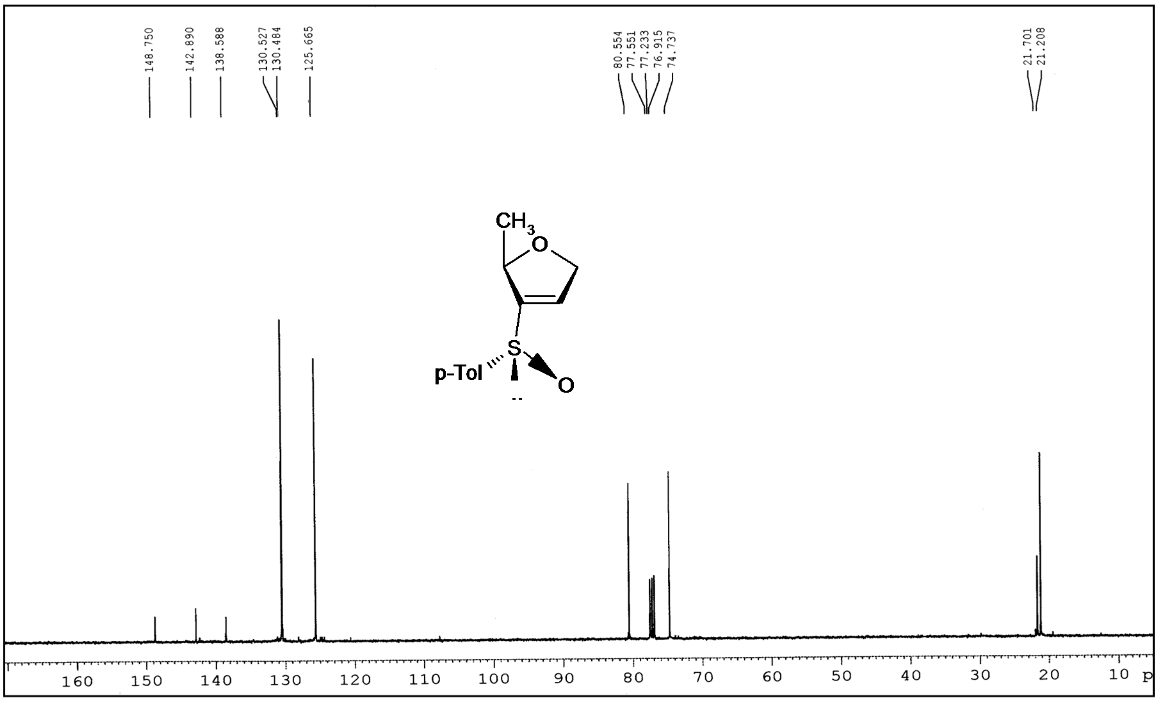

Figure S41. ^{13}C-NMR spectrum of compound **19R**s.

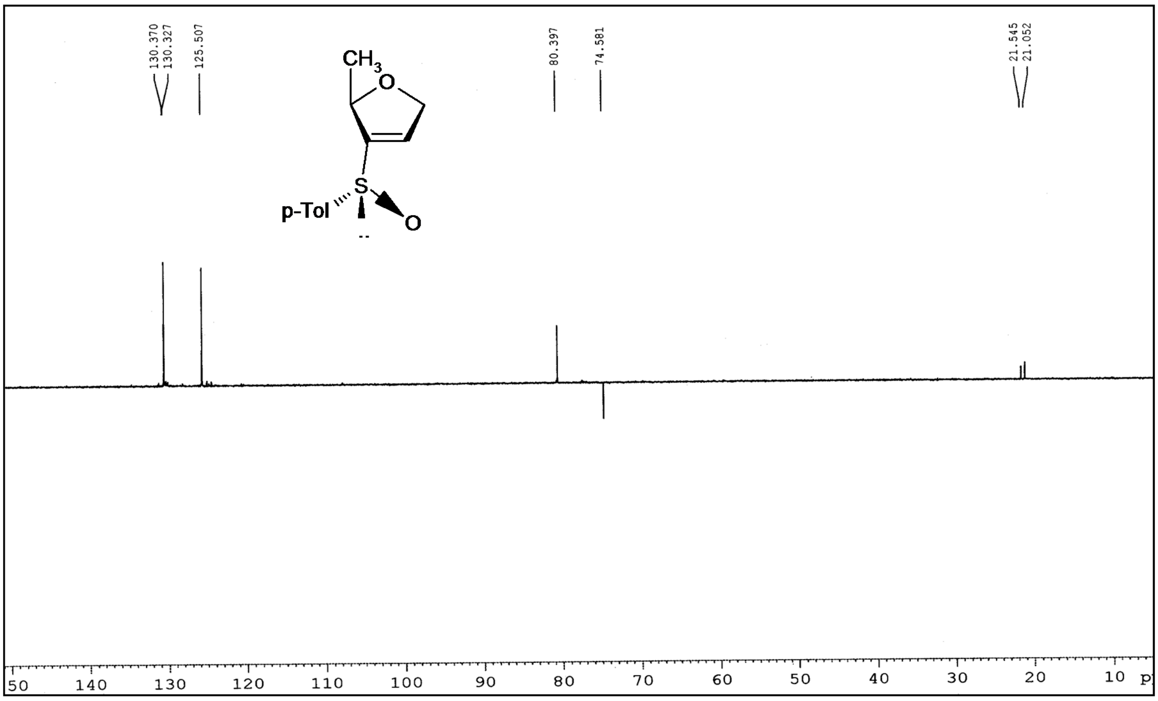

Figure S42. DEPT spectrum of compound **19R**s.

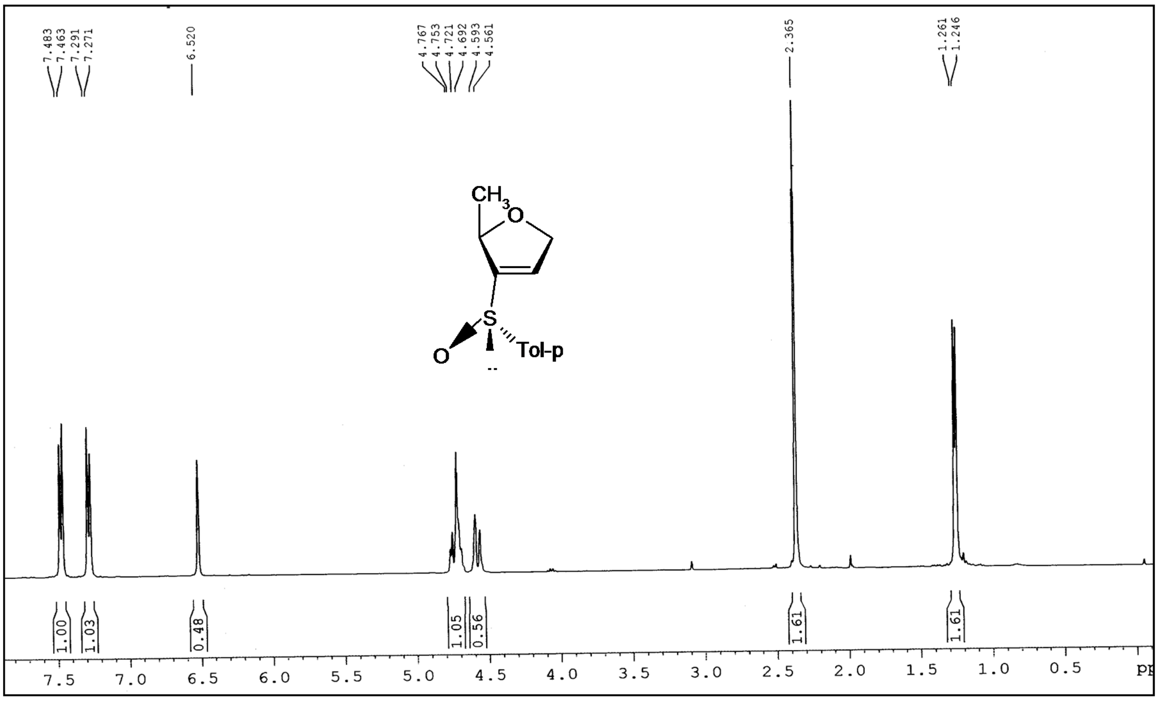

Figure S43. ^{1}H-NMR spectrum of compound **19S**s.

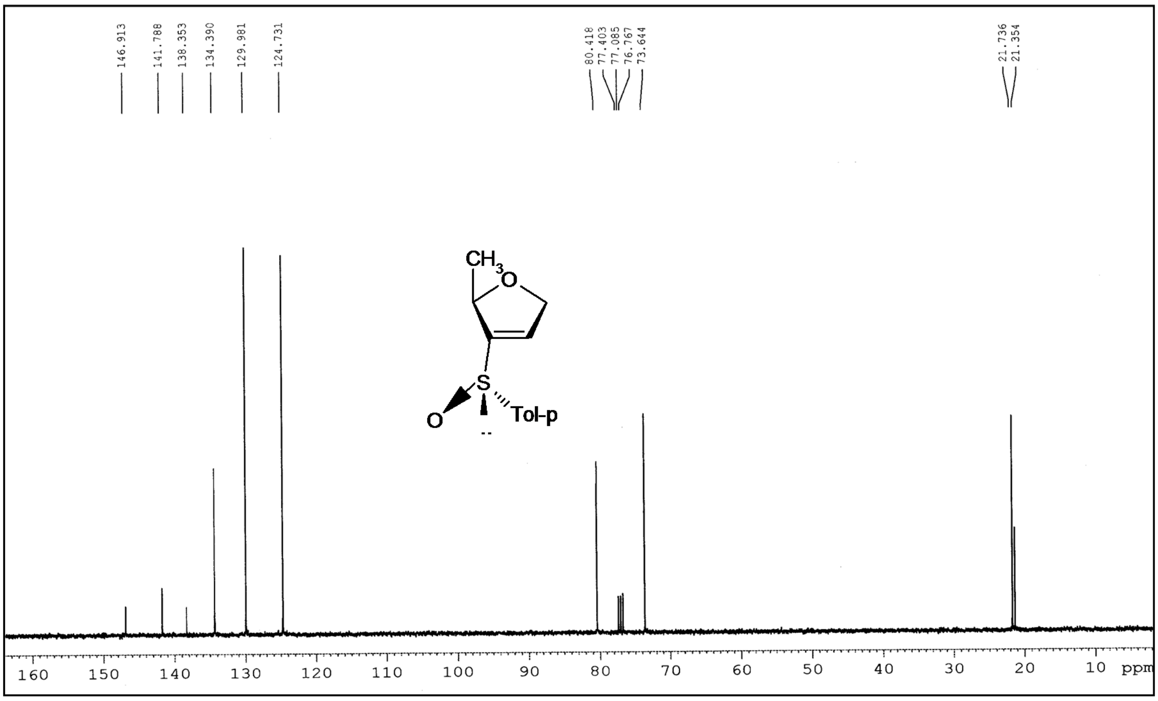

Figure S44. ^{13}C-NMR spectrum of compound **19S**s.

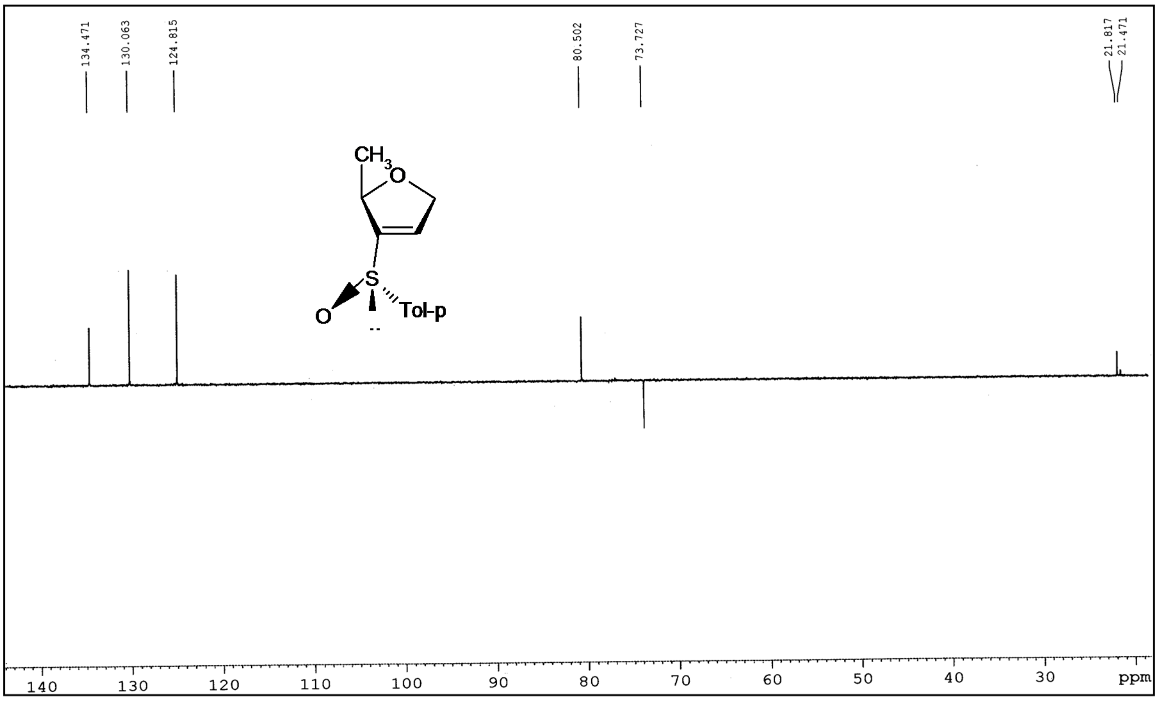

Figure S45. DEPT spectrum of compound **19S**s.

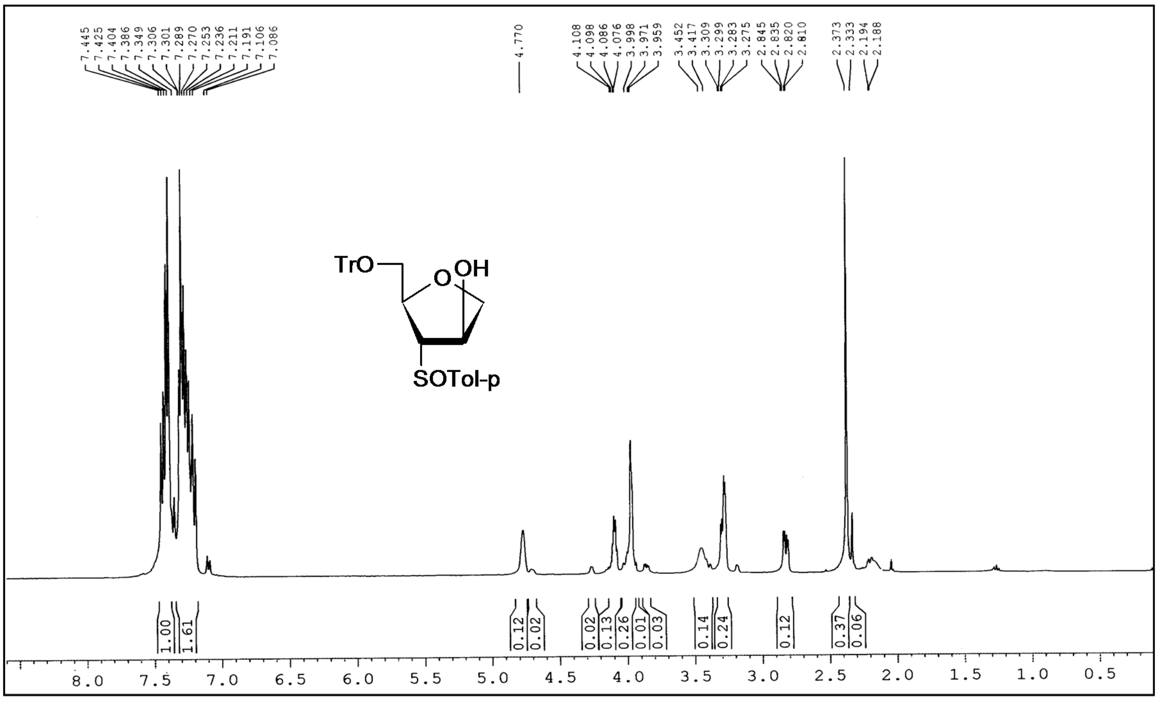

Figure S46. ^{1}H-NMR spectrum of compound **20.**

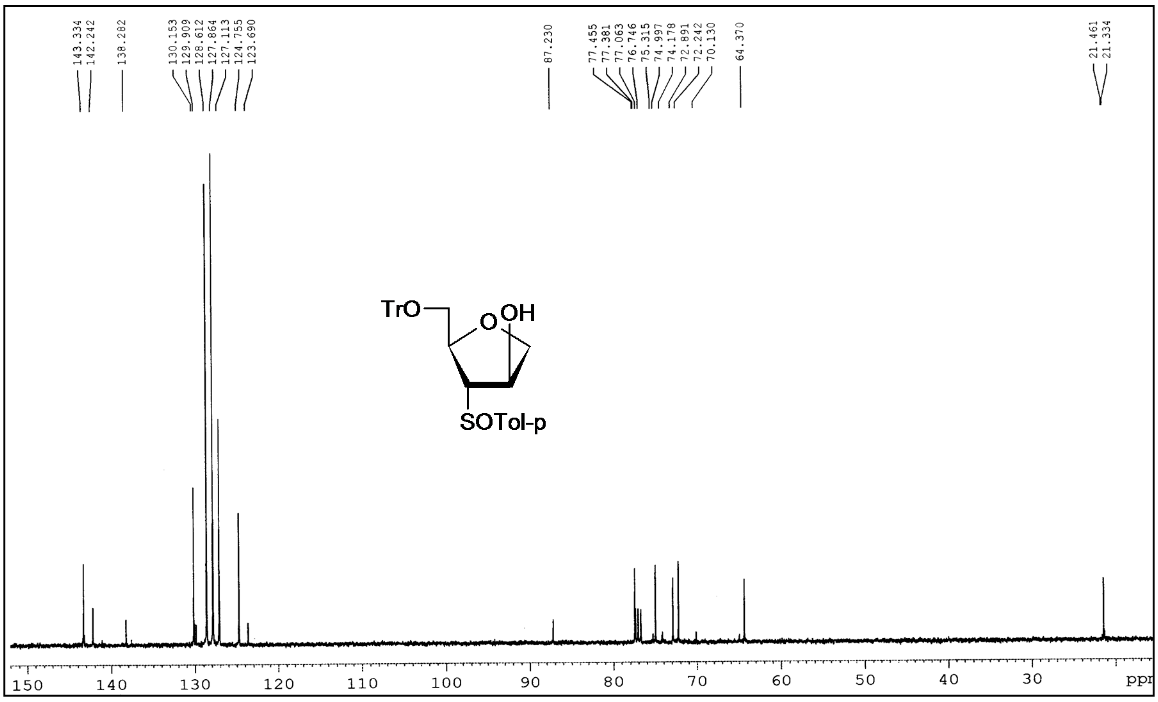

Figure S47. ^{13}C-NMR spectrum of compound **20.**

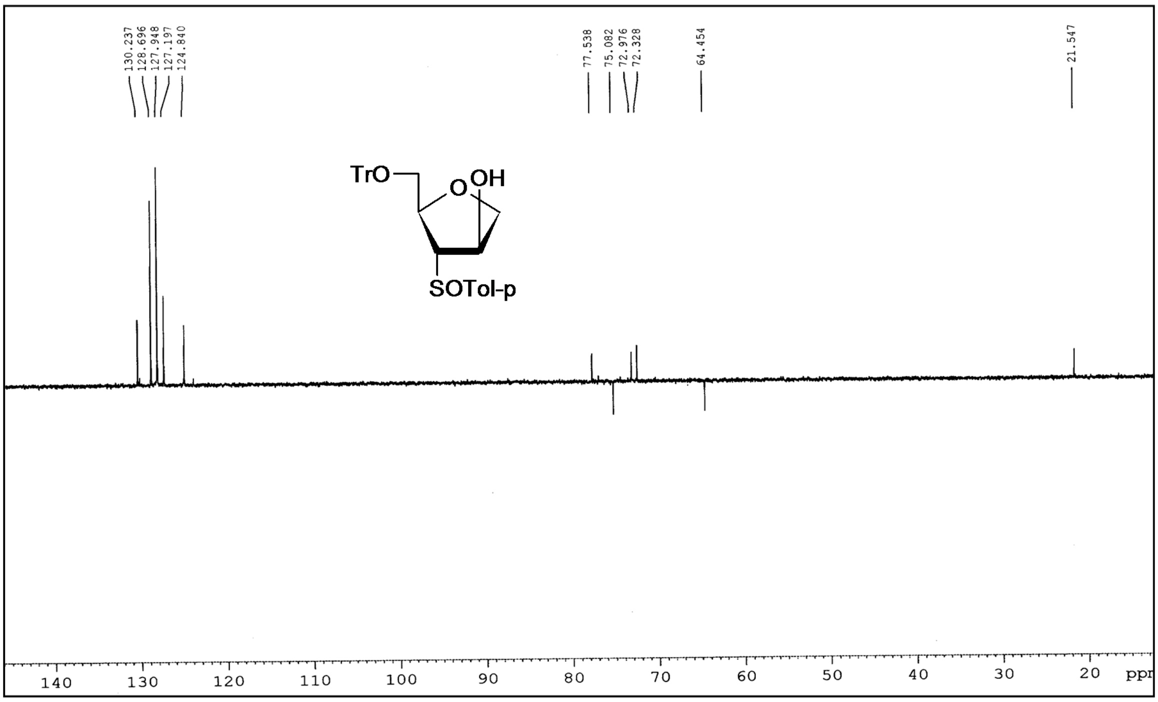

Figure S48. DEPT spectrum of compound **20.**

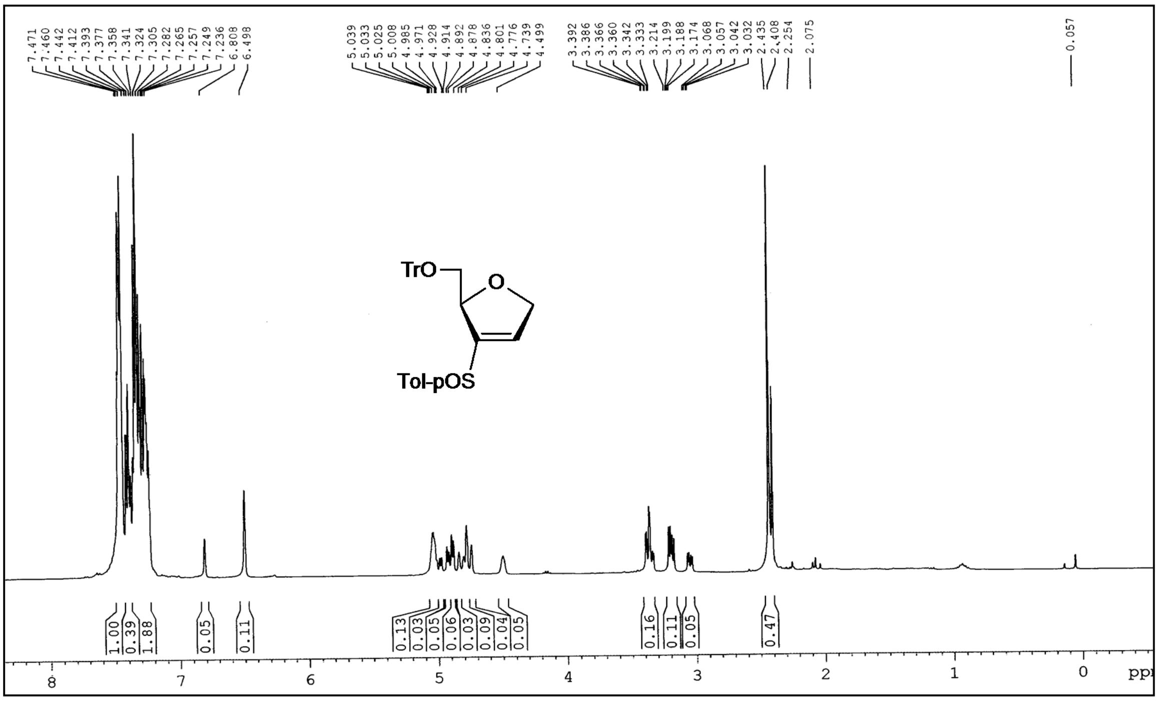

Figure S49. ^{1}H-NMR spectrum of compound **21.**

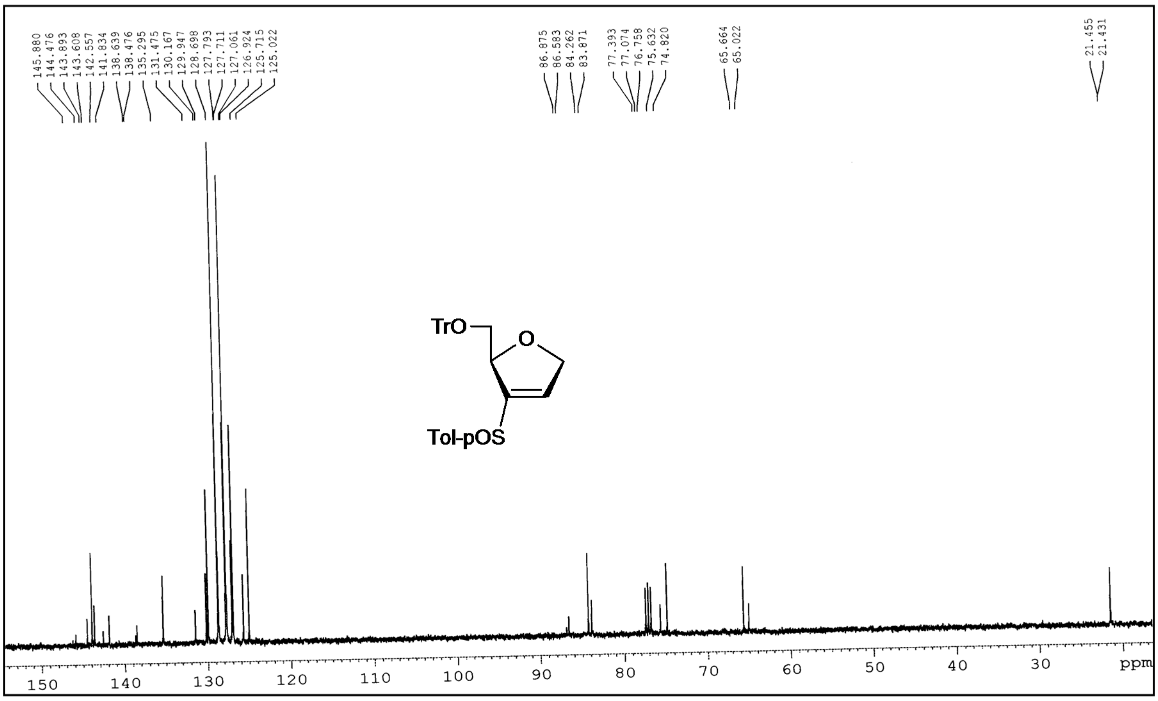

Figure S50. ^{13}C-NMR spectrum of compound **21.**

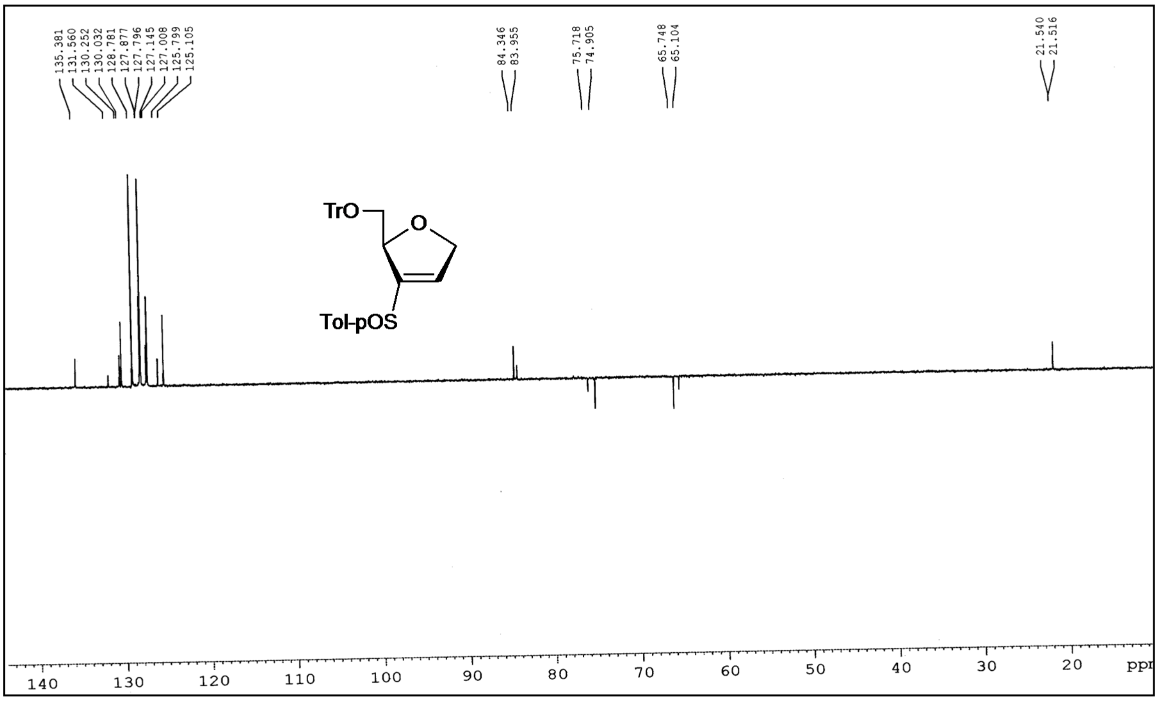

Figure S51. DEPT spectrum of compound **21.**

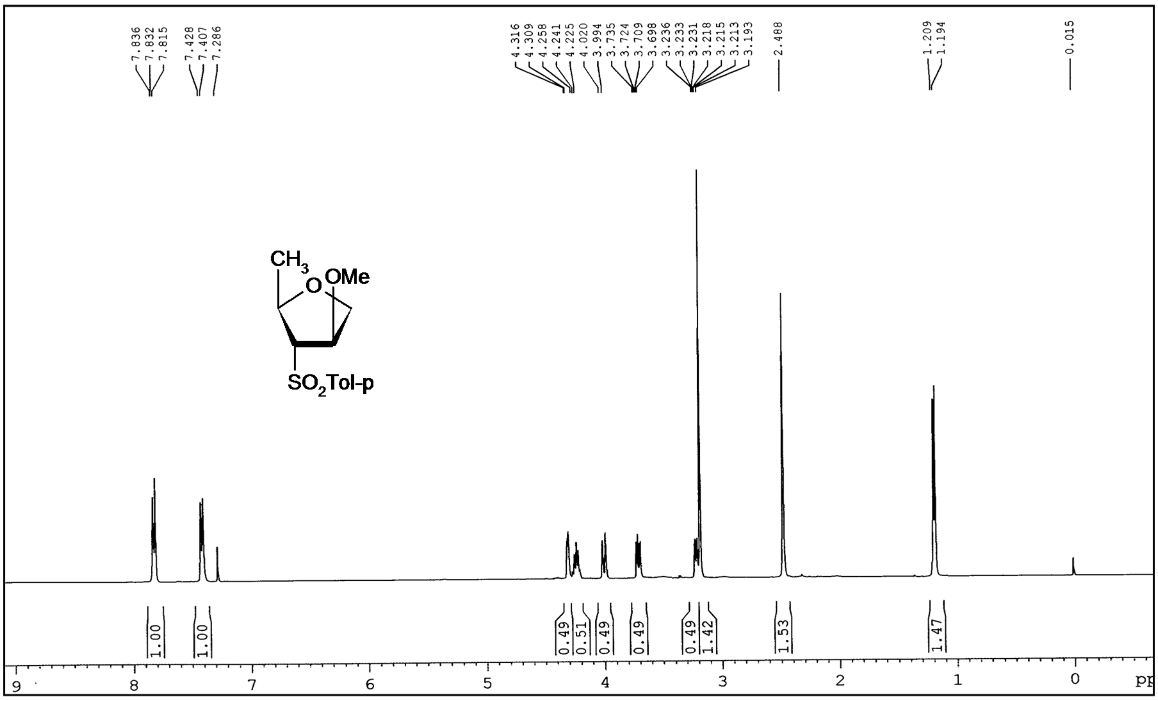

Figure S52. ^{1}H-NMR spectrum of compound **22.**

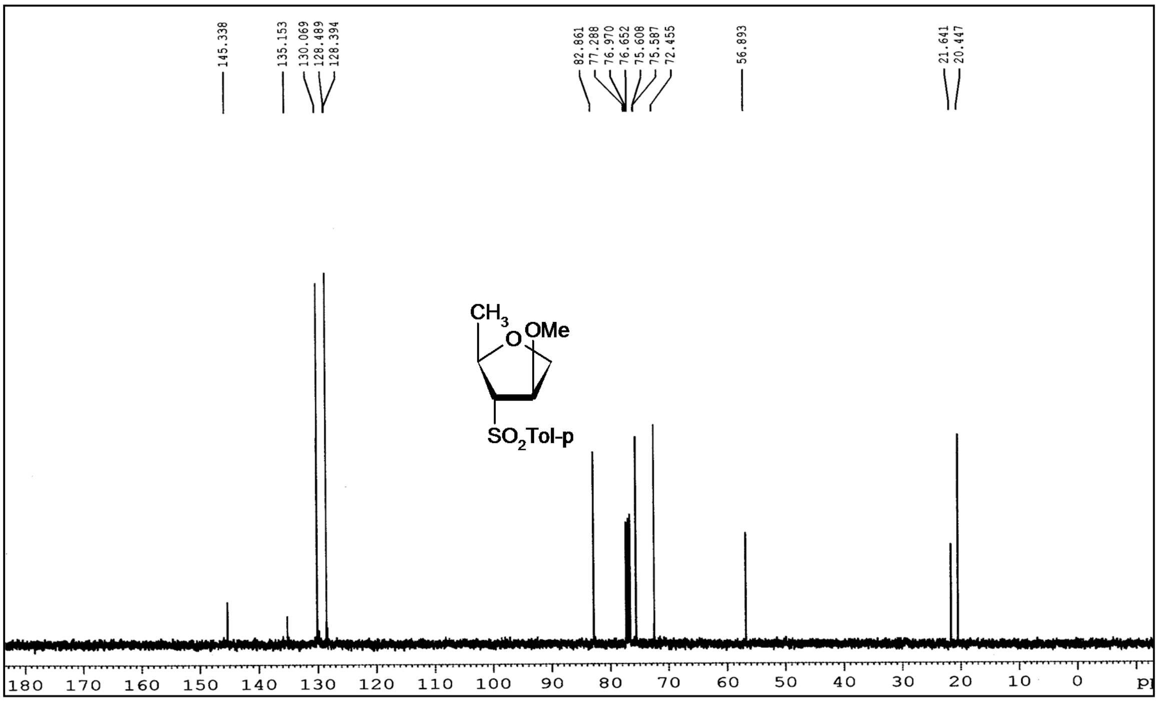

Figure S53. ^{13}C-NMR spectrum of compound **22.**

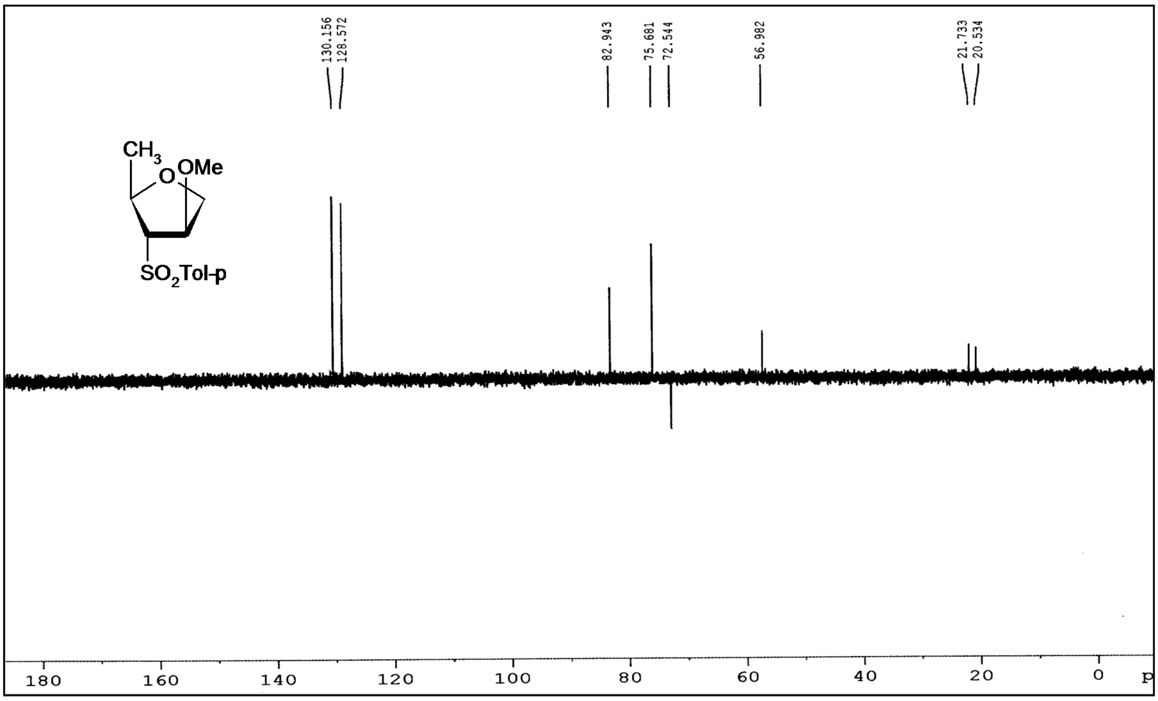

Figure S54. DEPT spectrum of compound **22.**

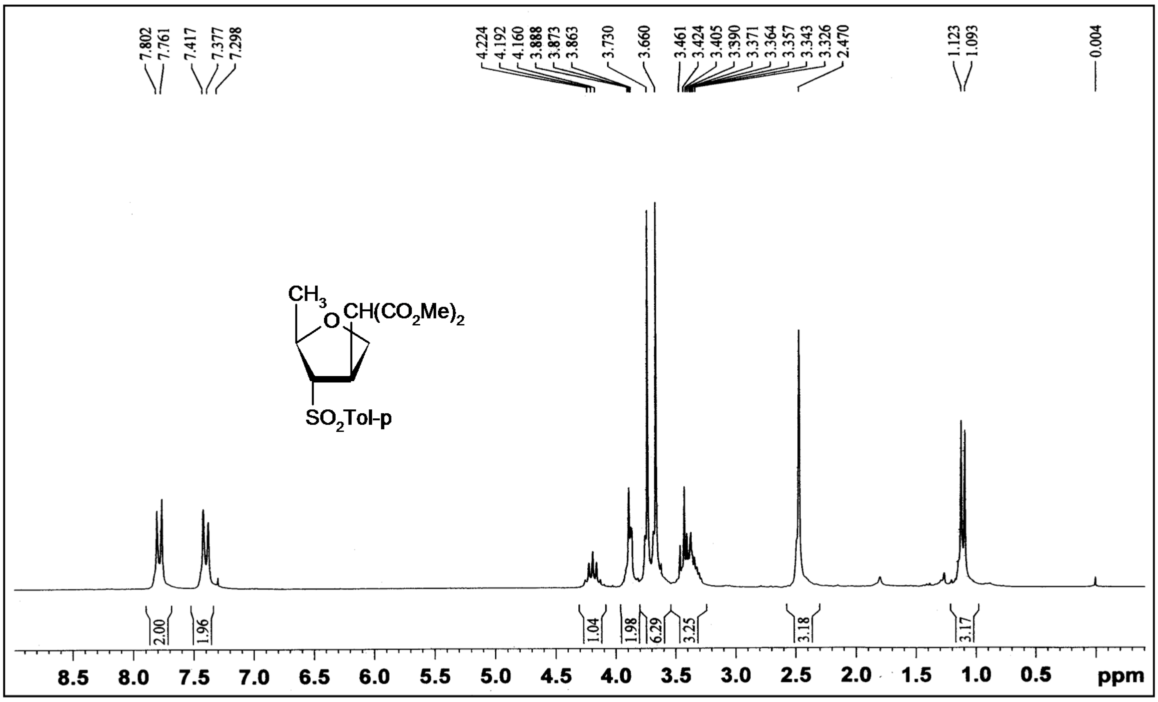

Figure S55. ^{1}H-NMR spectrum of compound **23.**

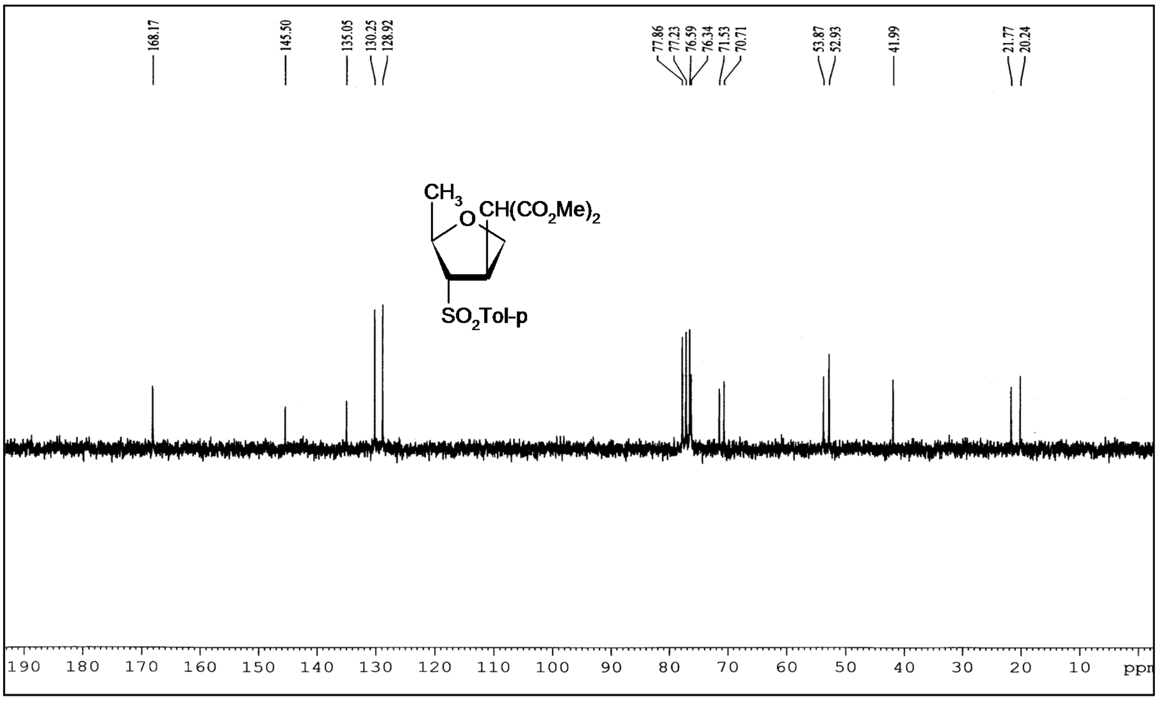

Figure S56. ^{13}C-NMR spectrum of compound **23.**

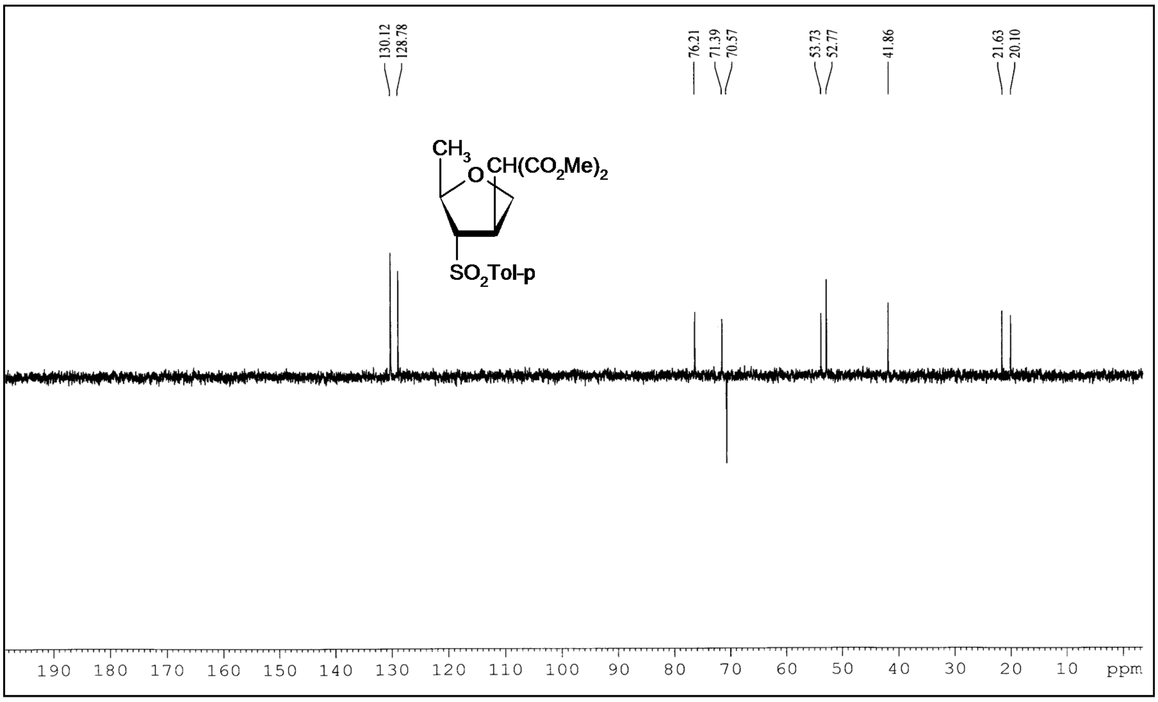

Figure S57. DEPT spectrum of compound **23.**

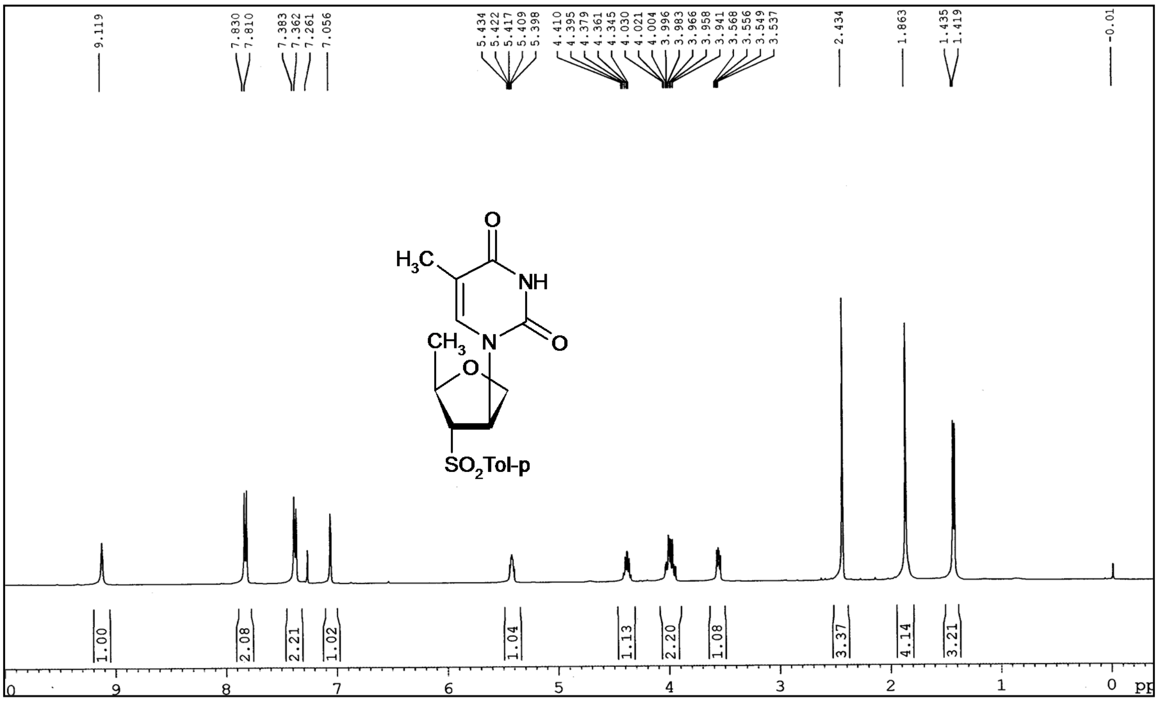

Figure S58. ^{1}H-NMR spectrum of compound **24.**

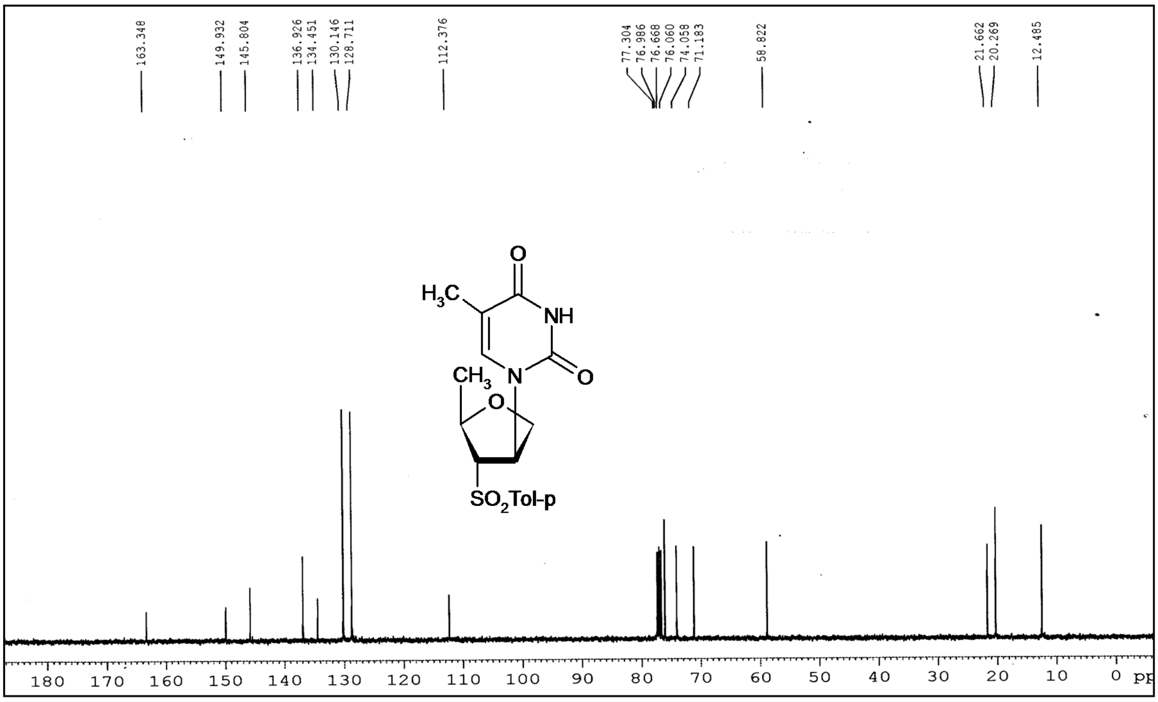

Figure S59. ^{13}C-NMR spectrum of compound **24.**

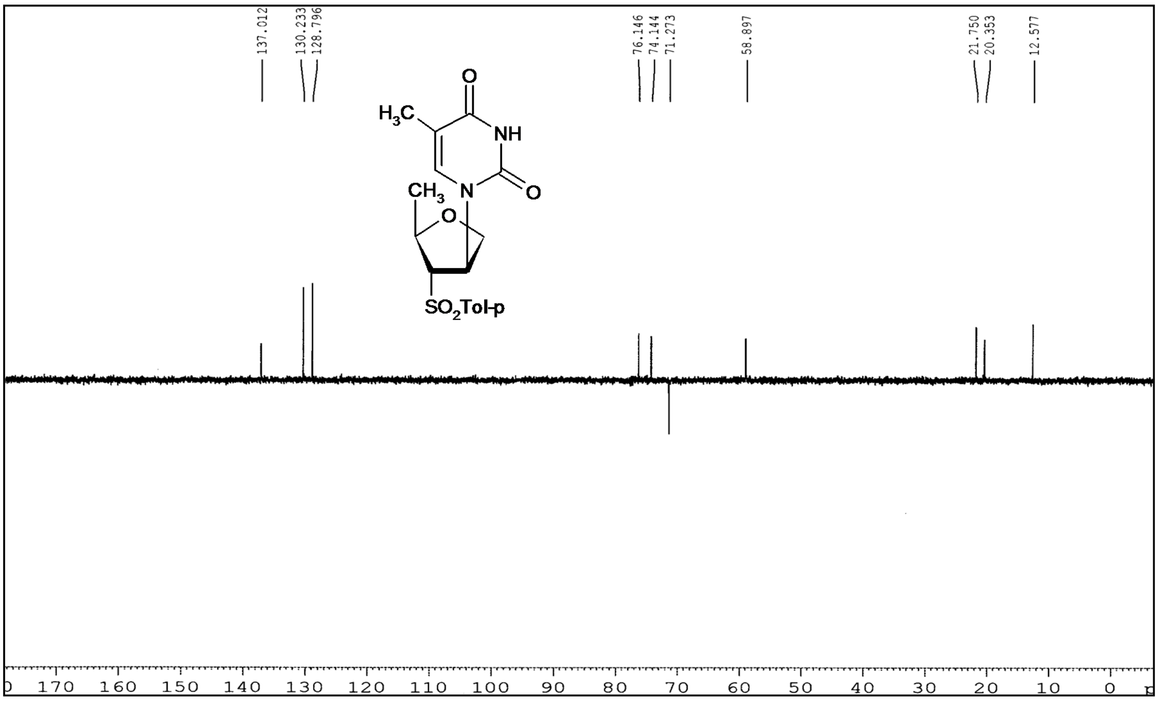

Figure S60. DEPT spectrum of compound **24.**

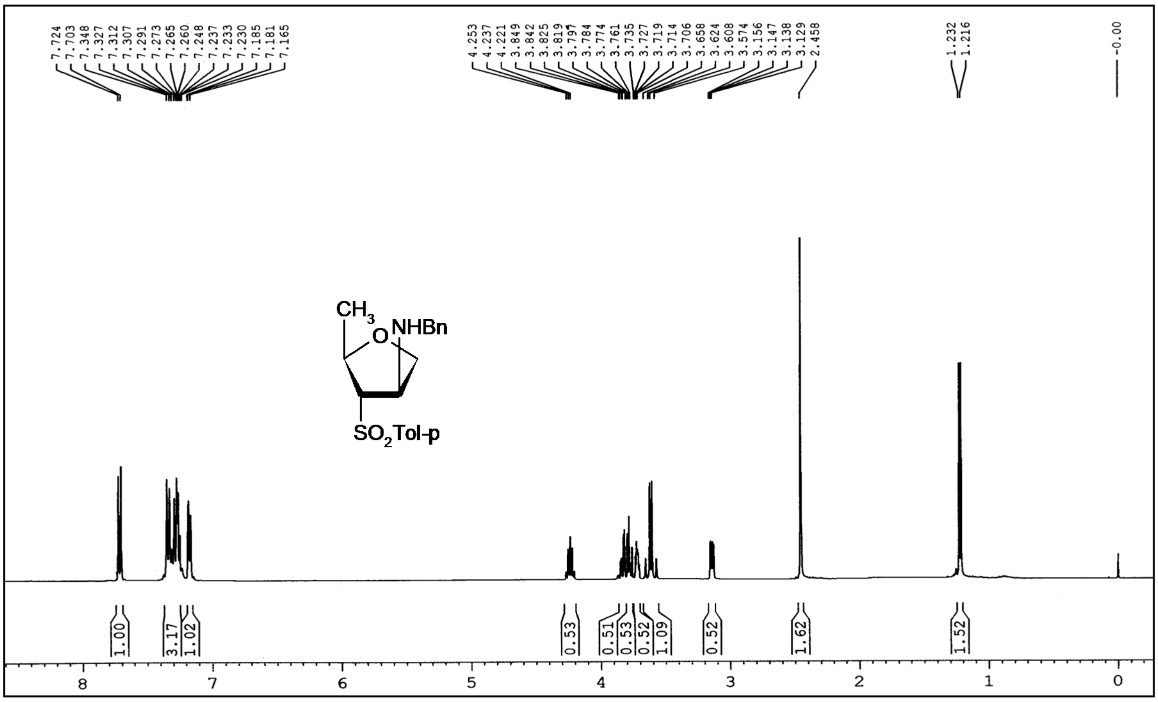

Figure S61. ^{1}H-NMR spectrum of compound **25.**

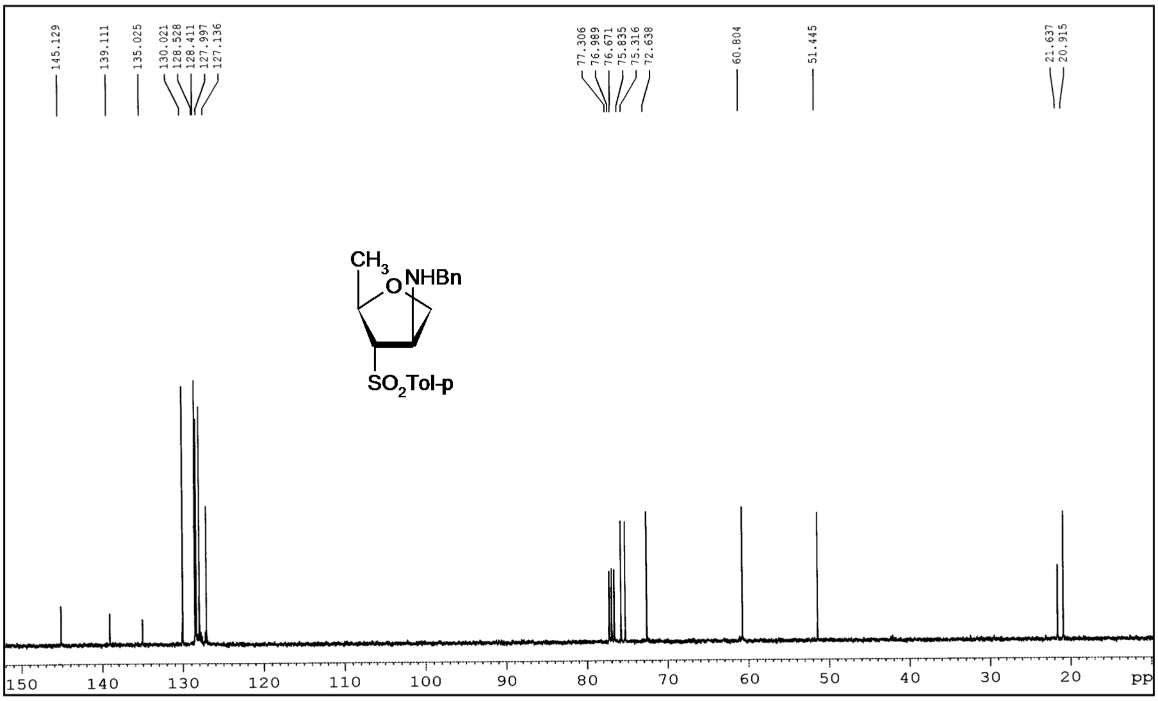

Figure S62. ^{13}C-NMR spectrum of compound **25.**

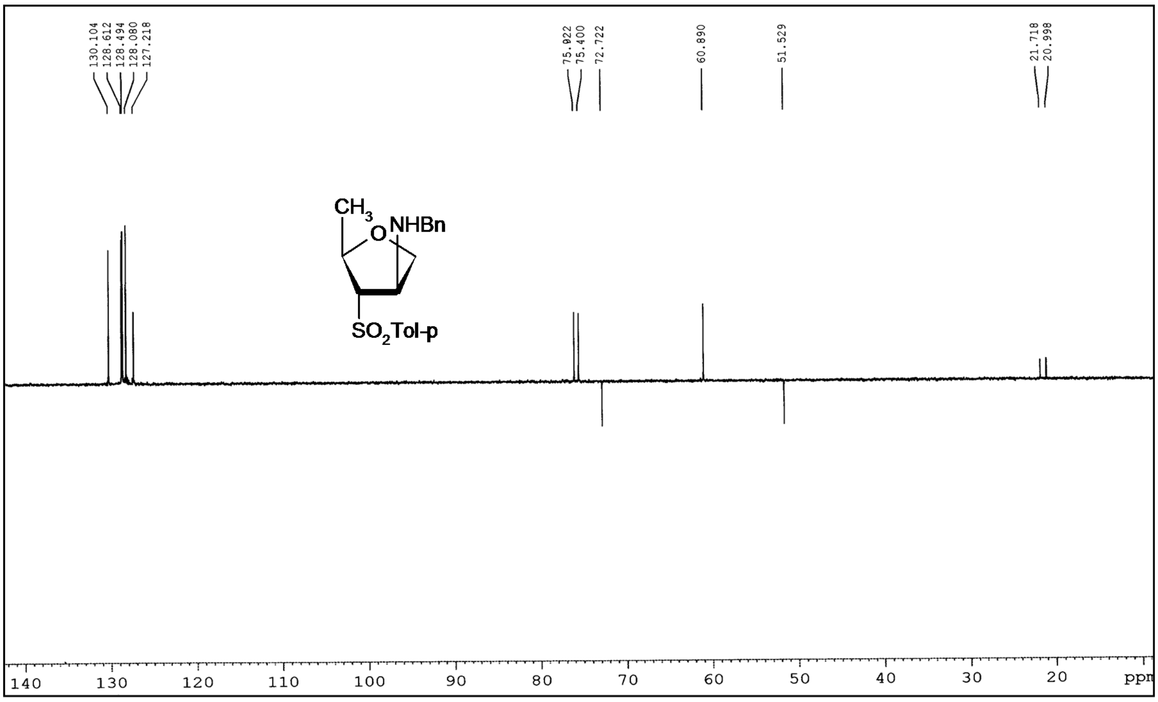

Figure S63. DEPT spectrum of compound **25.**

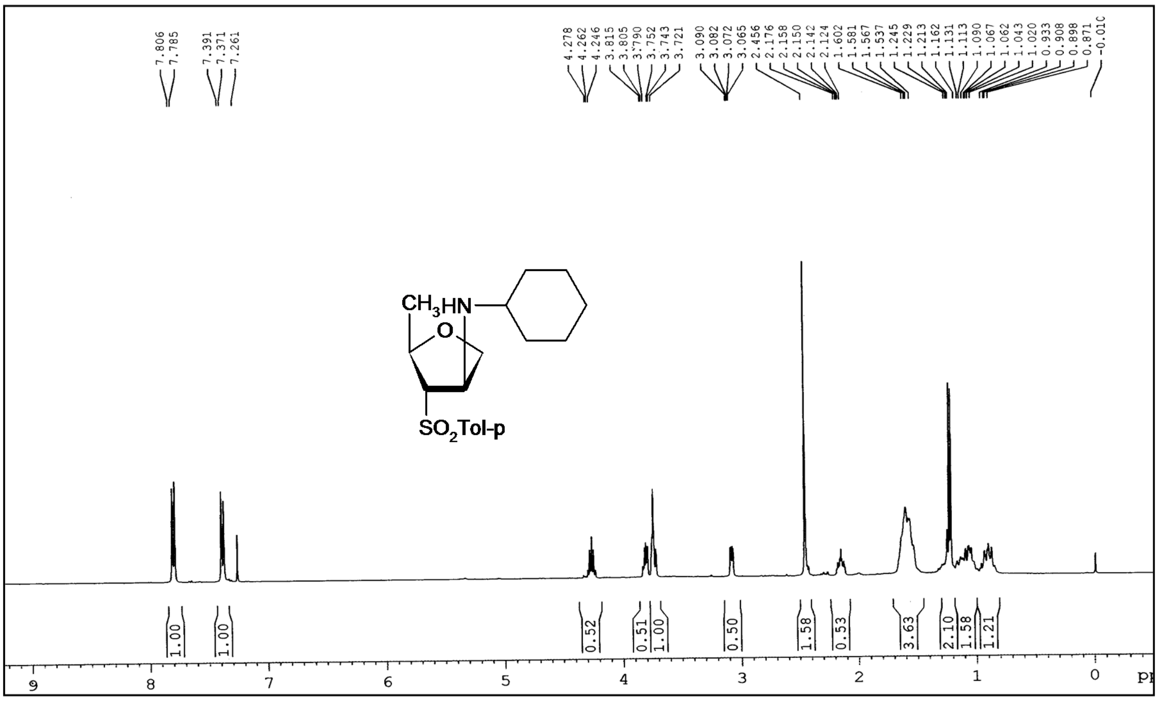

Figure S64. ^{1}H-NMR spectrum of compound **26.**

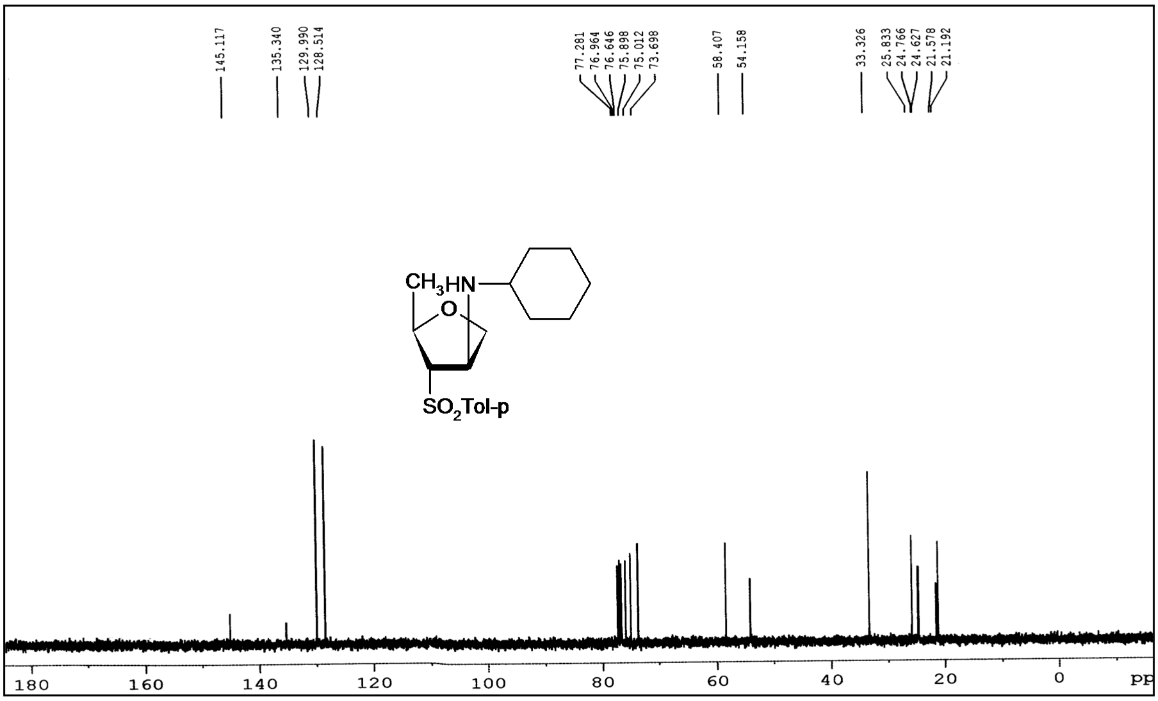

Figure S65. ^{13}C-NMR spectrum of compound **26.**

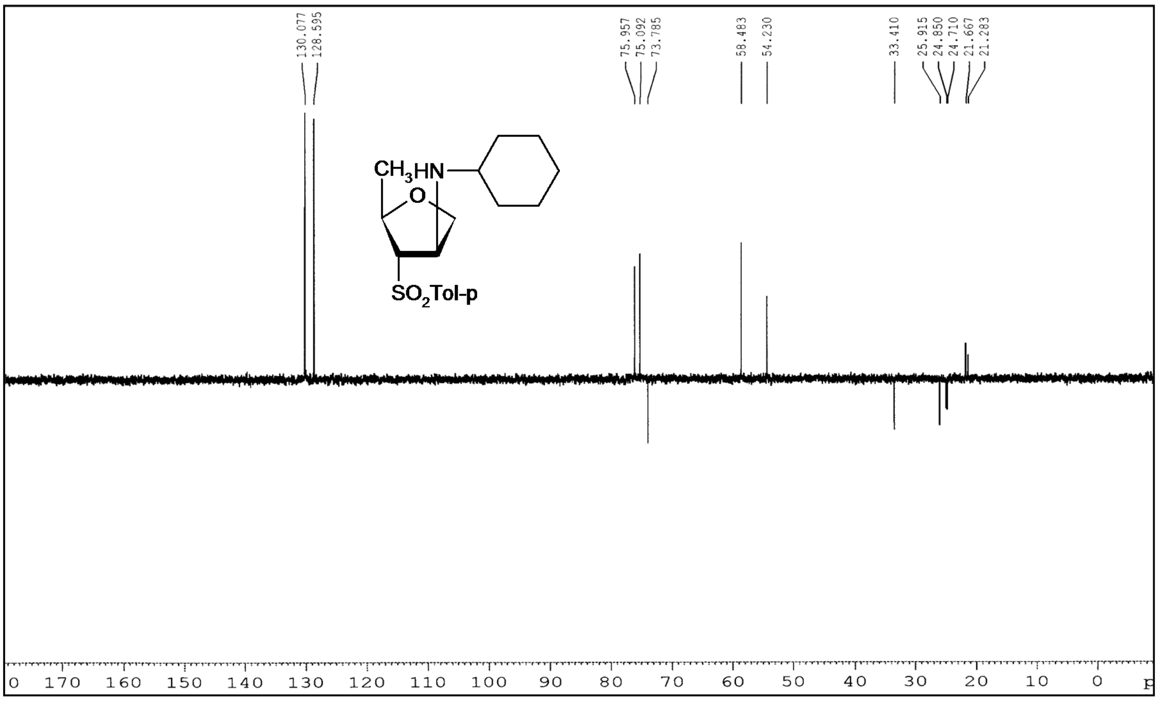

Figure S66. DEPT spectrum of compound **26.**

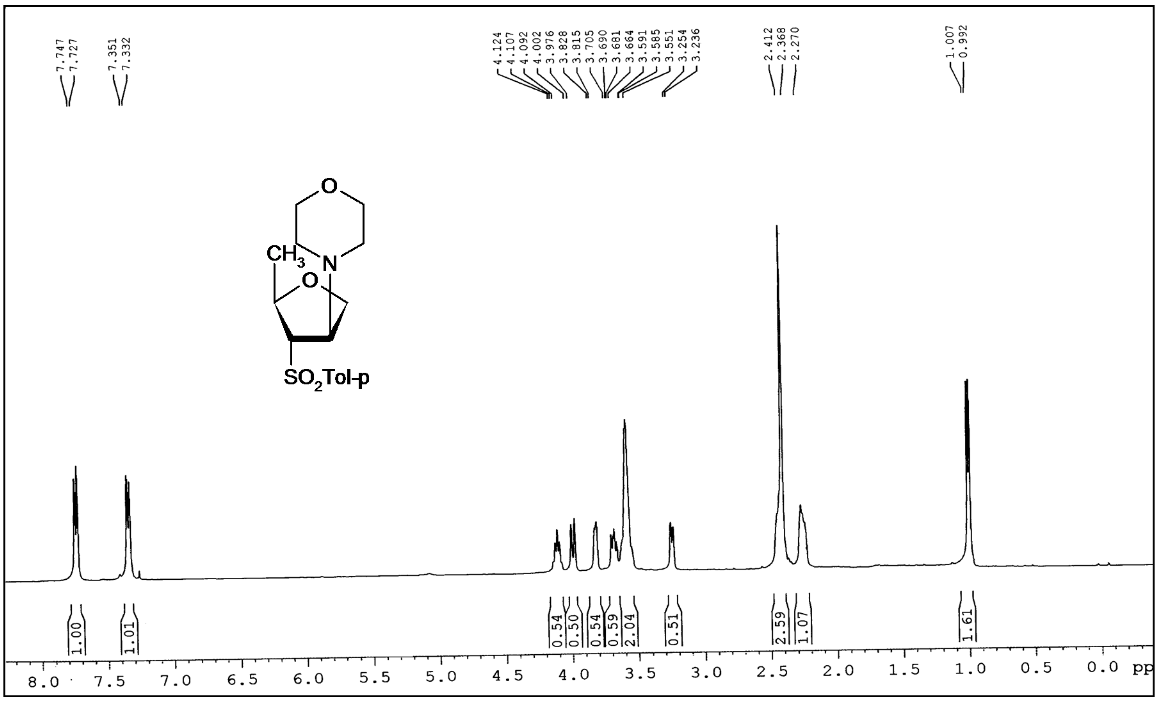

Figure S67. ^{1}H-NMR spectrum of compound **27.**

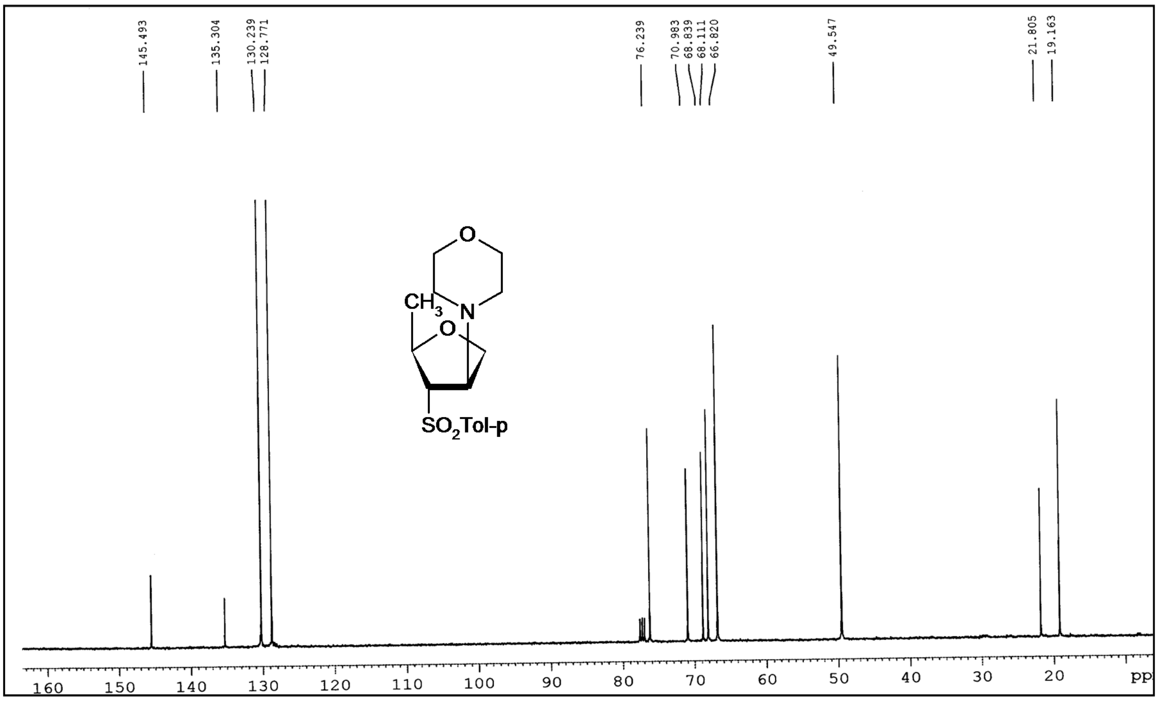

Figure S68. ^{13}C-NMR spectrum of compound **27.**

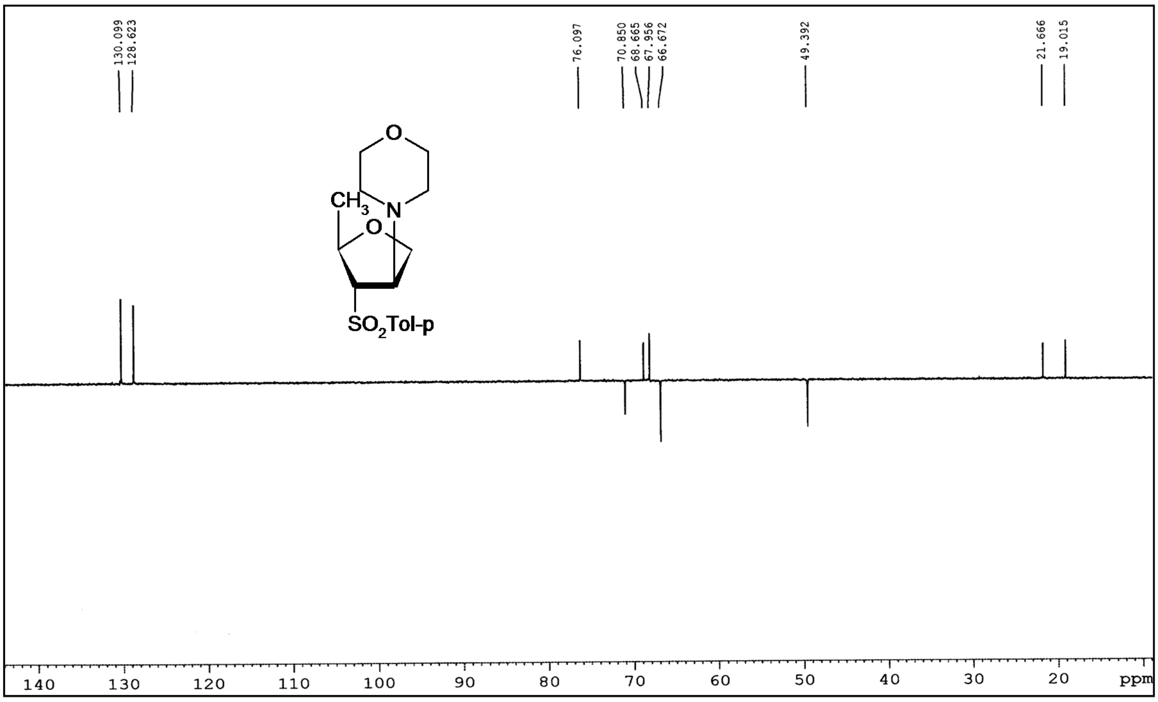

Figure S69. DEPT spectrum of compound **27.**

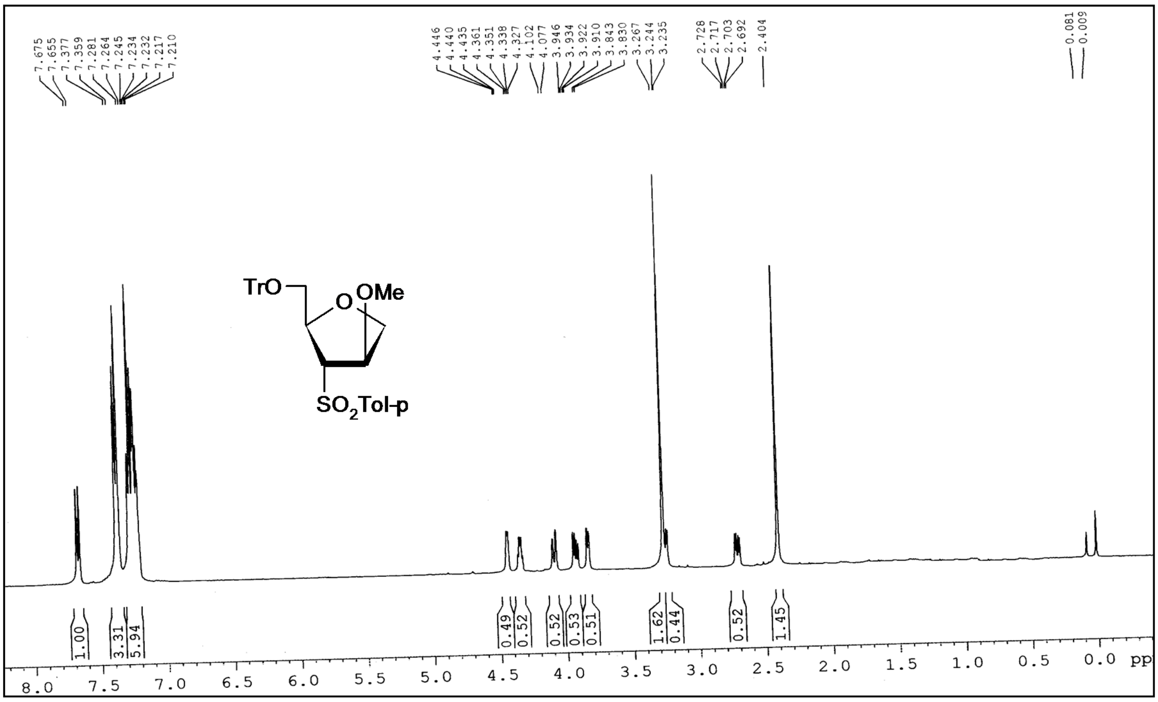

Figure S70. ^{1}H-NMR spectrum of compound **28.**

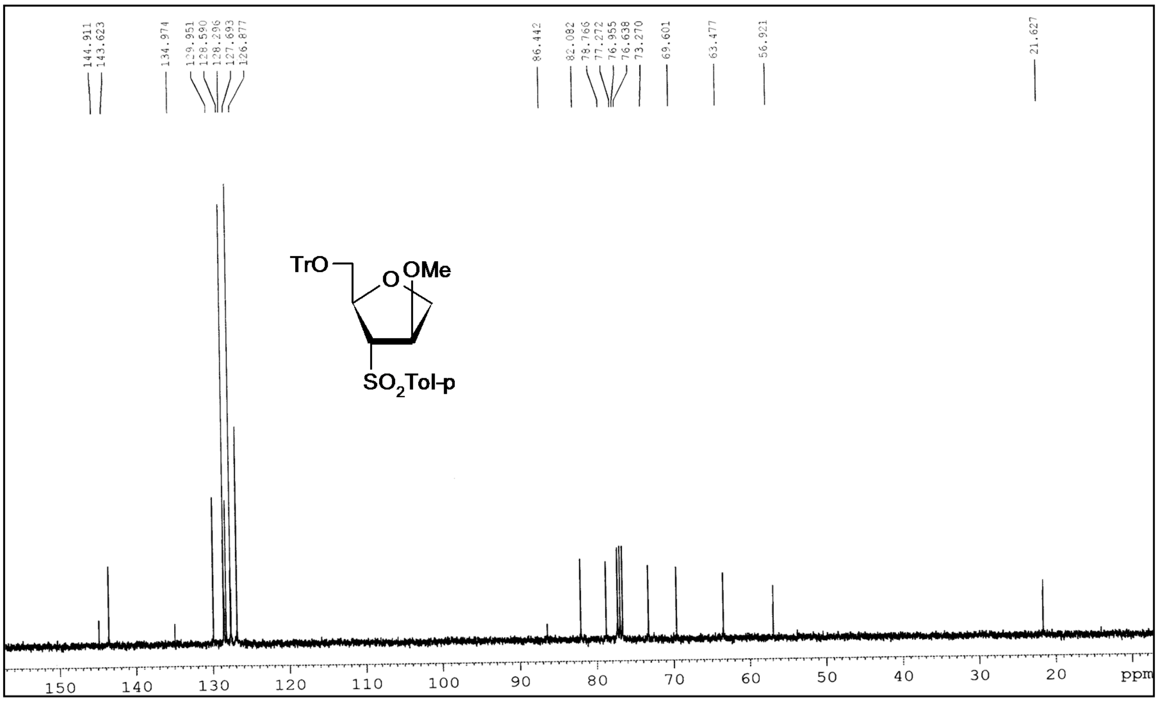

Figure S71. ^{13}C-NMR spectrum of compound **28.**

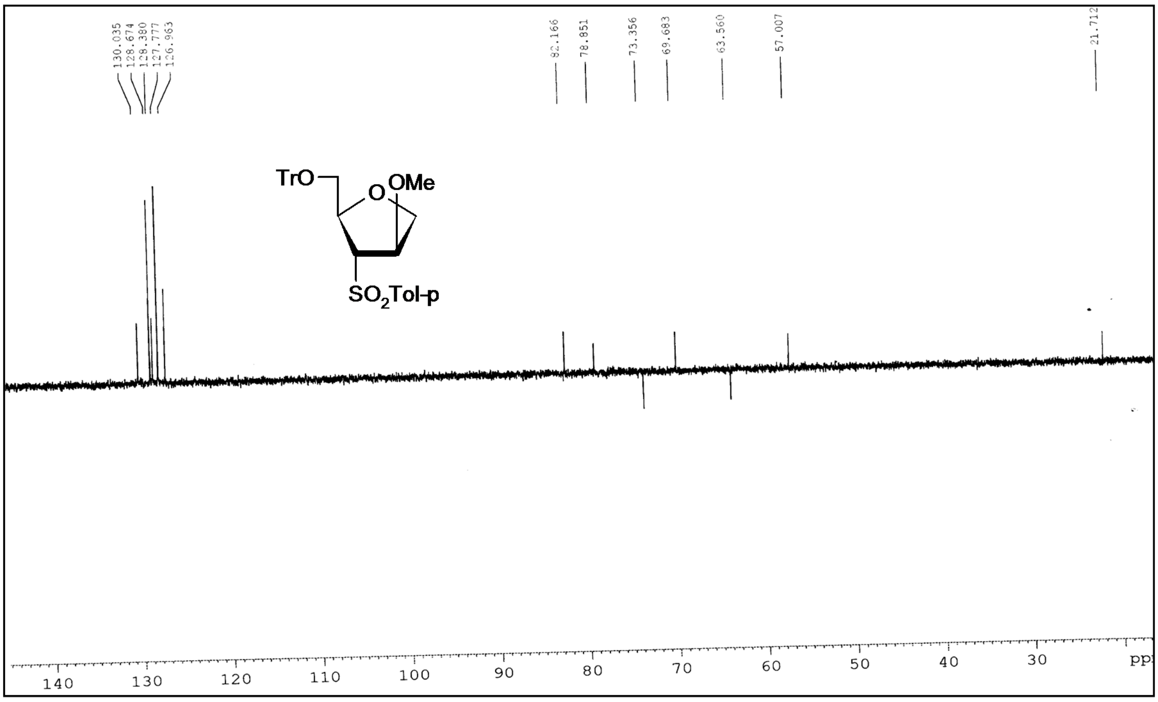

Figure S72. DEPT spectrum of compound **28.**

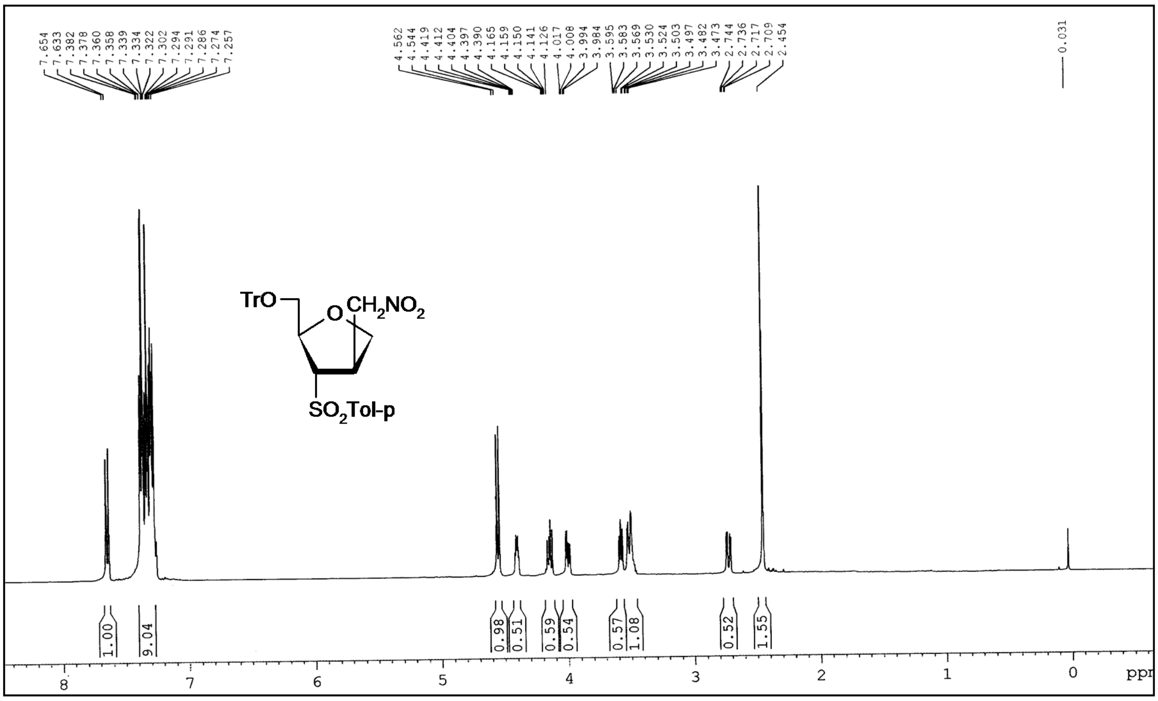

Figure S73. ^{1}H-NMR spectrum of compound **29.**

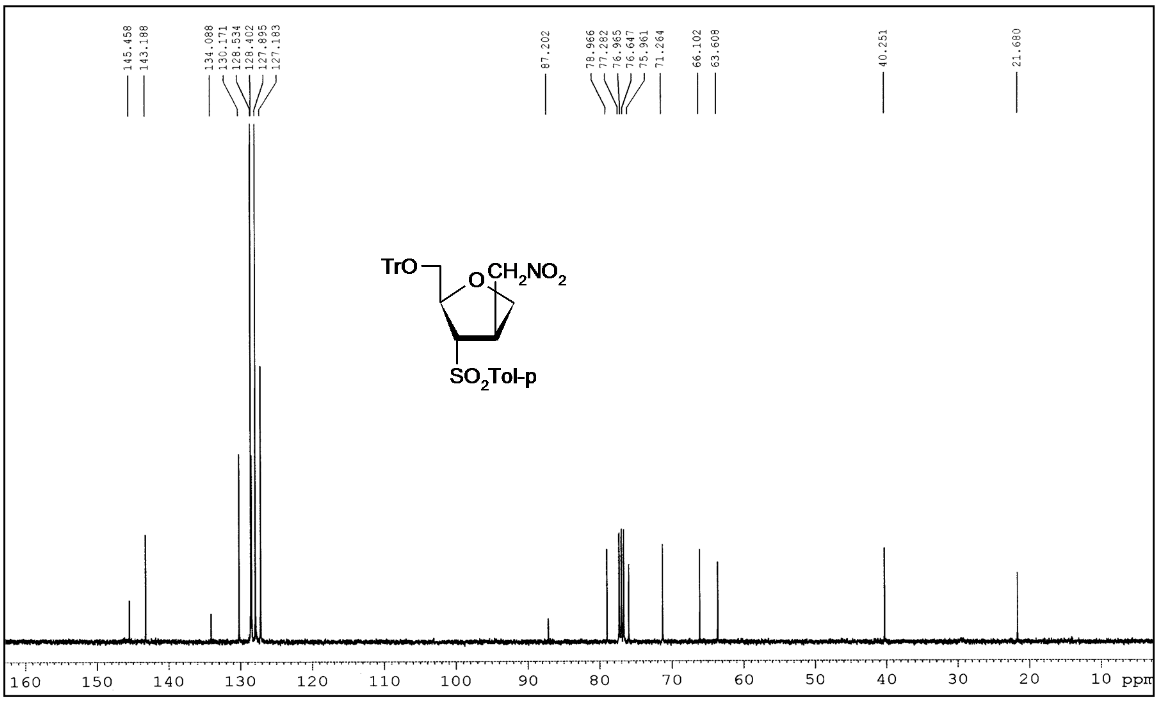

Figure S74. ^{13}C-NMR spectrum of compound **29.**

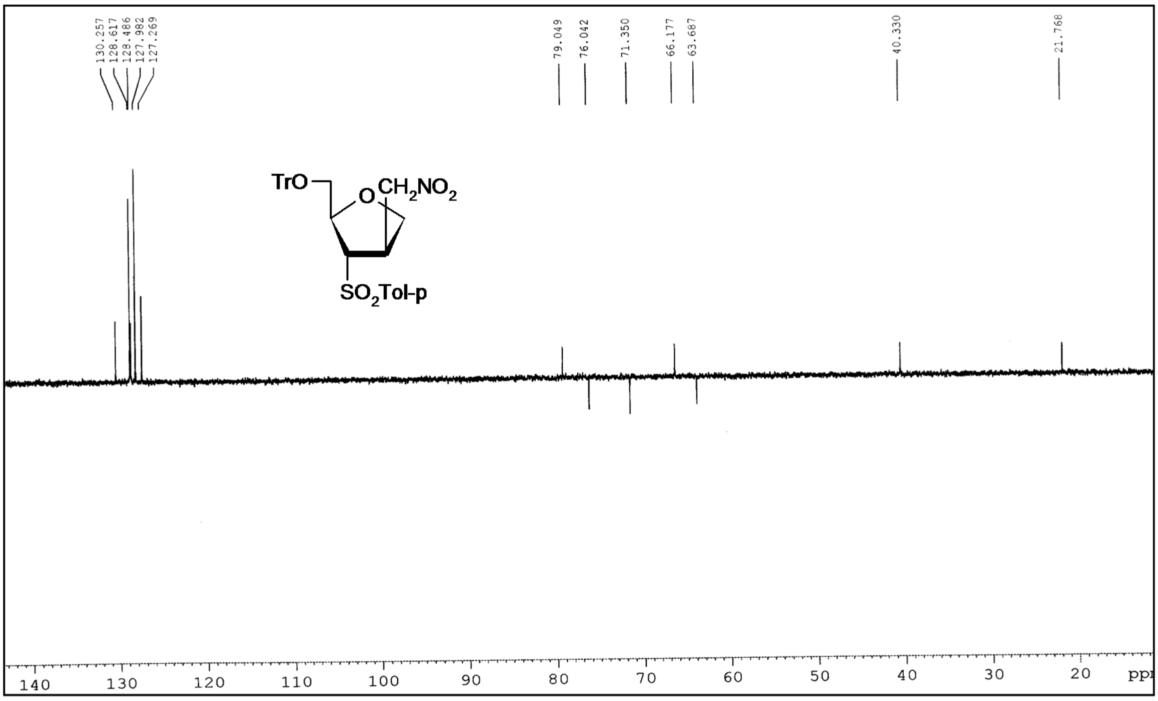

Figure S75. DEPT spectrum of compound **29.**

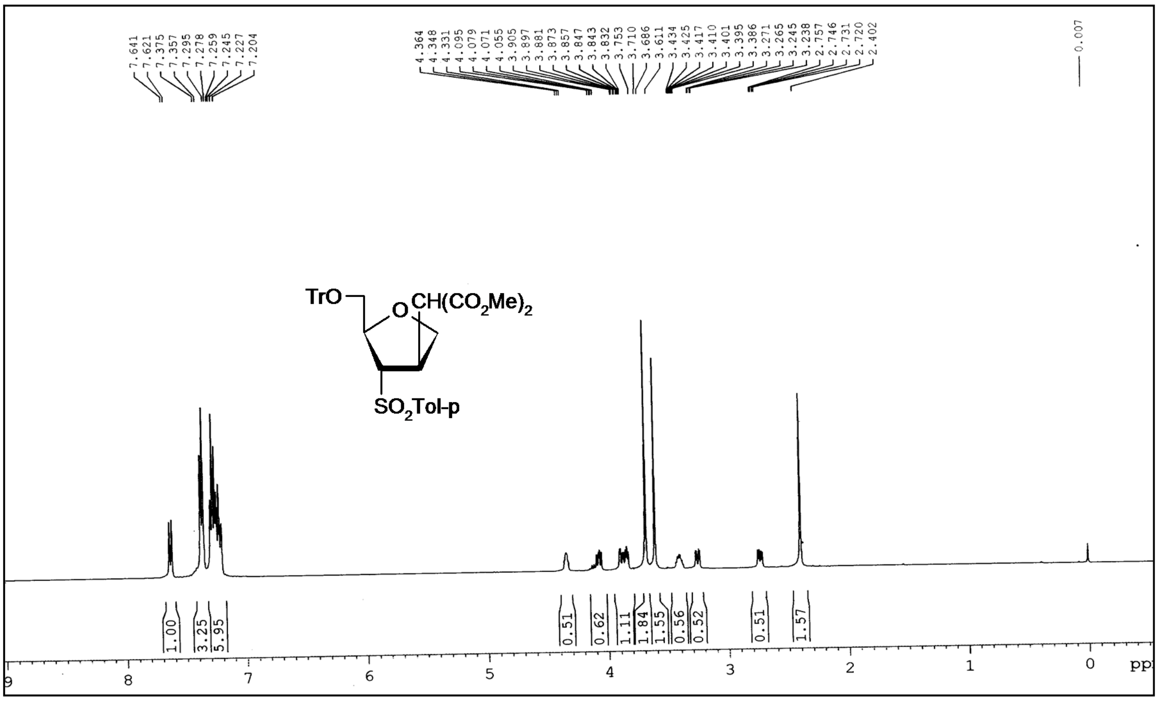

Figure S76. ^{1}H-NMR spectrum of compound **30.**

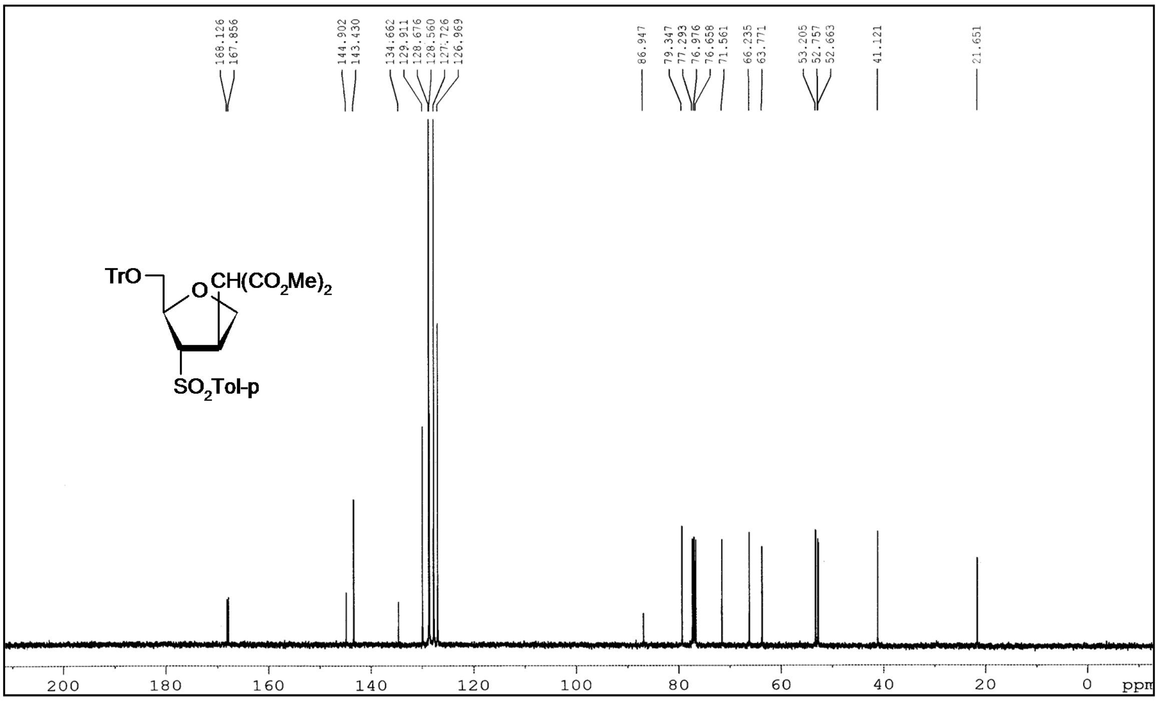

Figure S77. ^{13}C-NMR spectrum of compound **30.**

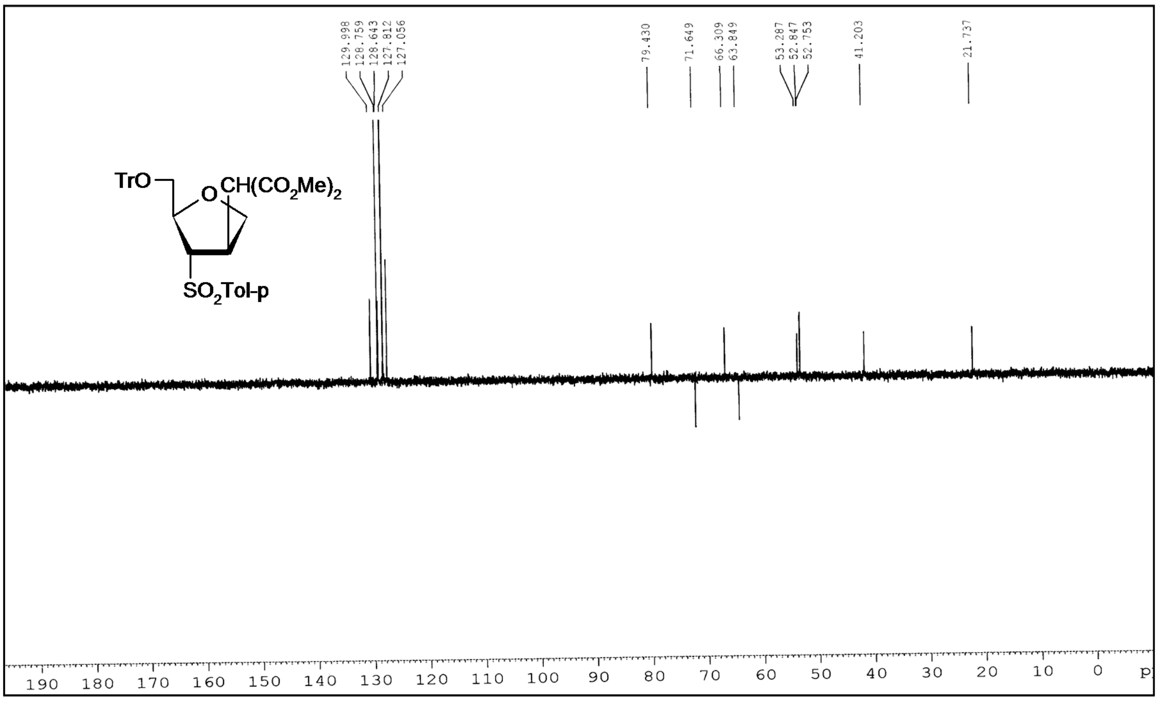

Figure S78. DEPT spectrum of compound **30.**

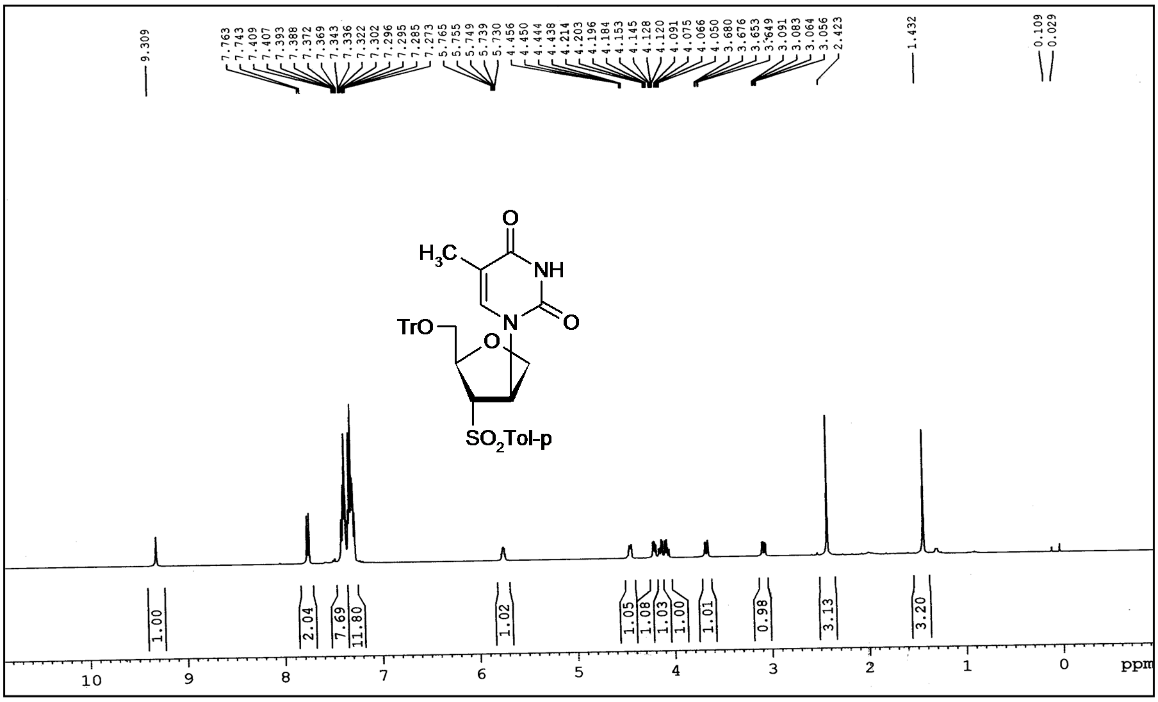

Figure S79. ^{1}H-NMR spectrum of compound **31.**

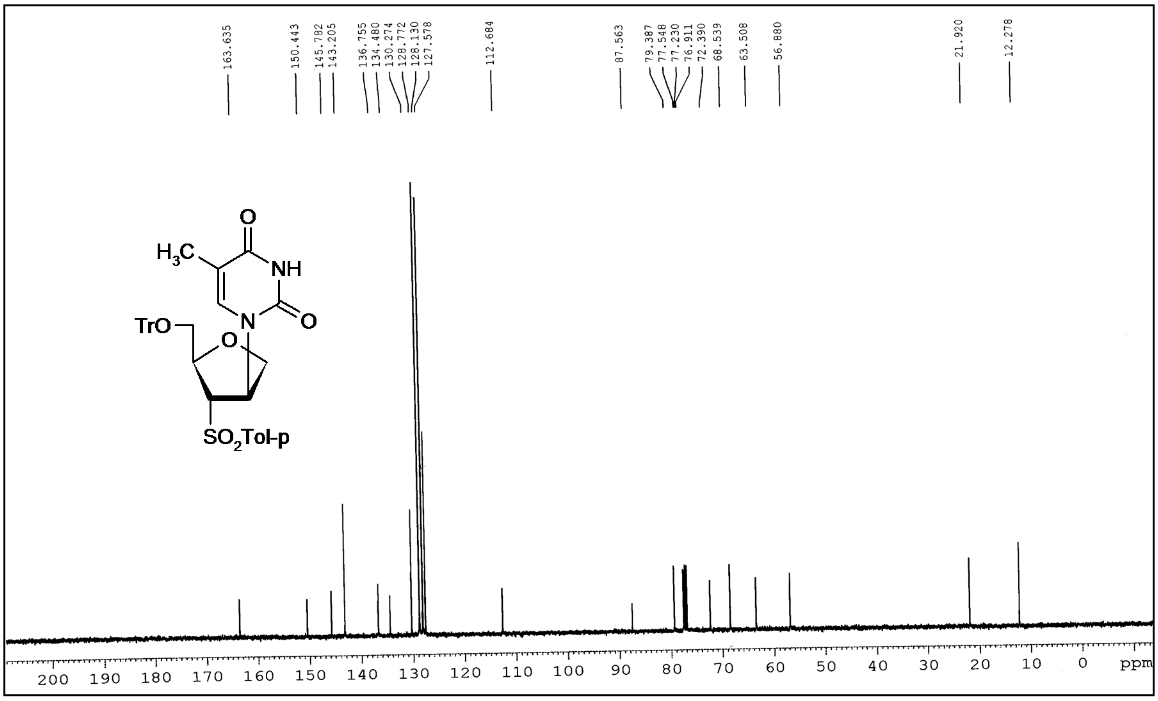

Figure S80. ^{13}C-NMR spectrum of compound **31.**

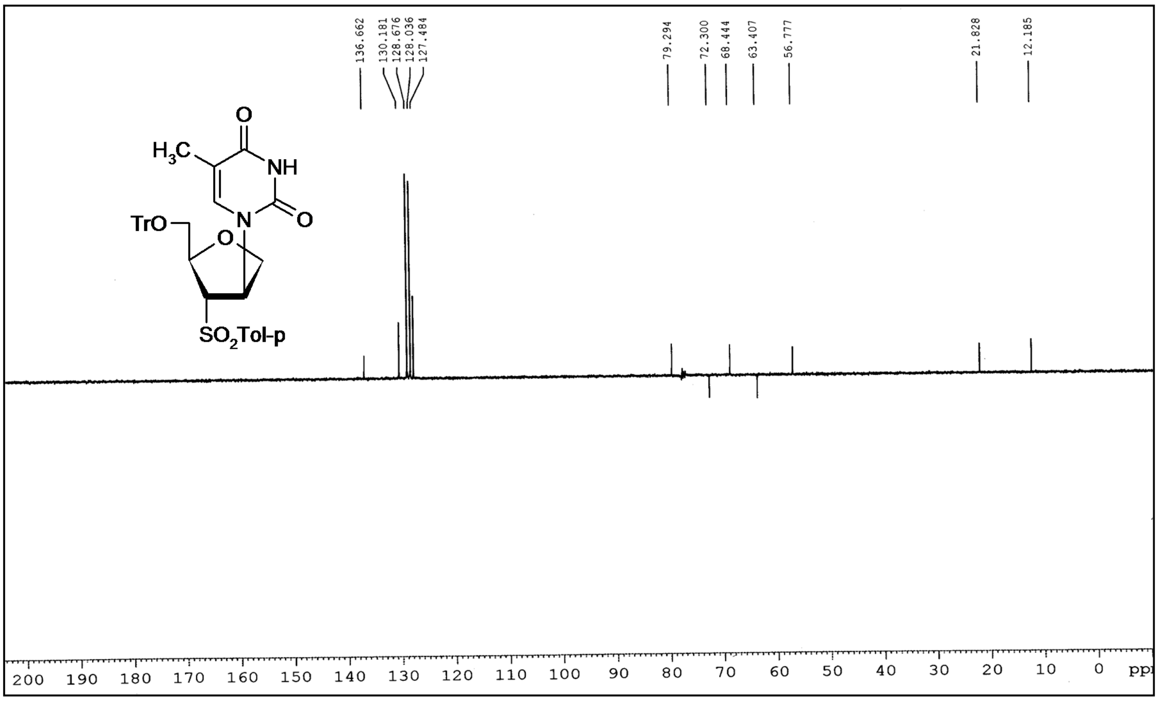

Figure S81. DEPT spectrum of compound **31.**

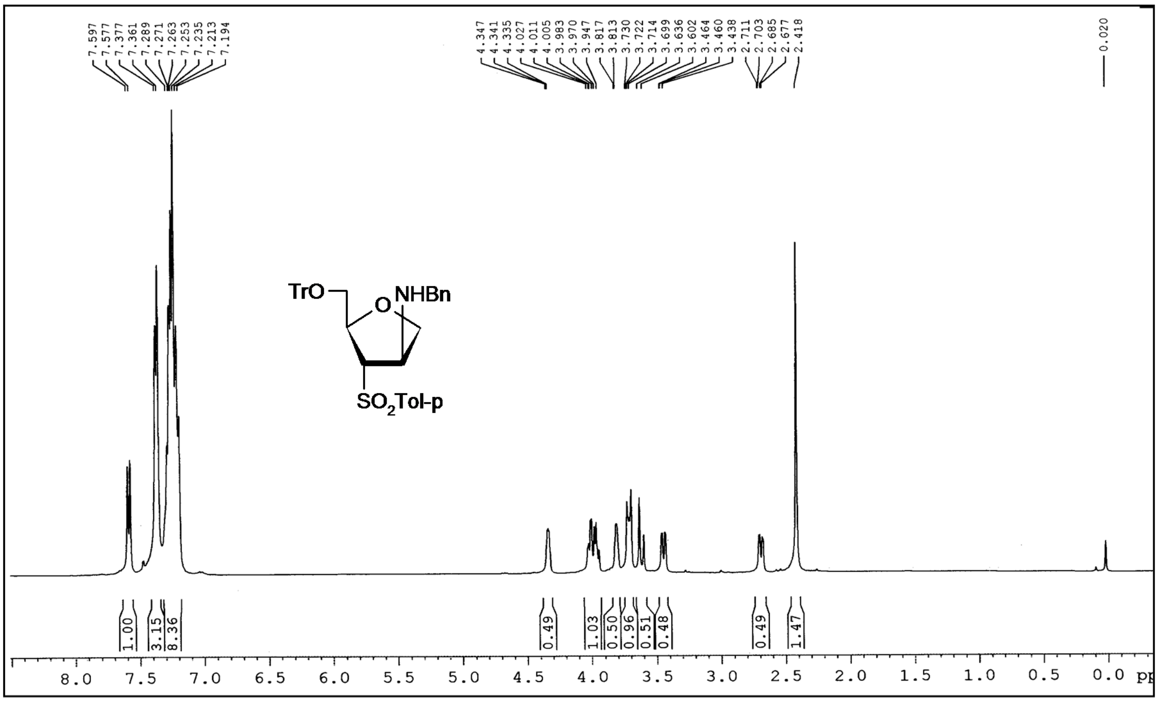

Figure S82. ^{1}H-NMR spectrum of compound **32.**

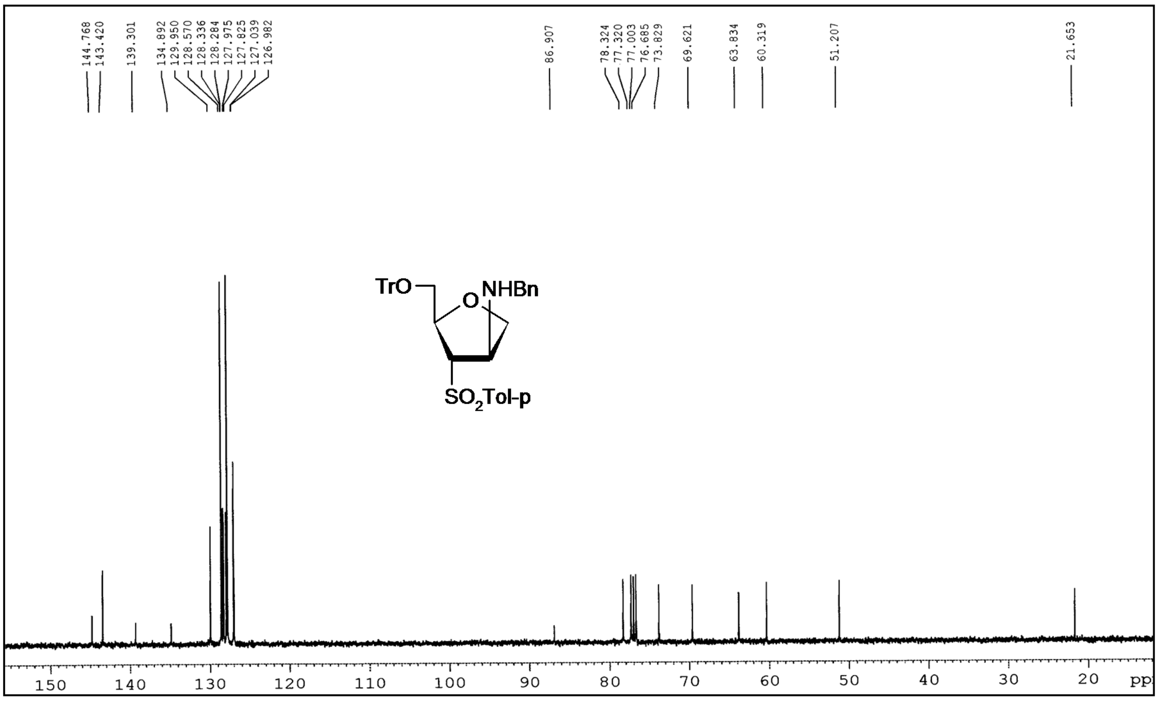

Figure S83. ^{13}C-NMR spectrum of compound **32.**

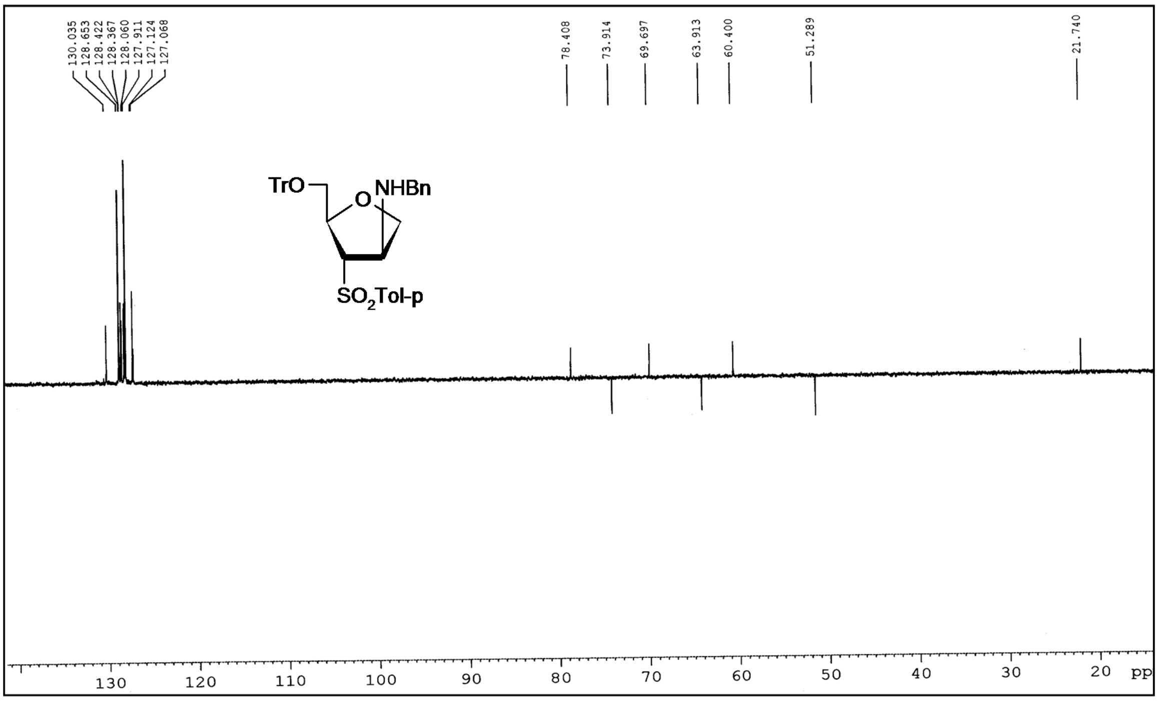

Figure S84. DEPT spectrum of compound **32.**

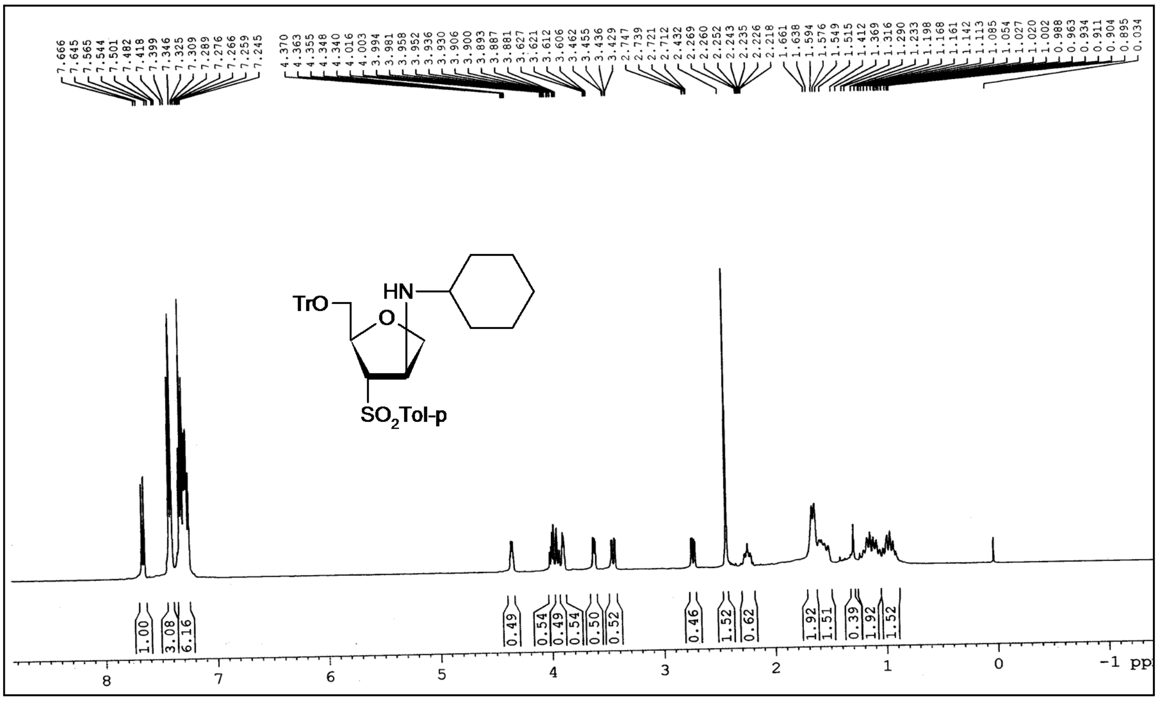

Figure S85. ^{1}H-NMR spectrum of compound **33.**

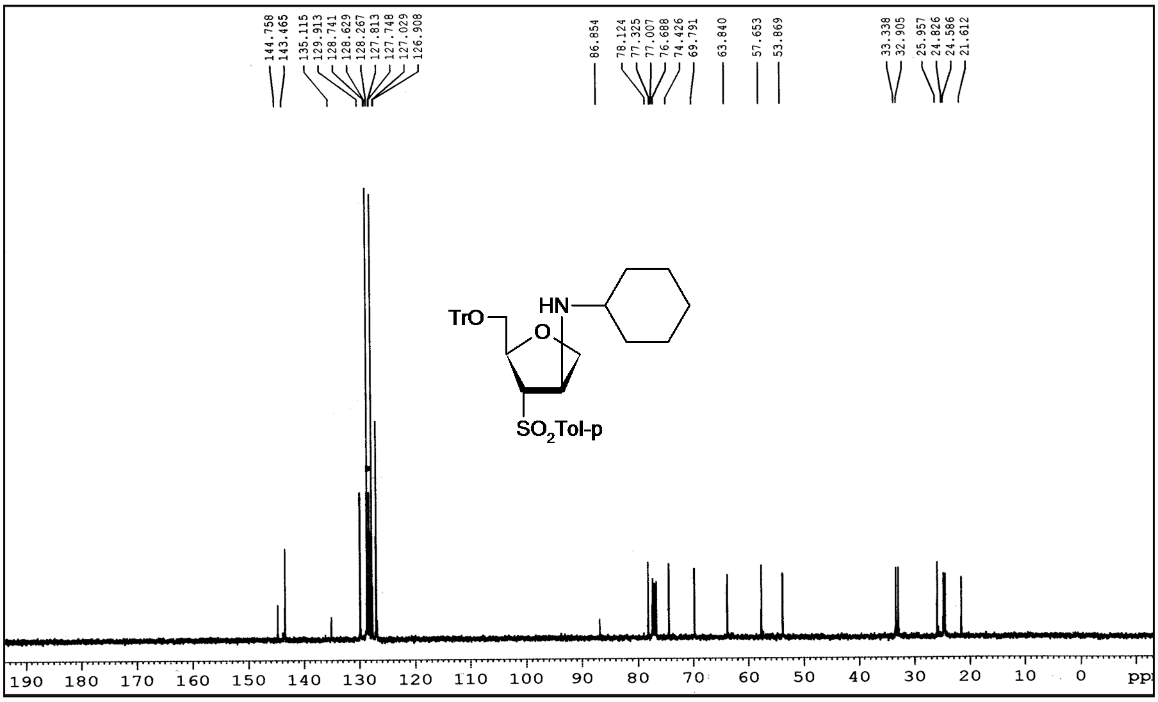

Figure S86. ^{13}C-NMR spectrum of compound **33.**

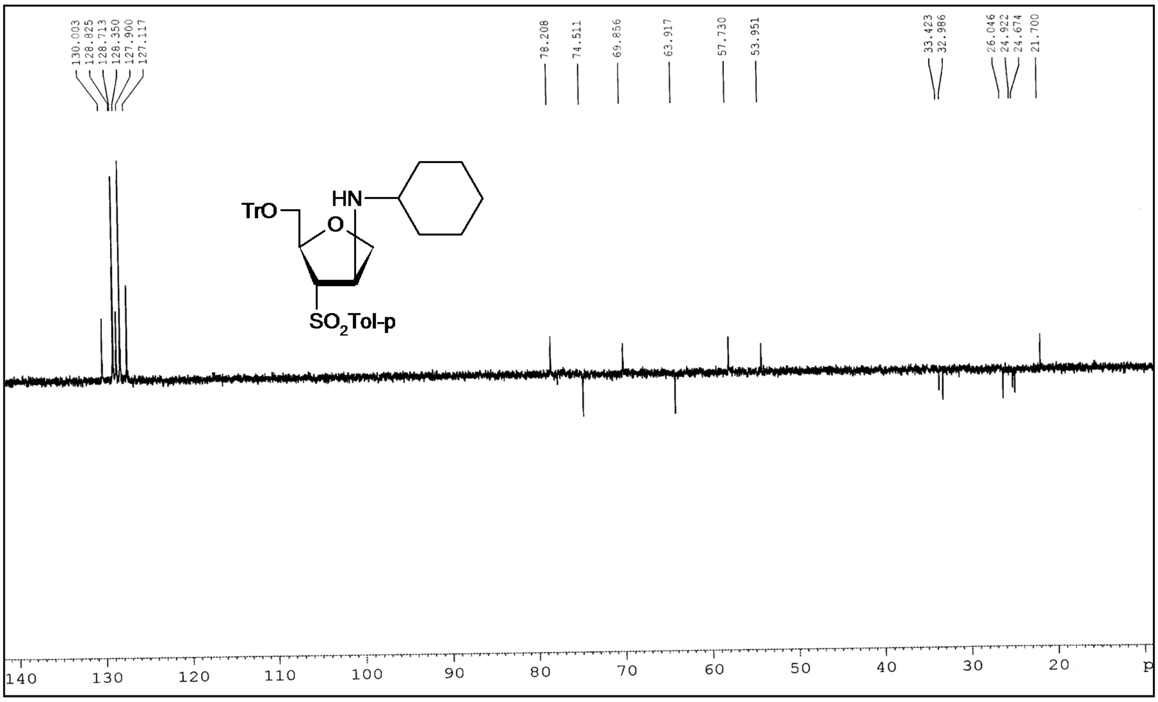

Figure S87. DEPT spectrum of compound **33.**

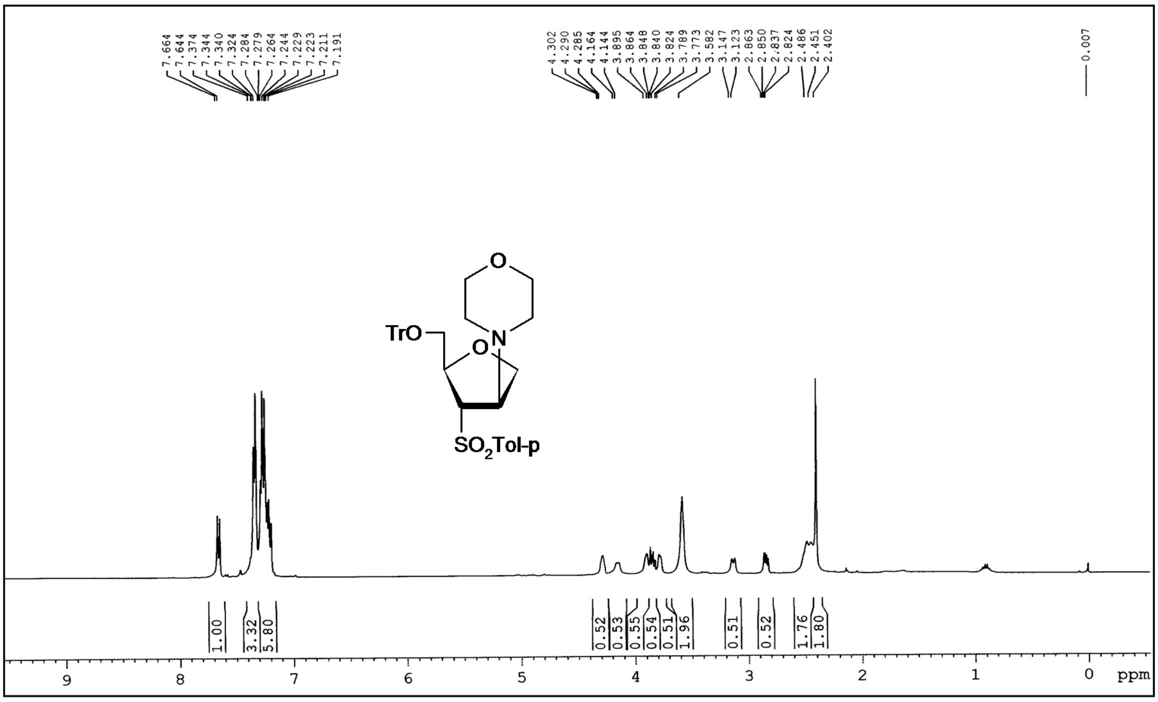

Figure S88. ^{1}H-NMR spectrum of compound **34.**

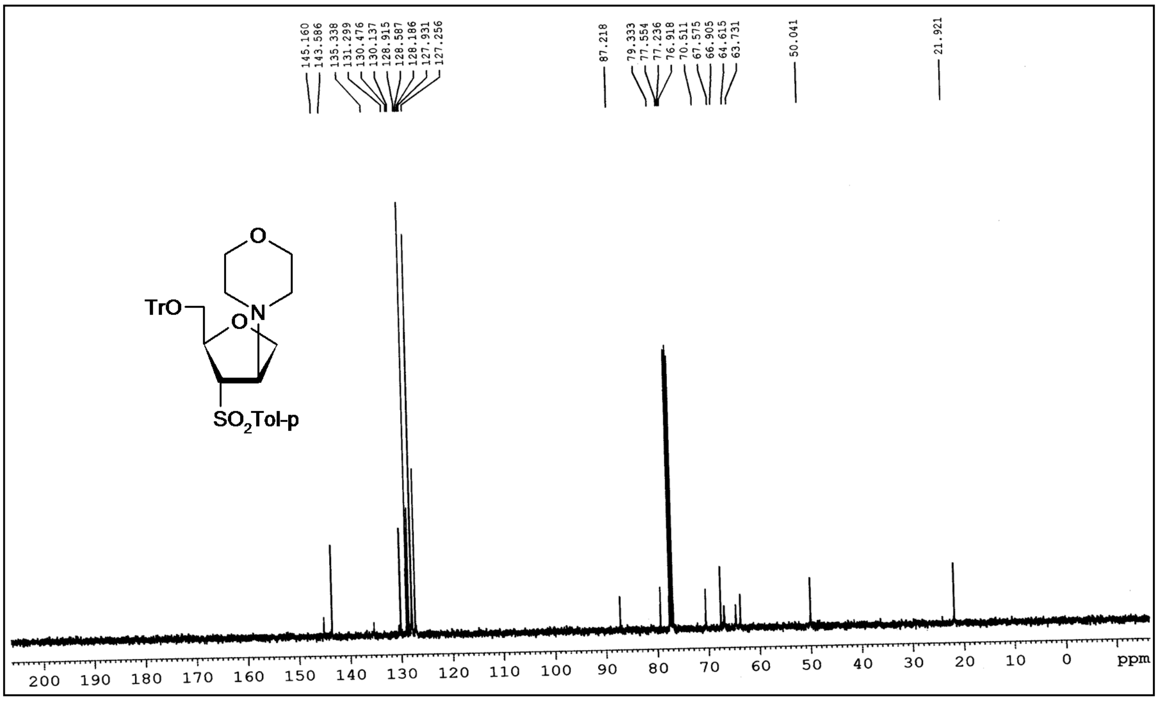

Figure S89. ^{13}C-NMR spectrum of compound **34.**

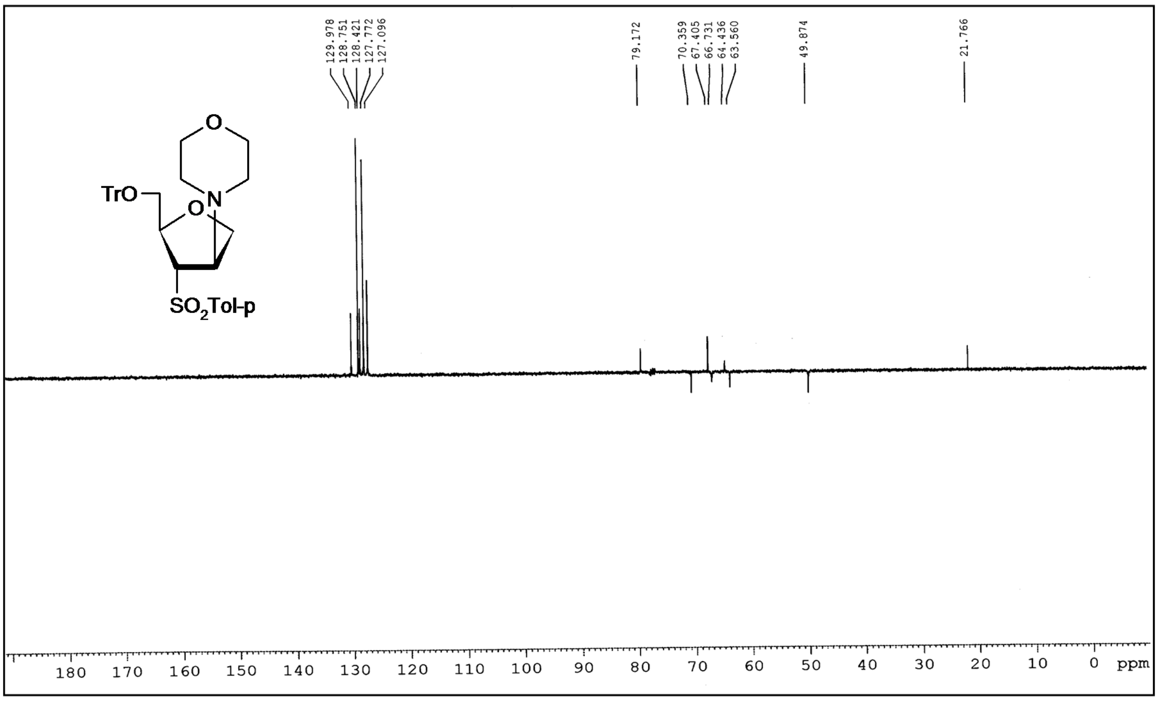

Figure S90. DEPT spectrum of compound **34.**

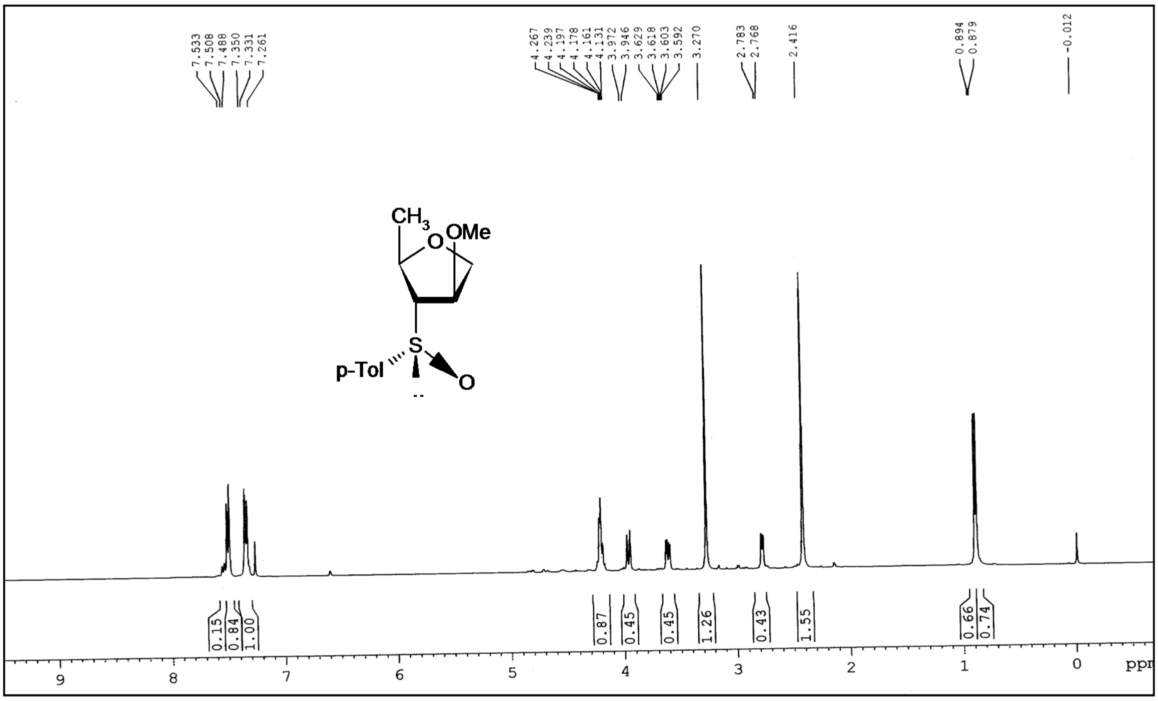

Figure S91. ^{1}H-NMR spectrum of compound **35Ss**.

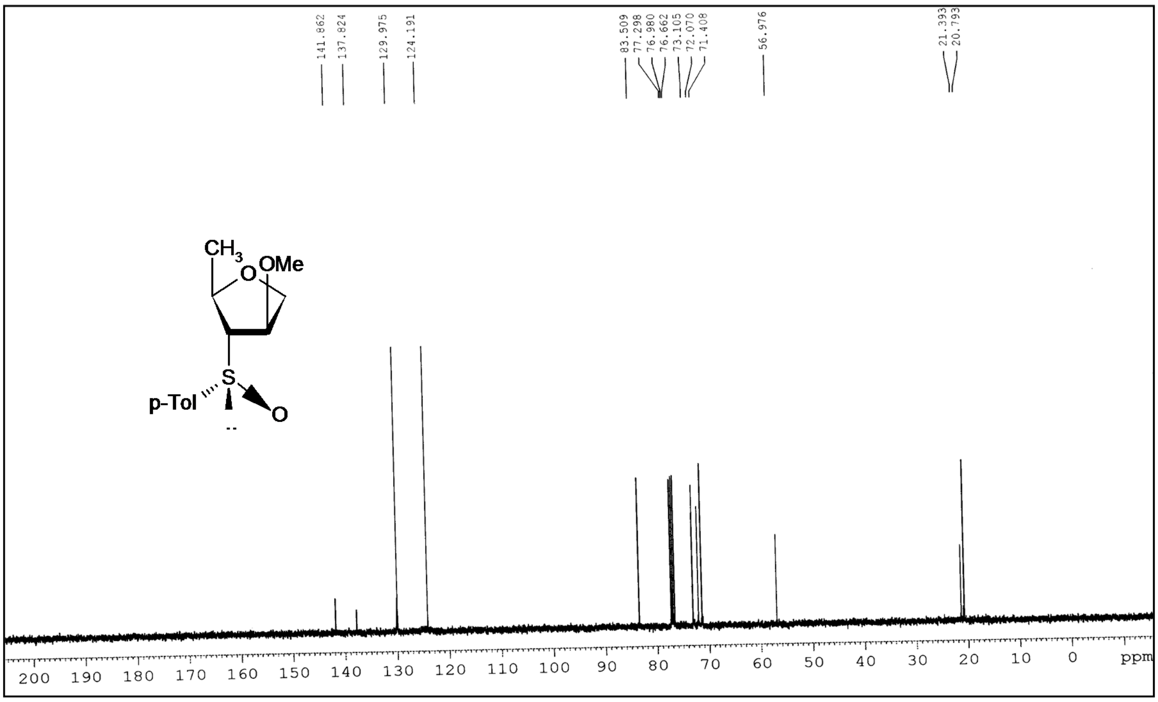

Figure S92. ^{13}C-NMR spectrum of compound **35Ss**.

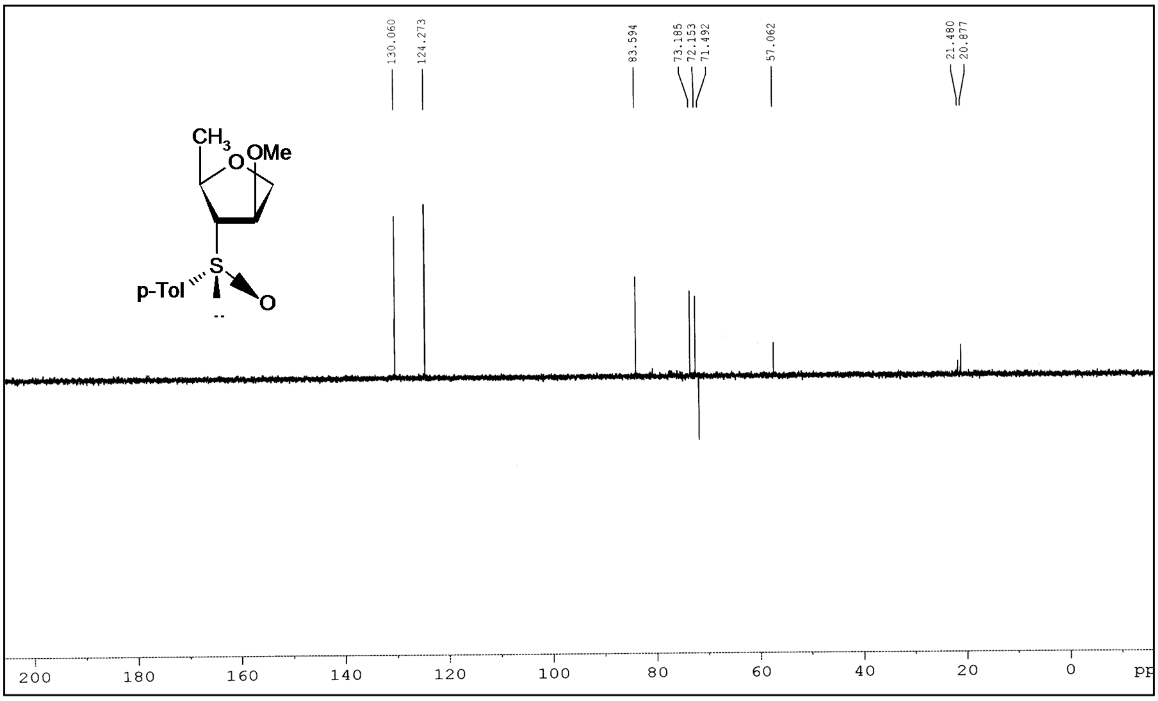

Figure S93. DEPT spectrum of compound **35S**s.

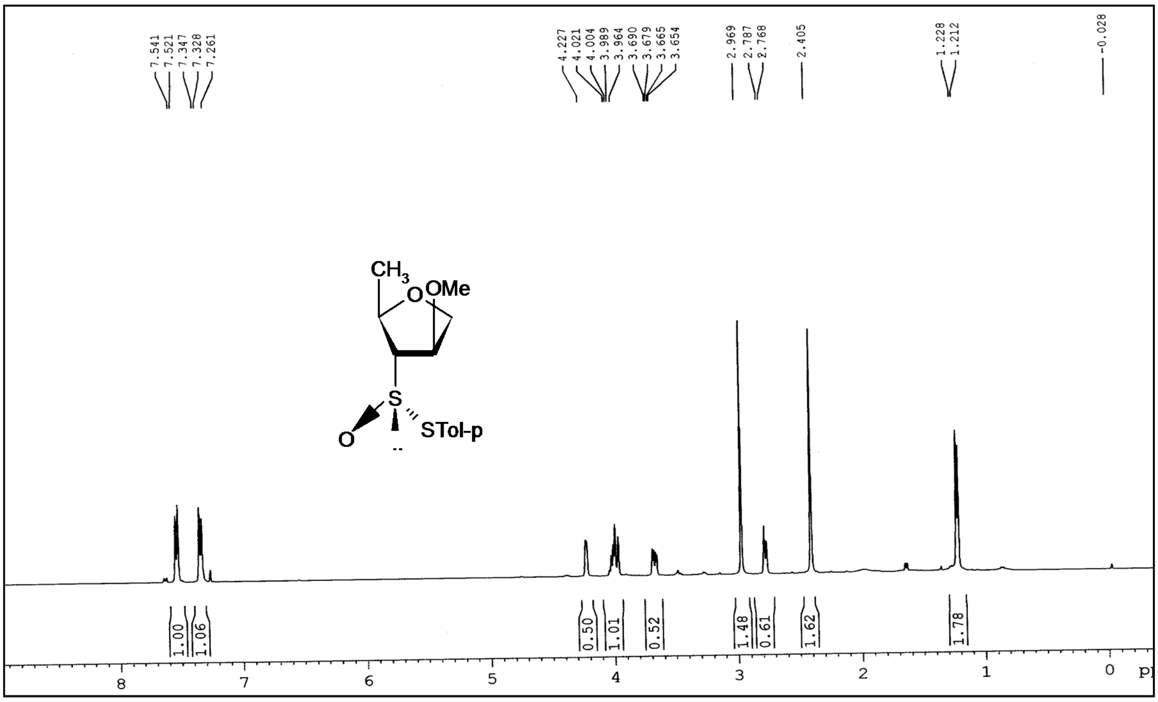

Figure S94. ¹H-NMR spectrum of compound **35R**s.

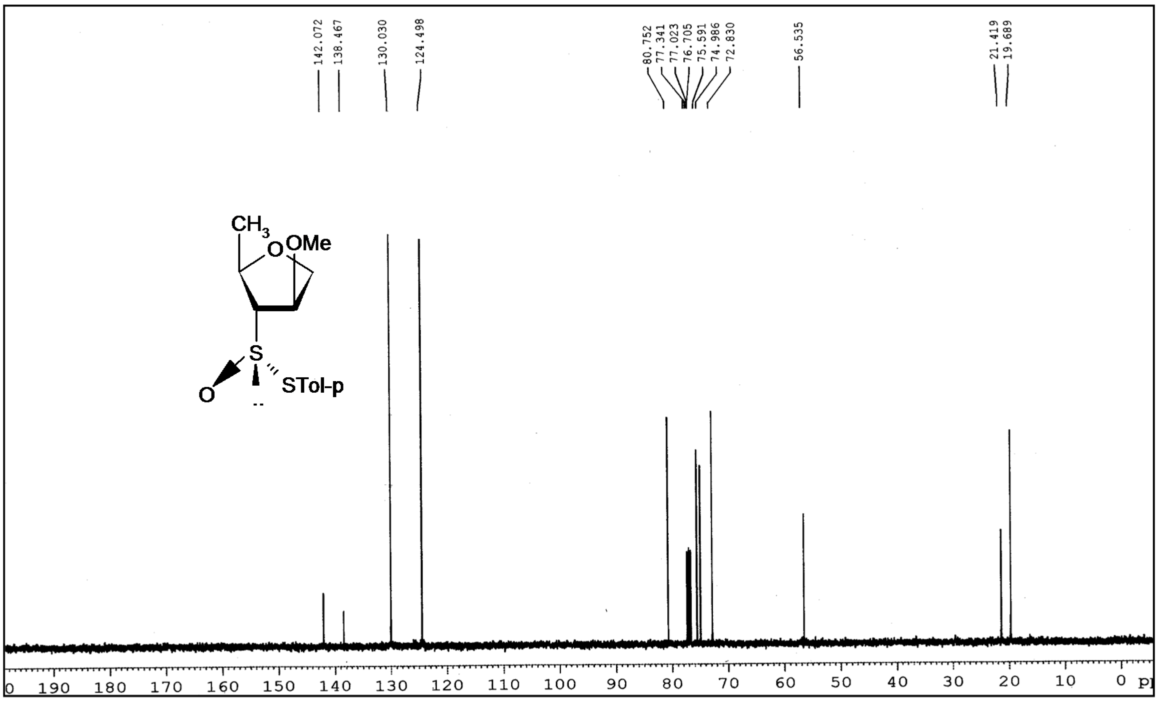

Figure S95. ^{13}C-NMR spectrum of compound **35Rs**.

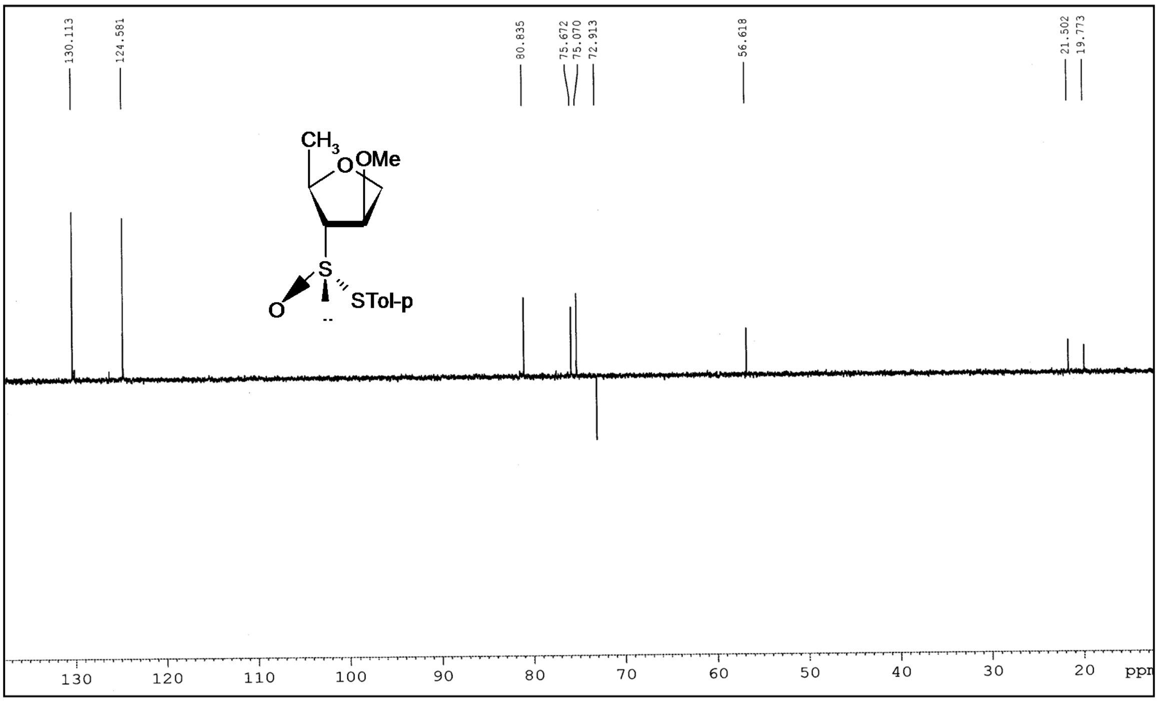

Figure S96. DEPT spectrum of compound **35Rs**.

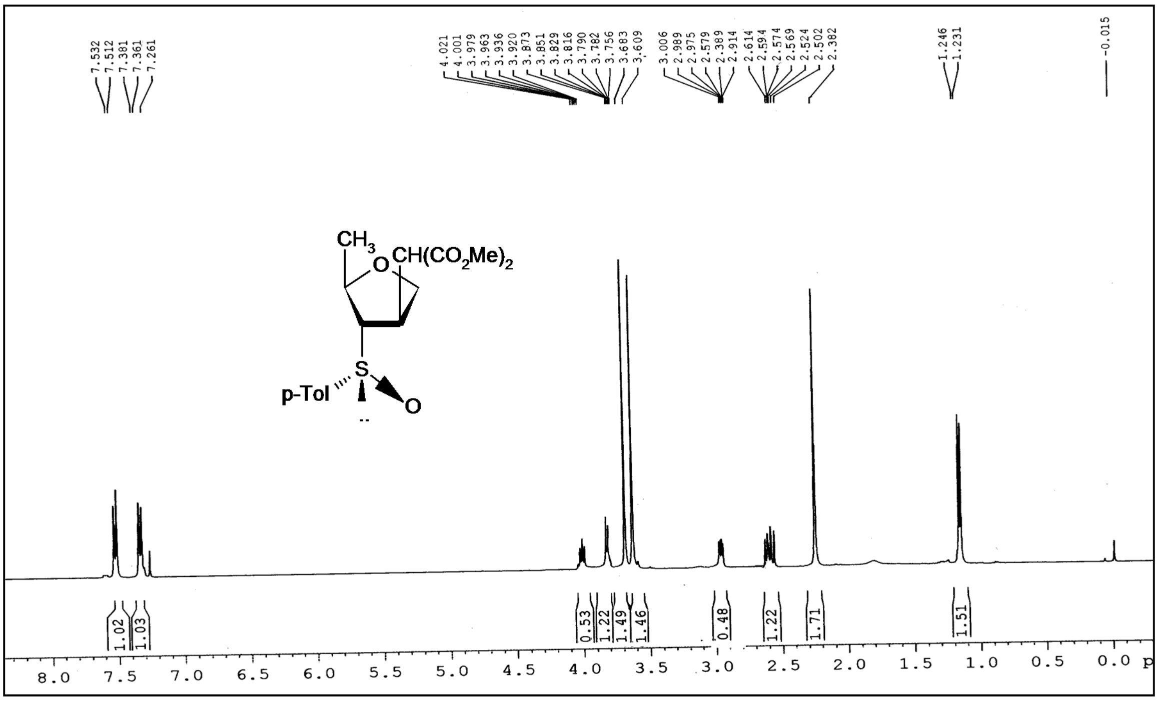

Figure S97. ^{1}H-NMR spectrum of compound **36Ss**.

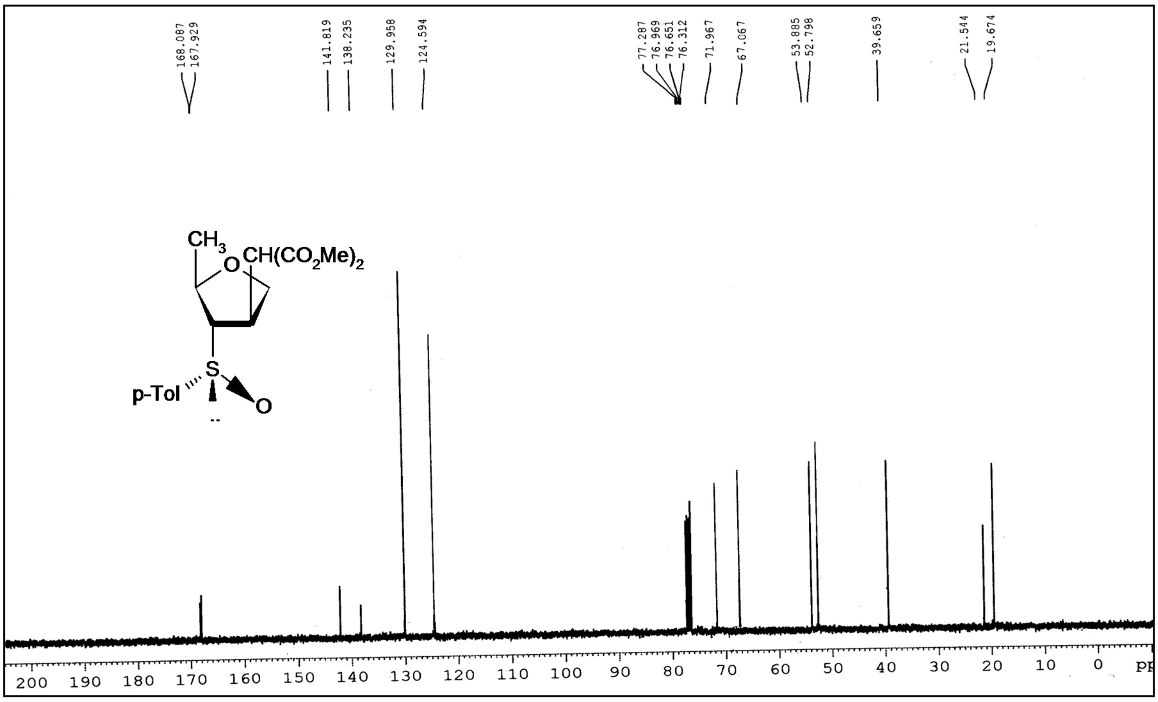

Figure S98. ^{13}C-NMR spectrum of compound **36Ss**.

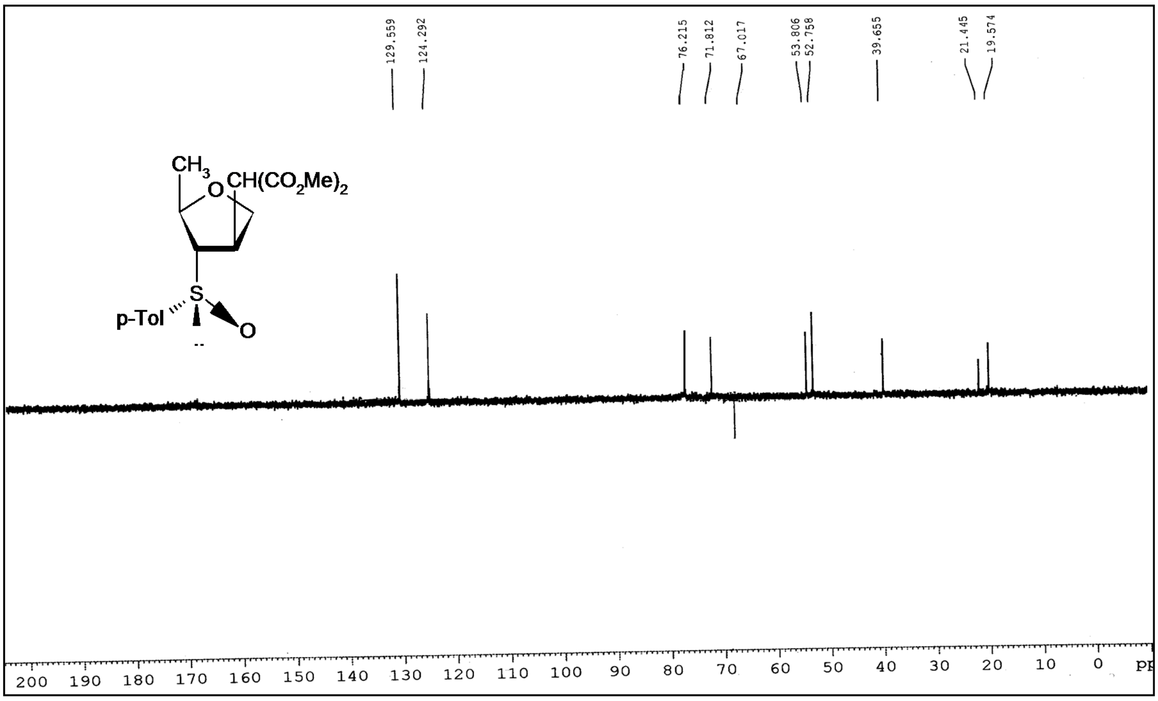

Figure S99. DEPT spectrum of compound **36S**s.

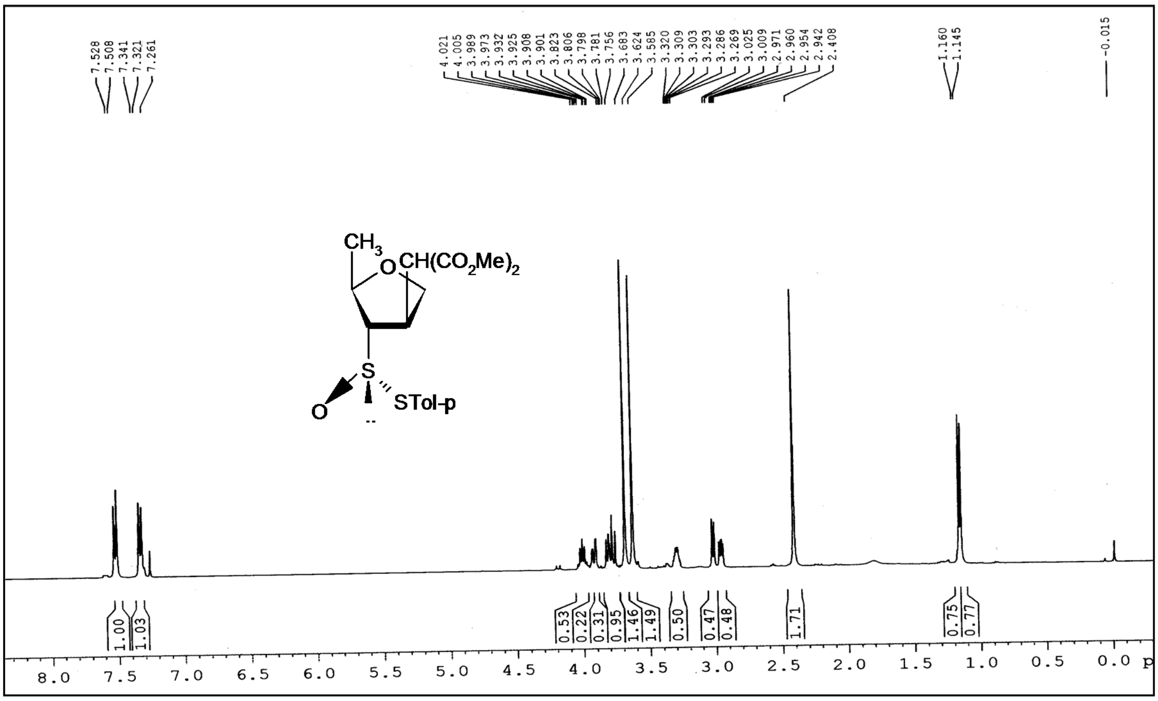

Figure S100. ^{1}H-NMR spectrum of compound **36R**s.

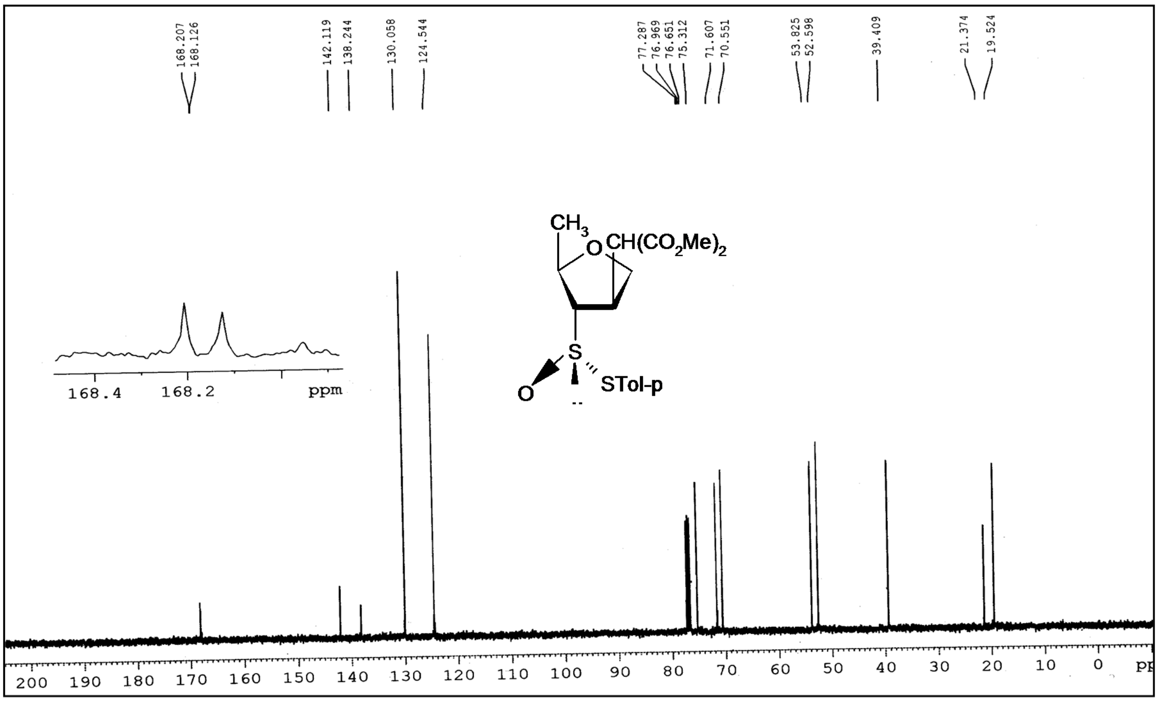

Figure S101. ^{13}C-NMR spectrum of compound **36R**s.

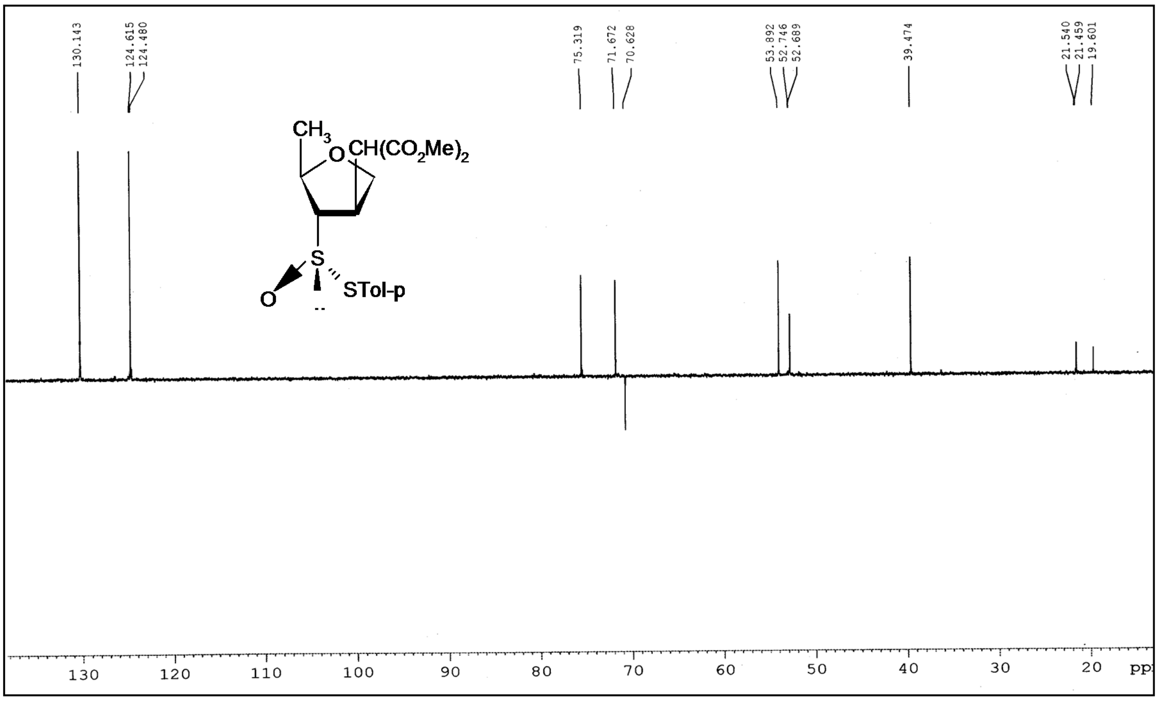

Figure S102. DEPT spectrum of compound **36R**s.

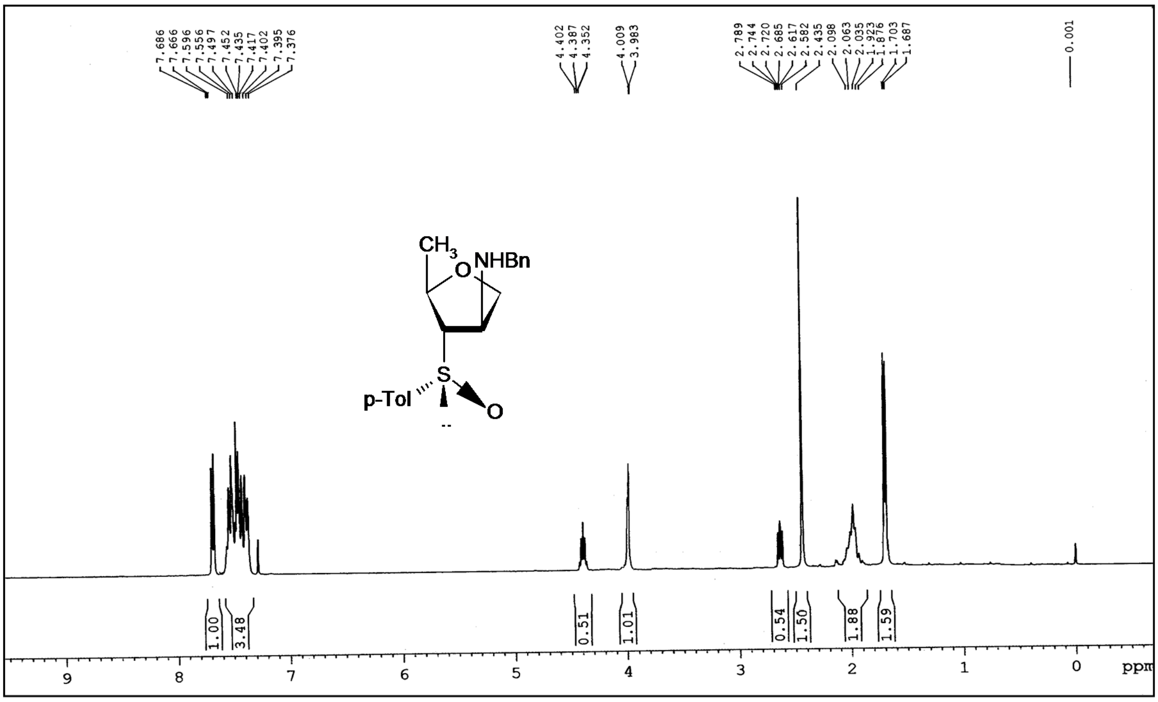

Figure S103. ^{1}H-NMR spectrum of compound **37S**s.

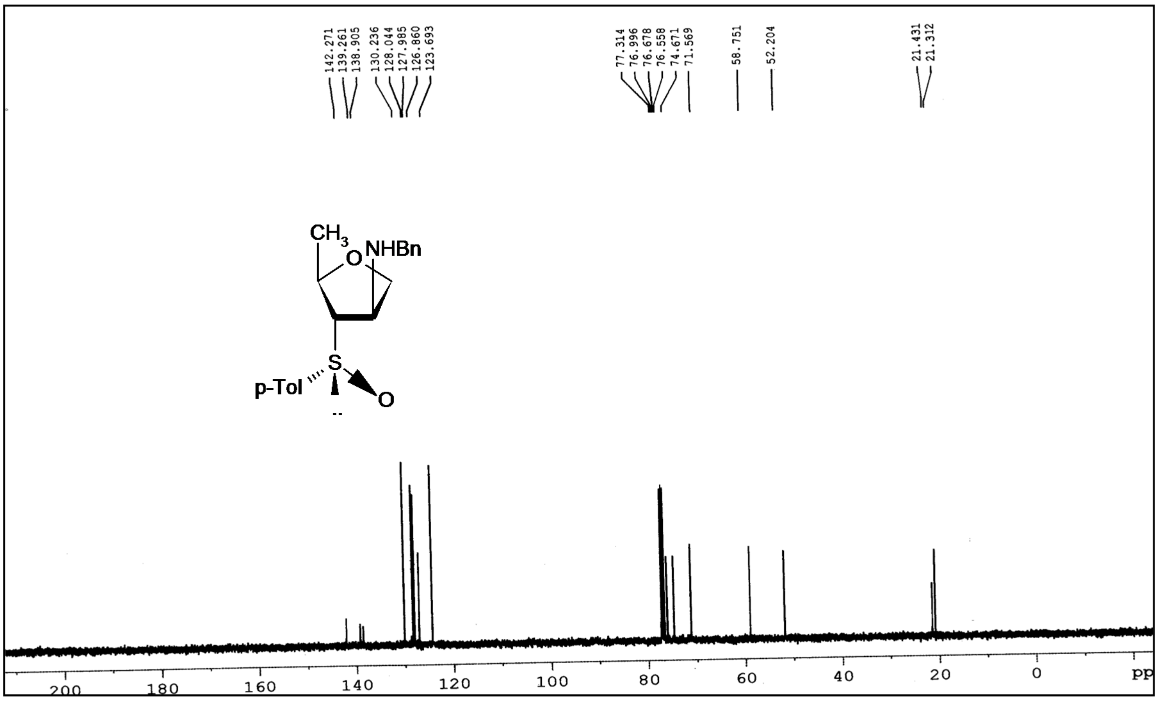

Figure S104. ^{13}C-NMR spectrum of compound **37S**s.

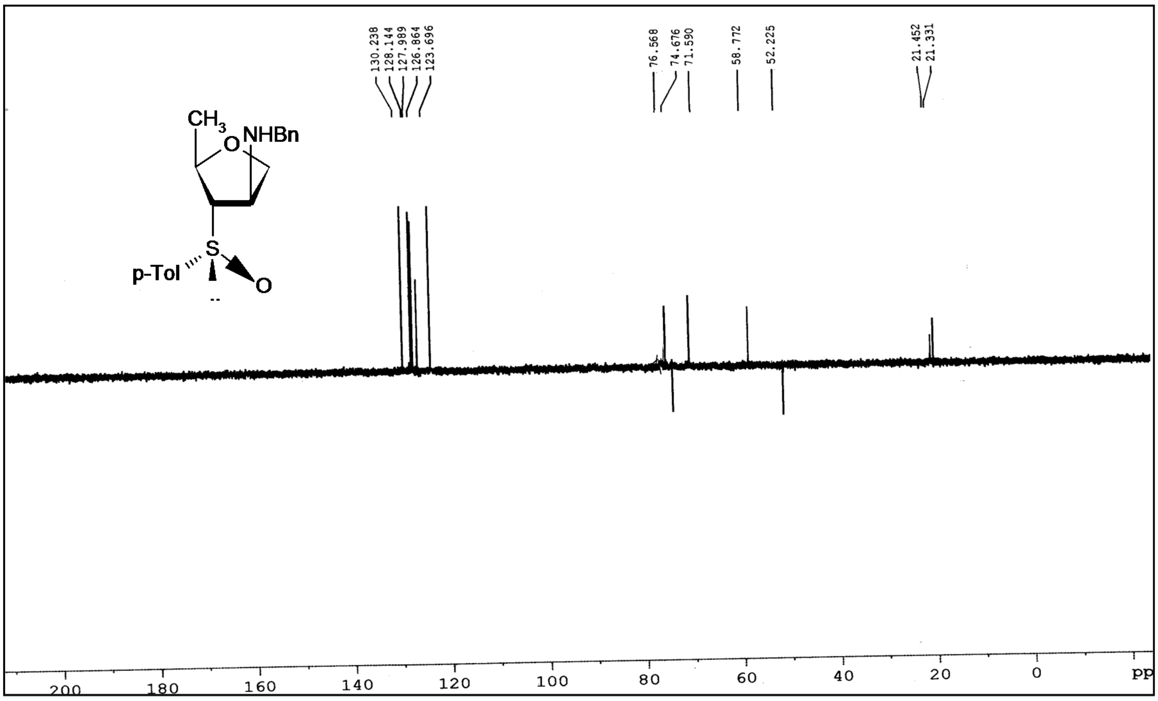

Figure S105. DEPT spectrum of compound **37Ss**.

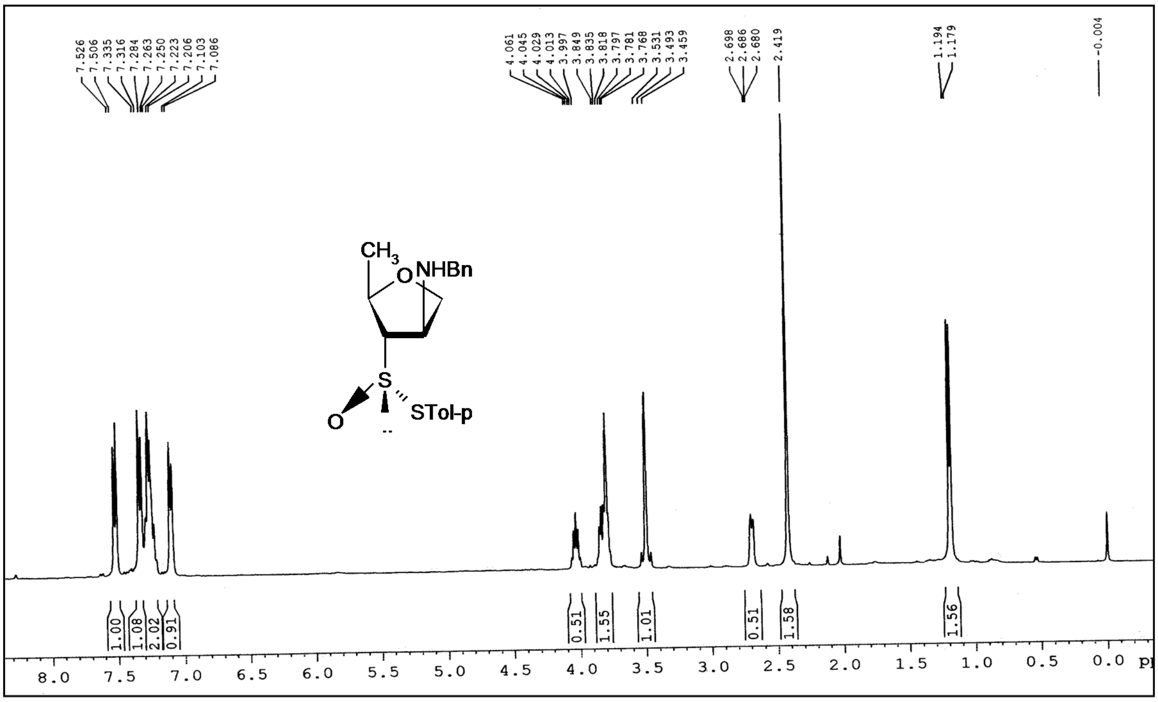

Figure S106. ^{1}H-NMR spectrum of compound **37Rs**.

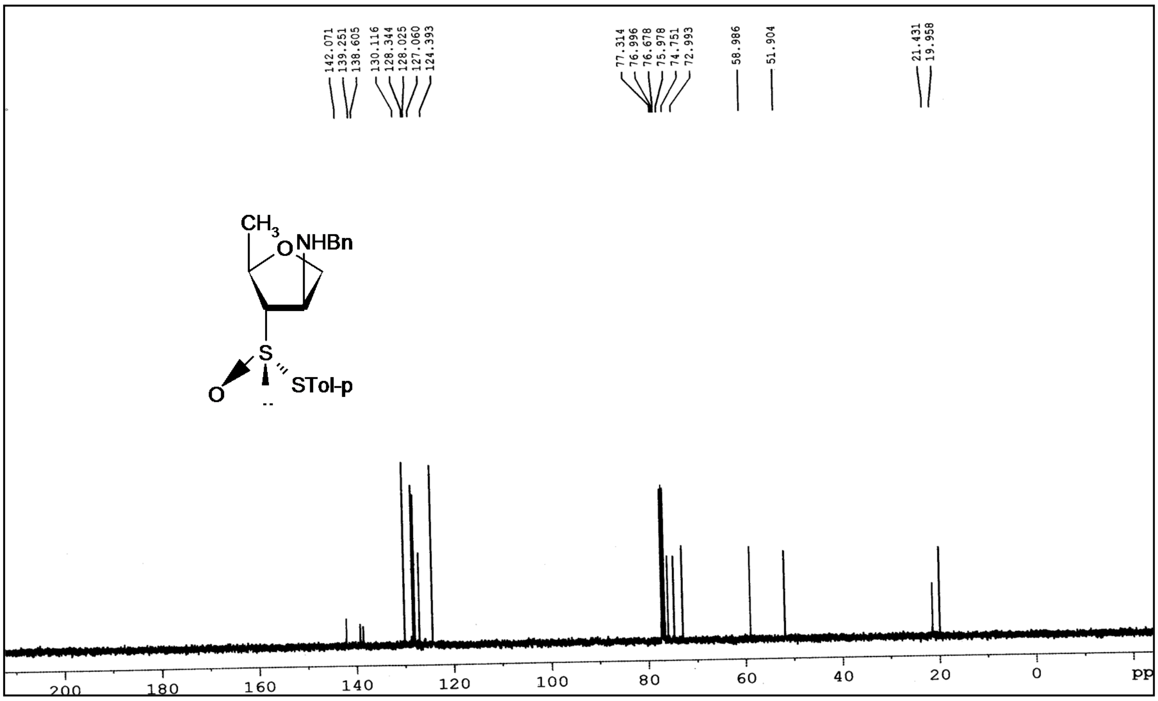

Figure S107. ^{13}C-NMR spectrum of compound **37R**s.

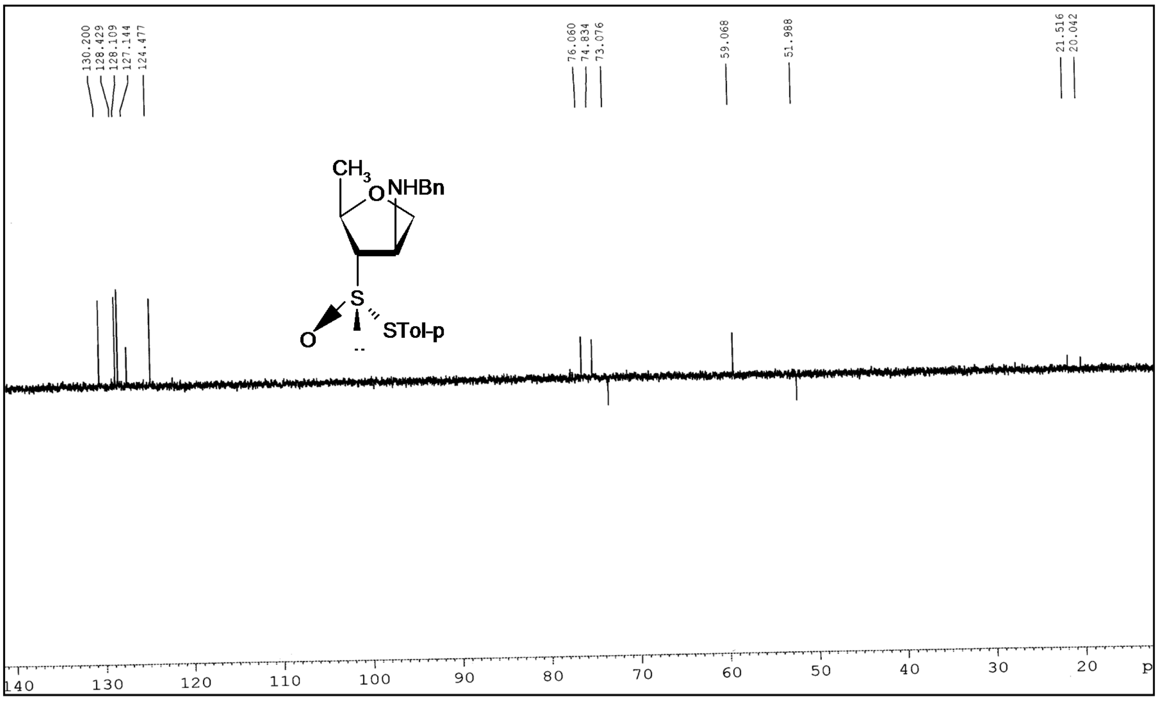

Figure S108. DEPT spectrum of compound **37R**s.

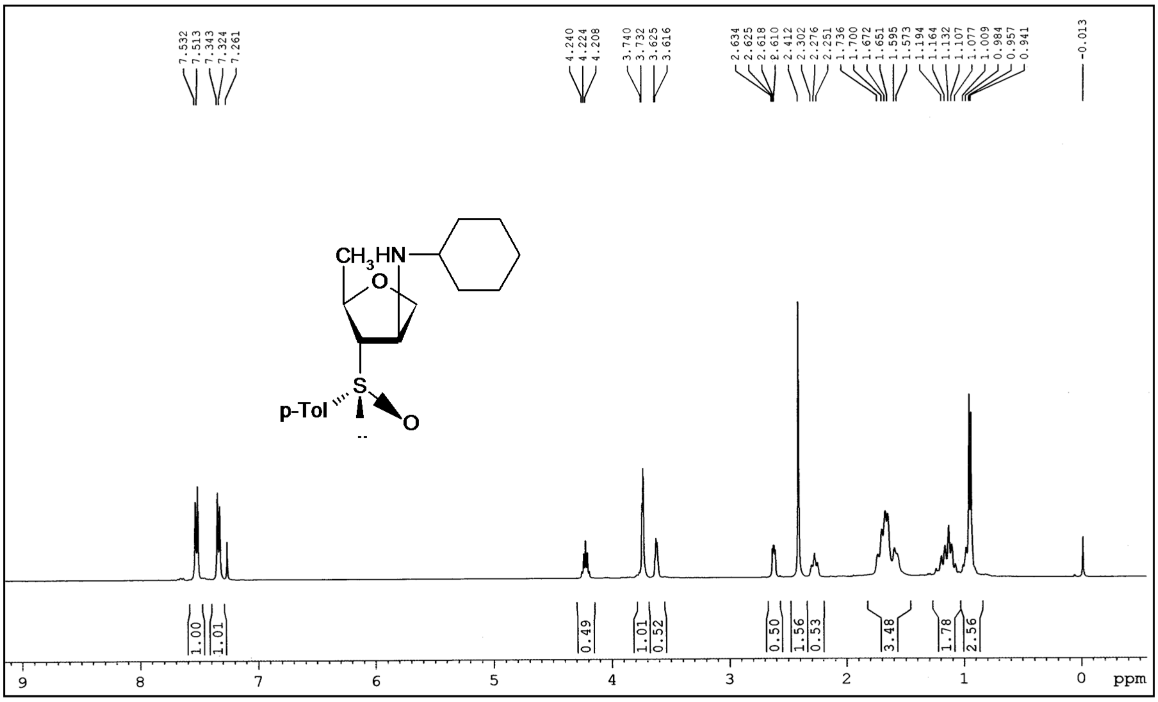

Figure S109. ^{1}H-NMR spectrum of compound **38S**s.

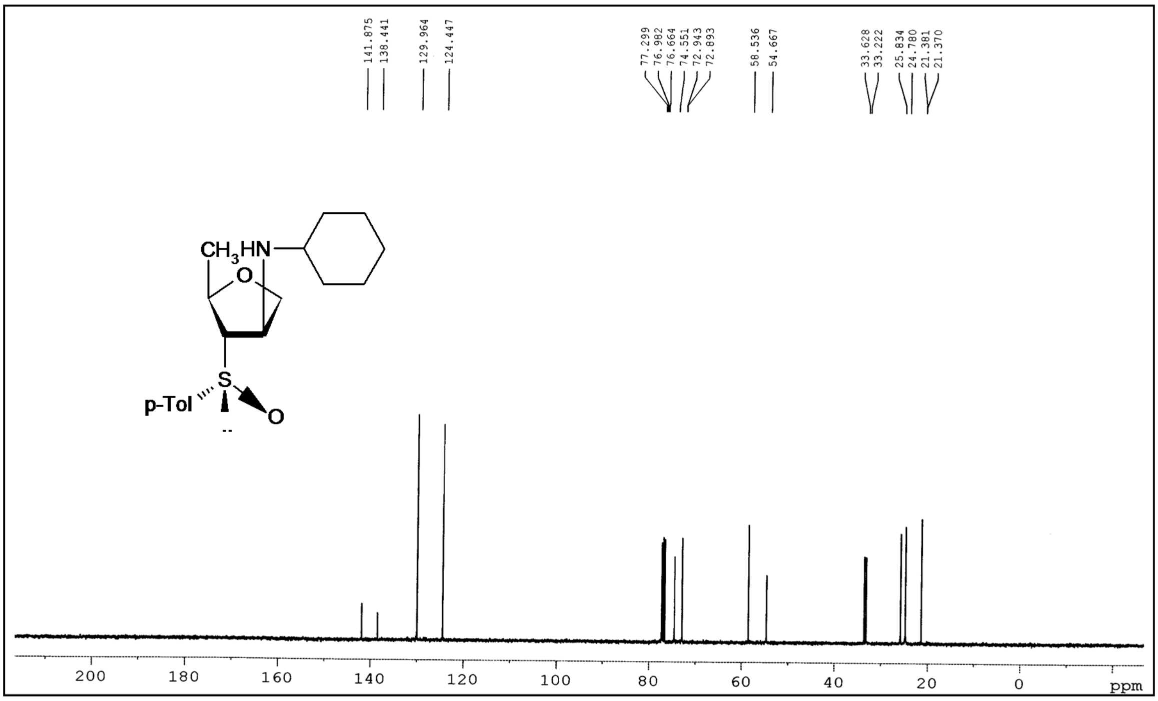

Figure S110. ^{13}C-NMR spectrum of compound **38S**s.

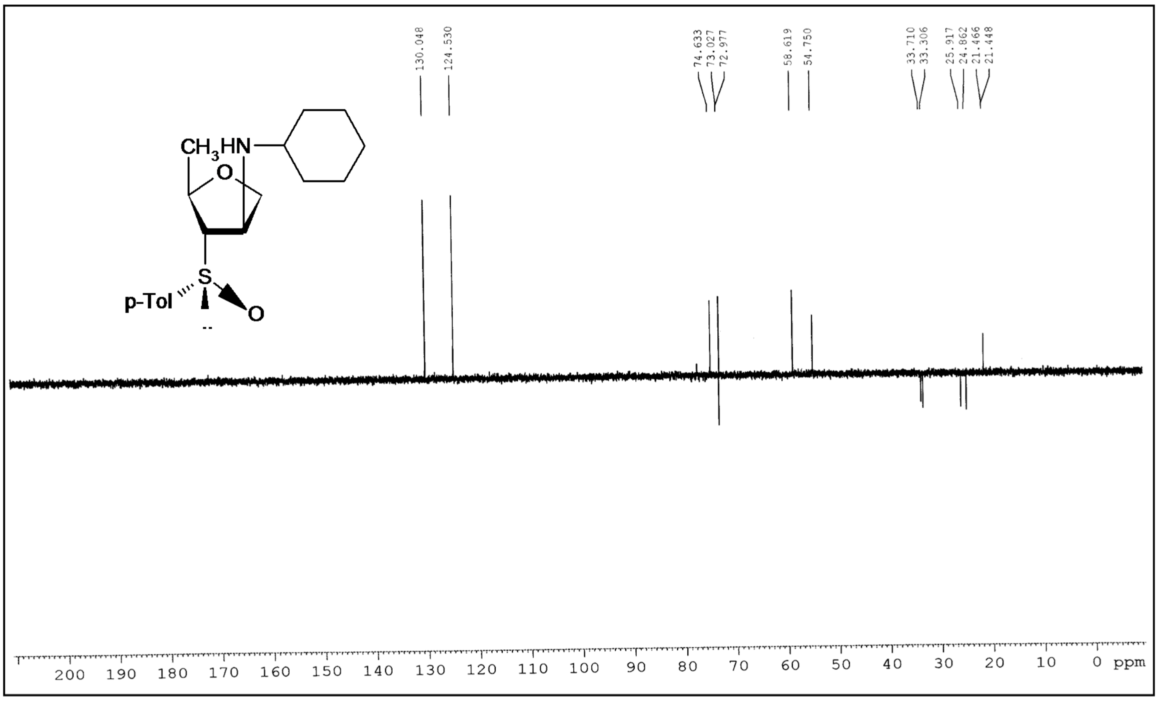

Figure S111. DEPT spectrum of compound **38S**s.

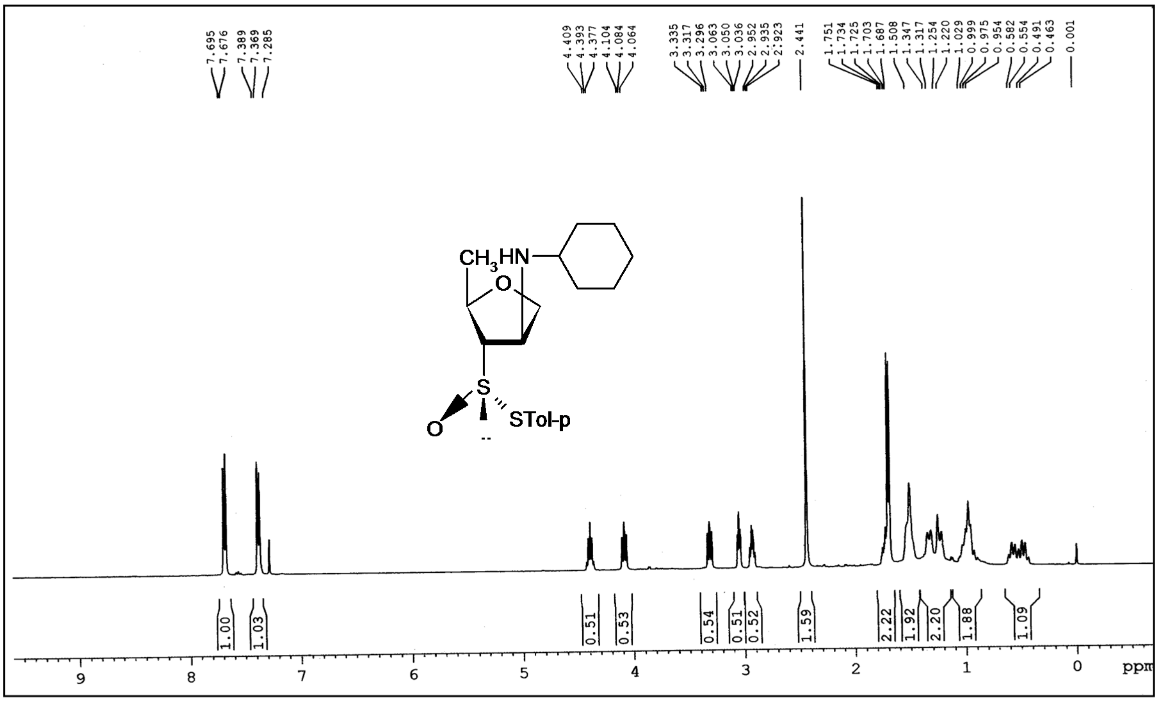

Figure S112. ^{1}H-NMR spectrum of compound **38R**s.

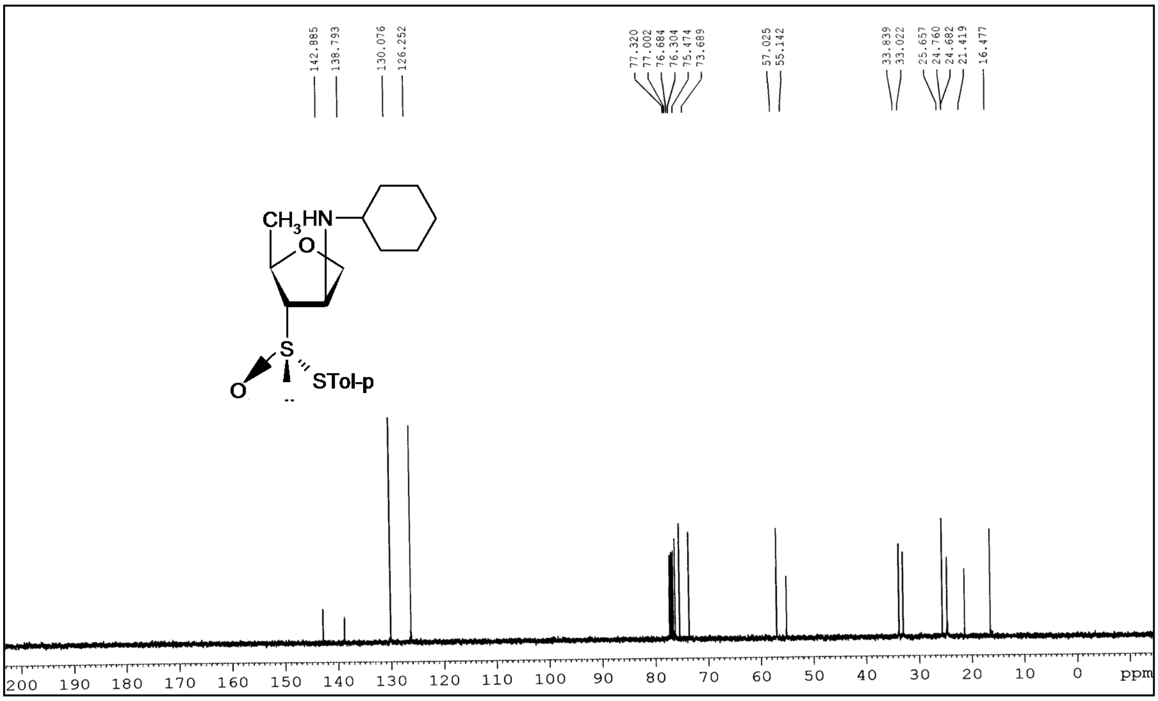

Figure S113. ^{13}C-NMR spectrum of compound **38R**s.

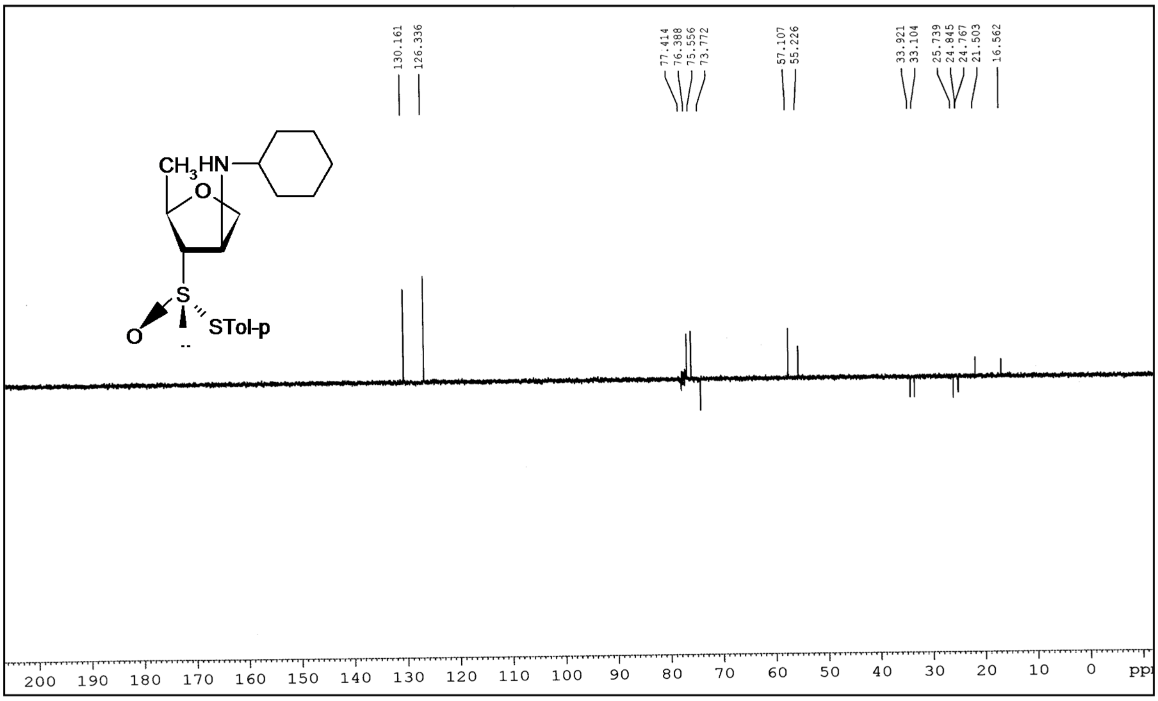

Figure S114. DEPT spectrum of compound **38R**s.
